# Structure‐Based Development of Ultra‐Broad‐Spectrum 3C‐Like Protease Inhibitors

**DOI:** 10.1002/advs.202512342

**Published:** 2025-12-12

**Authors:** Haixia Su, Tianqing Nie, Guofeng Chen, Muya Xiong, Yumin Zhang, Guoqing Wu, Mengyuan You, Hang Xie, Jian He, Yanchao Xiong, Hangchen Hu, Wenfeng Zhao, Minjun Li, Gengfu Xiao, Leike Zhang, Yechun Xu

**Affiliations:** ^1^ School of Pharmaceutical Science and Technology Hangzhou Institute for Advanced Study University of Chinese Academy of Sciences Hangzhou 310024 China; ^2^ State Key Laboratory of Drug Research Shanghai Institute of Materia Medica Chinese Academy of Sciences Shanghai 201203 China; ^3^ State Key Laboratory of Virology Wuhan Institute of Virology Center for Biosafety Mega‐Science Chinese Academy of Sciences Wuhan Hubei 430071 China; ^4^ University of Chinese Academy of Sciences Beijing 100049 China; ^5^ Lingang Laboratory Shanghai 200031 China; ^6^ Hubei Jiangxia Laboratory Wuhan 430200 China; ^7^ School of Physical Science and Technology ShanghaiTech University Shanghai 201210 China; ^8^ Shanghai Synchrotron Radiation Facility Shanghai Advanced Research Institute Chinese Academy of Sciences Shanghai 201204 China

**Keywords:** 3C‐like protease, conservation analysis of the binding pocket, co‐crystal structure, structure‐based drug design, ultra‐broad‐spectrum inhibitors

## Abstract

Recurrence of coronavirus outbreaks and zoonotic origins of human coronaviruses underscore the importance of developing pan‐coronavirus antivirals. The highly conserved 3C‐like protease (3CL^pro^) in coronaviruses, together with the well‐established druggability, makes it an ideal target for broad‐spectrum antiviral therapeutics. Here, the inhibitory activity of approved 3CL^pro^ inhibitors, including nirmatrelvir, ensitrelvir, and simnotrelvir, against fifteen 3CL^pro^s is first reported by enzymatic assays. Despite their potent inhibition toward 3CL^pro^s of β‐CoVs, these inhibitors show reduced potency against 3CL^pro^s from the other three genera, particularly against two newly identified human coronaviruses (α‐CCoV‐HuPn‐2018 and δ‐PDCoV). In this context, continued efforts in structure‐based optimization of nirmatrelvir lead to the identification of compound **8** that potently inhibits a panel of 32 3CL^pro^s across all subgenera (IC_50_s: 19–146 nm), with an IC_50_ value of 61 and 81 nm against α‐CCoV‐HuPn‐2018 and δ‐PDCoV 3CL^pro^s, respectively. Moreover, it effectively inhibits nirmatrelvir‐resistant 3CL^pro^ mutants and demonstrates broad‐spectrum antiviral efficacy in cells. These findings suggest an important rule that a small, non‐cyclic P2 segment and a P4 segment with a suitable size are preferred by the design of ultra‐broad‐spectrum 3CL^pro^ inhibitors, and provide a proof‐of‐concept guide for developing broad‐spectrum antivirals as potential pan‐CoV therapeutics.

## Graphical abstract

This study provides an in‐depth analysis of the substrate binding pocket of 3CL^pro^s across all coronavirus species using bioinformatics and structural insights, revealing the critical impact of S2/S4 subsite diversity on the broad‐spectrum activity of approved therapeutics. Structure‐based optimization of nirmatrelvir led to the identification of compound **8**, an ultra‐broad‐spectrum 3CL^pro^ inhibitor that potently inhibits a panel of 32 3CL^pro^s across all subgenera.

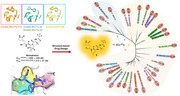

## 1 Introduction

Coronaviruses (CoVs, subfamily *Orthocoronavirinae*, family *Coronaviridae*, suborder *Cornidovirineae*, order *Nidovirales*) are a large family of enveloped, positive‐sense, single‐stranded RNA viruses that infect animals as well as humans to cause a wide range of diseases. For example, there has been a recurrence of CoV outbreaks in recent decades, as illustrated by severe acute respiratory syndrome (SARS) caused by SARS‐CoV in 2003, Middle East respiratory syndrome (MERS) caused by MERS‐CoV in 2012, and, most notably, the ongoing COVID‐19 pandemic caused by SARS‐CoV‐2. CoVs are classified into four highly divergent genera (α‐, β‐, γ‐, and δ‐CoV) and 26 subgenera. Seven human coronaviruses (HCoVs) from two genera have been well recognized. HCoV‐HKU1, HCoV‐OC43, SARS‐CoV, MERS‐CoV, and SARS‐CoV‐2 belong to the β‐CoV genus, while HCoV‐229E and HCoV‐NL63 are in the α‐CoV genus. These HCoVs are believed to be transmitted from animals, of which bats are the major source, suggesting that these zoonotic viruses are capable of crossing species barriers to infect humans.^[^ ^1^ ^]^ As further evidence of this, porcine δ‐CoV (PDCoV)^[^ ^2^ ^]^ and a novel canine‐feline recombinant α‐CoV (CCoV‐HuPn‐2018)^[^ ^1^ ^]^ have been recently found to jump from nonhuman hosts into human beings, resulting in the number of HCoVs up to nine. It is thus speculated that recurrent cross‐species transmissions, along with a variety of zoonotic CoVs circulating in wildlife, greatly increase the risk of the emergence of new HCoVs in different genera of CoVs. In this case, discovery and development of pan‐coronavirus antivirals that possess the cross‐inhibiting activity against α‐, β‐, γ‐, and δ‐CoVs provides a promising therapeutic option to tackle persistent mutation and adaptation of zoonotic CoVs.

CoVs possess conserved genomic elements encoding viral proteins that are essential for viral replication and are pivotal targets for developing broad‐spectrum antiviral therapeutics. Among them, 3C‐like protease (3CL^pro^) is a highly conserved cysteine proteinase responsible for the cleavage of two viral polyproteins (pp1a and pp1ab) to release non‐structural proteins for viral replications.^[^ ^3^ ^]^ Regarding the sequence and structural conservation, we previously reported that the averaged sequence identity between every two of 12 3CL^pro^s is 51%, with the highest value (96%) for SARS‐CoV and SARS‐CoV‐2 3CL^pro^s, while overall crystal structures of 12 3CL^pro^s are nearly identical in spite of the sequence variation.^[^ ^3^ ^]^ Furthermore, unlike the spike glycoprotein, which is vulnerable to mutations,^[^ ^4^ ^]^ 3CL^pro^ and particularly its substrate binding site (S1'‐S4) are highly conserved in prevalent CoVs like SARS‐CoV‐2 and its variants.^[^ ^3^ ^]^ Accordingly, the highly conserved 3CL^pro^ represents an attractive drug target for the development of small molecule inhibitors with antiviral activity across different genera of CoVs. In this context, a comprehensive *in‐silico* investigation to further explore conserved characteristics of the substrate binding pocket across 3CL^pro^s of 26 subgenera of CoVs is crucial to design and optimize the broad‐spectrum inhibitors.

The remarkable druggability of 3CL^pro^ has been evidenced by the approval of its inhibitors, including nirmatrelvir (PF‐07321332),^[^ ^5^ ^]^ ensitrelvir (S‐217622),^[^ ^6^ ^]^ and simnotrelvir (SIM0417/SSD8432),^[^ ^7^ ^]^ for the treatment of COVID‐19. Moreover, our structure‐based development of simnotrelvir from boceprevir, an oral drug targeting HCV NS3/4A serine protease, indicates that the development of new viral protease inhibitors based on marketed drugs offers a cutting‐edge solution to efficiently find therapeutic interventions against emerging highly pathogenic viruses.^[^ ^7^ ^]^ Nevertheless, the broad‐spectrum activity of these approved inhibitors against 3CL^pro^s of four genera of CoVs, in particular two newly characterized HCoVs (PDCoV and CCoV‐HuPn‐2018), has not yet been determined. In the present study, we carried out the broad‐spectrum inhibitory activity measurement of the approved compounds, showing that they can potently inhibit 3CL^pro^s of β‐CoVs but not the other genera of CoVs. This prompts us to further develop pan‐genus CoV 3CL^pro^ inhibitors. Fortunately, the knowledge gained from our comprehensive bioinformatic analysis on 59 3CL^pro^s across all CoV species and the inhibitor binding modes revealed by co‐crystal structures of PDCoV, CCoV‐HuPn‐2018, and SARS‐CoV‐2 3CL^pro^s lays a solid foundation for structural optimization of nirmatrelvir to yield novel ultra‐broad‐spectrum inhibitors that ultimately show potent inhibitory activity against 3CL^pro^s of 26 subgenera of four genera, as well as nine HCoV 3CL^pro^s. To the best of our knowledge, this new class of inhibitors, exemplified by compound **8**, possess the broadest spectrum of inhibitory activity among all protease inhibitors.

## 2 Results

### 2.1 Conserved Features of the Substrate Binding Pocket of 3CL^pro^s

**Figure 1:**
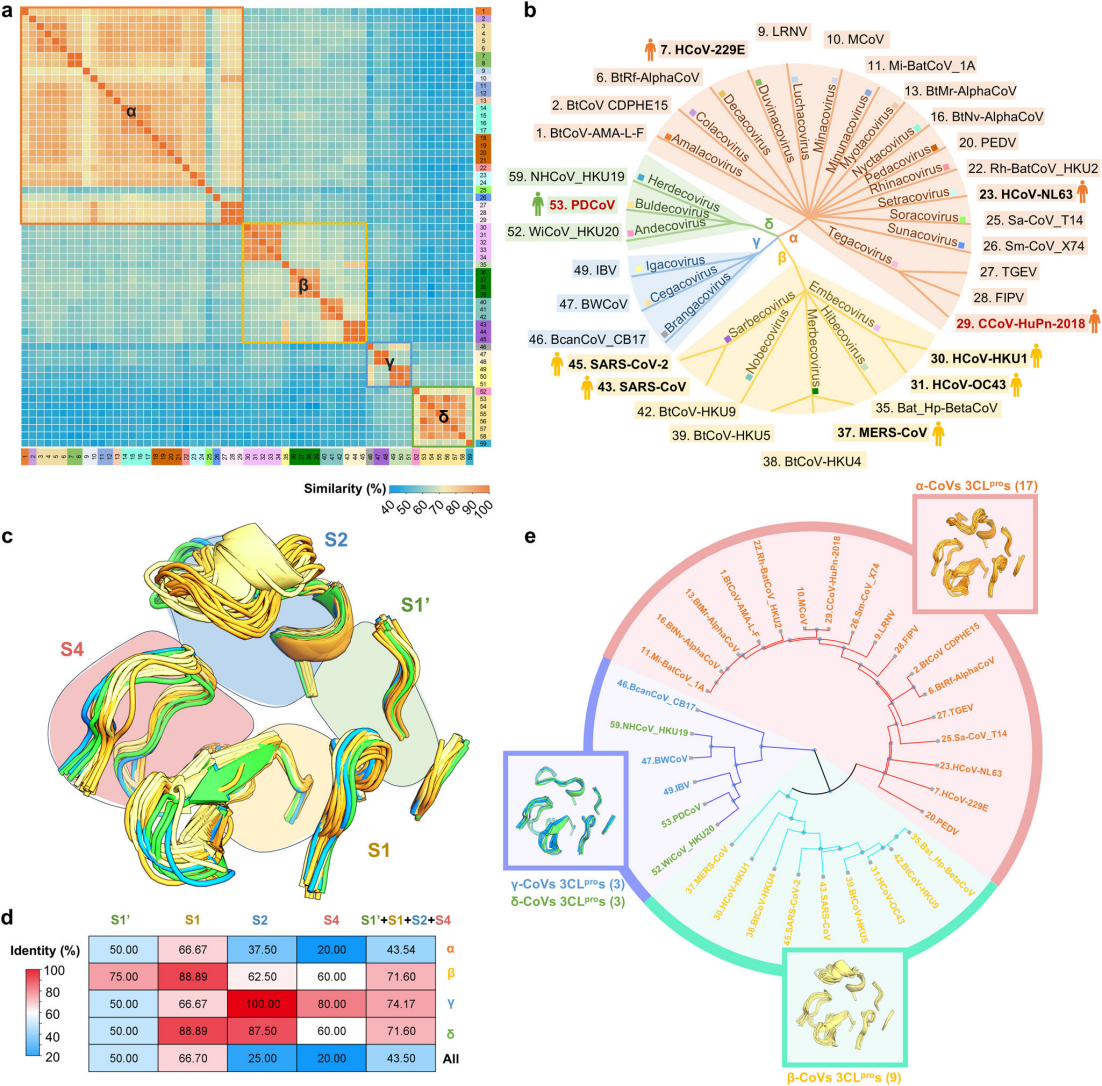
Conservation analysis of sequences, 3D structures, and substrate binding pockets of CoV 3CL^pro^s. a) Pairwise sequence similarity matrix depicting the sequence similarities among 59 CoV 3CL^pro^s across 26 subgenera. The heatmap colors represent the degree of similarity. Different subgenera of CoVs are distinguished by different colors; four genera of CoVs are boxed by different colors: orange (α), yellow (β), blue (γ), and green (δ). b) The taxonomy of picked 32 CoVs, including exemplar CoVs of all 26 subgenera, human CoVs (HCoVs, marked with a man icon), and other CoVs of concern. c) Structure alignments of substrate binding pockets of 32 3CL^pro^s based on available crystal structures and AlphaFold2‐predicted structures. Four subsites are circled by different colors: light green (S1ʹ), light yellow (S1), light blue (S2), and rose red (S4). d) Sequence identity heatmap indicating the conservation of residues at four subsites (S1ʹ, S1, S2, and S4) of 3CL^pro^s within each genus and 32 3CL^pro^s together. Heatmap colors represent the degree of identity. e) The structural dendrogram of 32 3CL^pro^s based on a pairwise structure similarity matrix, and structure alignments of four subsites for each of three clusters are provided in boxes.

In accordance with the taxonomic classification established by the International Committee on Taxonomy of Viruses (ICTV) on June 7, 2023, the *Orthocoronavirinae* subfamily comprises a total of 52 species that belong to 26 subgenera of four genera, contributing the vast majority of members to the *Coronaviridae* family. We incorporated 56 exemplar CoVs of 52 species enumerated in ICTV, another CoV of concern (FIPV), and two recently identified but unclassified HCoVs (PDCoV and CCoV‐HuPn‐2018) into a total of 59 CoVs for sequence conservation analysis of 3CL^pro^s (**Figure** **1**a,b; Figure S1, Supporting Information). In the pairwise alignment of these 59 CoV 3CL^pro^s, the averaged sequence similarity is 65% while a higher level of sequence similarity is observed for 3CL^pro^s in every genus, with an averaged sequence similarity of 80%, 72%, 76%, and 81% for α‐, β‐, γ‐, and δ‐genus, respectively (Figure 1a). Moreover, this value reaches 92% for 3CL^pro^s within a subgenus (Figure 1a). Overall, the substantially high sequence similarity of 3CL^pro^ within and across genera of the *Orthocoronavirinae* subfamily demonstrates the potential of 3CL^pro^ as a target for the development of pan‐genus anti‐CoV agents.

To strengthen further the finding that 3CL^pro^ is highly conserved across the *Orthocoronavirinae* subfamily, we turned our attention to exploring conserved features at the substrate binding pocket to which the inhibitors bind. Given the substantially high sequence similarity of 3CL^pro^s within a subgenus, we selected 32 CoVs, including one exemplar CoV per subgenus and the remaining HCoVs (Figure 1b; Figure S1, Supporting Information), for analyzing sequence identity of amino acids at the substrate binding pocket, that is mainly consists of S1'/S1/S2/S4 subsites. As the S3 subsite is a solvent‐exposed region and the inhibitors typically do not interact with it, we excluded the S3 subsite from the following analysis. The results demonstrate remarkably high conservation within the β‐, γ‐, or δ‐genus CoV 3CL^pro^s, with surpassing 50% identity for each subsite (Figure 1c,d; Figure S2, Supporting Information). However, for the α‐CoV 3CL^pro^s, while the amino acids at S1' and S1 subsites are relatively conserved, those at S2 and S4 subsites only share 37.5% and 20% identity, respectively (Figure 1c,d; Figure S2, Supporting Information). Accordingly, for all 32 CoV 3CL^pro^s together, the amino acids at the S1' and S1 subsites show relatively high conservation with 50% and 66.7% identity, respectively, whereas it is only 25% and 20% identity for the S2 and S4 subsites, respectively (Figure 1c,d; Figure S2, Supporting Information). In addition to the sequence identity analysis, we explored structural/conformational changes of the subsites by overlaying 3D‐structures of the 32 CoV 3CL^pro^s that were determined by X‐ray protein crystallography or predicted by Alphafold2 (Figure 1c). In line with the sequence identity data, similar conformations of the S1'/S1 subsites but diverse structures/conformations of the S2/S4 subsites are unveiled (Figure 1c; Figure S3, Supporting Information). According to the diverse conformations of S2/S4 subsites, the 32 CoV 3CL^pro^s could be grouped into three clusters (Figure 1e). The substrate binding pockets of the α‐genus CoV 3CL^pro^s share a similar conformation, and so do the β‐genus CoV 3CL^pro^s. By contrast, both the γ‐ and δ‐genus CoV 3CL^pro^s exhibit a resemble substrate binding pocket and are thus classified into a unique third cluster. Collectively, these findings provide important insight into the sequence as well as structure conservation of the substrate binding pocket of 3CL^pro^ across the *Orthocoronavirinae* subfamily, suggesting challenges of identifying suitable P2 and P4 segments to fit well into the varied S2 and S4 subsites for the development of pan‐genus 3CL^pro^ inhibitors.

### 2.2 Broad‐Spectrum Inhibitory Activity Measurement of Three Approved Inhibitors

**Figure 2:**
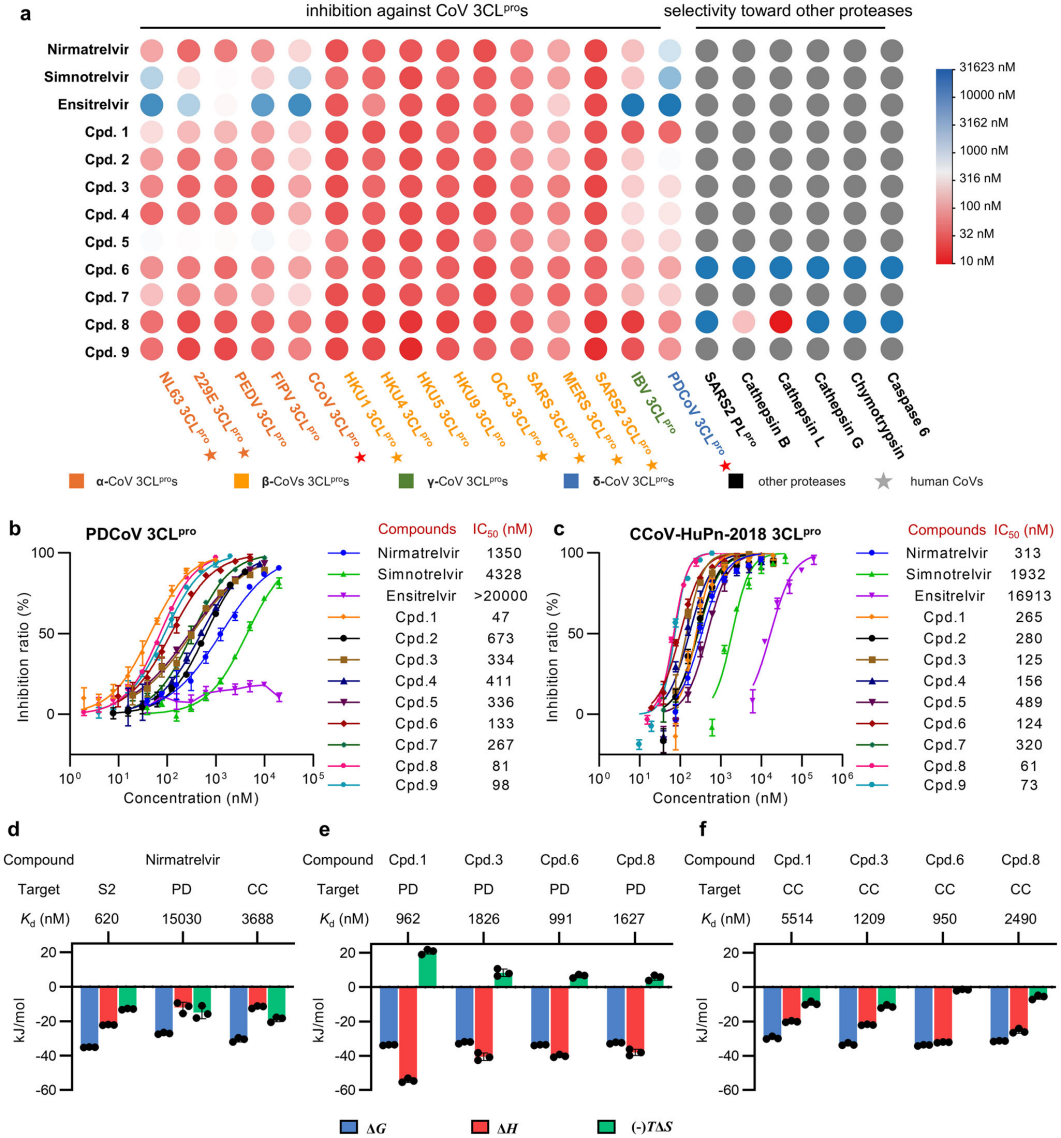
Inhibitory activities and thermodynamic profiles of compounds toward 3CL^pro^s of a variety of CoVs. a) Heatmap of IC_50_ values of nirmatrelvir, simnotrelvir, ensitrelvir, and compounds **1**–**9** against fifteen 3CL^pro^s from four genera and six other proteases, including SARS‐CoV‐2 PL^pro^, human cathepsin B, L, and G, bovine chymotrypsin, and human caspase 6. Nine human‐infecting CoVs are marked with pentagrams. b, c) Representative inhibitory profiles for nirmatrelvir (blue circle), simnotrelvir (green triangle), ensitrelvir (purple inverted triangle), **1** (orange rhombus), **2** (black circle), **3** (brown square), **4** (dark blue triangle), **5** (dark purple inverted triangle), **6** (red rhombus), **7** (dark green rhombus), **8** (pink hexagon) and **9** (cyan hexagon) against PDCoV (b) and CCoV‐HuPn‐2018 (c) 3CL^pro^s. The data are plotted as the mean ± SD. Three independent experiments were performed to determine the IC_50_ values. d) Thermodynamic profiles of nirmatrelvir binding to catalytically inactive SARS‐CoV‐2 C145G (S2), PDCoV C144G (PD), and CCoV‐HuPn‐2018 C144G (CC) 3CL^pro^ mutants, measured by isothermal titration calorimetry (ITC). e, f) Thermodynamic profiles of compounds **1**, **3**, **6,** and **8** binding to PDCoV C144G (e) and CCoV‐HuPn‐2018 C144G (f) 3CL^pro^ mutants measured by ITC. The data are plotted as the mean ± SD. Three independent experiments were performed to determine the thermodynamic profiles.

A panel of fifteen 3CL^pro^s from nine HCoVs and six other CoVs of concern were first selected to measure the broad‐spectrum inhibitory activity of three marketed 3CL^pro^ inhibitors that contain two covalent peptidomimetic inhibitors, nirmatrelvir and simnotrelvir, and a non‐covalent and non‐peptidic inhibitor, ensitrelvir (**Figure** **2**a). These fifteen CoVs encompass five α‐CoVs (HCoV‐NL63, HCoV‐229E, PEDV, FIPV, and CCoV‐HuPn‐2018), eight β‐CoVs (HCoV‐HKU1, BtCoV‐HKU4, BtCoV‐HKU5, BtCoV‐HKU9, HCoV‐OC43, SARS‐CoV, MERS‐CoV, and SARS‐CoV‐2), one γ‐CoV (IBV), and one δ‐CoV (PDCoV). We expressed and purified the fifteen recombinant 3CL^pro^s and utilized a fluorescence resonance energy transfer (FRET)‐based enzymatic assay to determine the half‐maximal inhibitory concentrations (IC_50_s) of the three compounds against each 3CL^pro^. For a direct comparison of IC_50_ values measured across 3CL^pro^ from various coronaviruses, a concentration of 100 nm was uniformly used for all 3CL^pro^s in the FRET‐based enzymatic assays.

It shows that the three inhibitors effectively inhibit eight β‐CoV 3CL^pro^s with IC_50_ values in the range of 26–267 nm (Figure 2a; **Table** **1**). By contrast, they all exhibit significantly lower inhibitory activities against 3CL^pro^s of α‐, δ‐, and γ‐CoVs, in particular 3CL^pro^s of α‐genus CCoV‐HuPn‐2018 and δ‐genus PDCoV. Furthermore, three inhibitors show different inhibitory profiles against the panel of fifteen 3CL^pro^s. Ensitrelvir demonstrates IC_50_ values of 16126, 2332, 543, 10444, and 16913 nm against 3CL^pro^s of α‐HCoV‐NL63, α‐HCoV‐229E, α‐PEDV, α‐FIPV, and α‐CCoV‐HuPn‐2018, respectively (Figure 2a; Table 1). It fails to inhibit 3CL^pro^s of γ‐IBV and δ‐PDCoV even at a concentration of 20000 nm (Figure 2a; Table 1). These IC_50_ values are substantially higher than those against β‐CoV 3CL^pro^s, reflecting a significant loss of inhibitory potency toward non‐β CoV 3CL^pro^s. Two covalent peptidomimetic inhibitors, nirmatrelvir and simnotrelvir, show broader inhibitory profiles across multiple CoV 3CL^pro^s (Figure 2a; Table 1). Nirmatrelvir is a potent inhibitor of most 3CL^pro^s and the lowest IC_50_ value is 1350 nm toward δ‐PDCoV 3CL^pro^. The inhibitory profile of simnotrelvir is similar to that of nirmatrelvir but with much lower potency toward 3CL^pro^s of five α‐CoVs as well as δ‐PDCoV. The IC_50_ values of nirmatrelvir against 3CL^pro^s of five α‐CoVs (HCoV‐NL63, HCoV‐229E, PEDV, FIPV, and CCoV‐HuPn‐2018), γ‐IBV, and δ‐PDCoV are 132, 48, 65, 101, 313, 209, and 1350 nm, respectively, while the equivalent values are 2222, 365, 587, 278, 1932, 234, and 4328 nm, respectively, for simnotrelvir (Figure 2a; Table 1). Such a difference may be attributed to the larger P2 segment in simnotrelvir in comparison to nirmatrelvir. Together, these data demonstrate for the first time that three approved 3CL^pro^ inhibitors are able to potently inhibit 3CL^pro^s of β‐CoVs but not those of the other three genera of CoVs. It is also suggested that the inhibitory profile against the panel of fifteen 3CL^pro^s is tightly associated with the binding mode as well as the scaffold of the inhibitor.

**Table 1:** IC_50_ (nM) of compounds against fifteen 3CL^pro^s.

genus	α					β								γ	δ
CoV 3CL^pro^	NL63	229E	PEDV	FIPV	CCoV	HKU1	HKU4	HKU5	HKU9	OC43	SARS	MERS	SARS2	IBV	PDCoV
Nirmatrelvir	132	48	65	101	313	31	39	34	37	30	70	133	26	209	1350
Simnotrelvir	2222	365	587	278	1932	53	46	28	46	38	70	119	26	234	4328
Ensitrelvir	16 126	2332	543	10 444	16 913	34	79	34	31	47	55	267	27	>20000	> 20000
**1**	342	190	179	131	265	31	31	28	50	38	99	141	31	38	47
**2**	120	58	74	79	280	31	44	30	36	40	88	170	32	248	673
**3**	77	43	50	36	125	29	46	33	39	30	57	68	25	262	334
**4**	47	51	50	50	156	33	39	29	33	31	55	72	30	331	411
**5**	683	592	579	727	489	70	32	29	31	67	78	132	44	239	336
**6**	92	64	48	85	124	51	36	39	38	28	54	55	42	126	133
**7**	207	86	105	160	320	30	29	33	43	28	64	47	45	179	267
**8**	53	29	35	41	61	28	22	19	24	28	38	106	21	22	81
**9**	53	26	27	43	73	26	28	16	34	44	50	85	17	33	98

It is noteworthy that the inhibition of three drugs toward 3CL^pro^s of two newly characterized HCoVs, α‐CCoV‐HuPn‐2018 and δ‐PDCoV, is worst compared to other 3CL^pro^s (Figure 2a–c; Table 1). Specifically, the measured IC_50_ values of nirmatrelvir, simnotrelvir, and ensitrelvir against δ‐PDCoV 3CL^pro^ were 1350, 4328, and >20000 nM, respectively, each of which is the lowest one among fifteen IC_50_ values (Figure 2a,b; Table 1). This raises a major concern about the lack of sufficient broad‐spectrum antiviral activity of existing pharmaceuticals, especially when they are unable to effectively block the hydrolytic activity of all nine HCoV 3CL^pro^s. To overcome this concern, it is important to develop novel inhibitors that can potently inhibit 3CL^pro^s from diverse genera of CoVs, including δ‐PDCoV and α‐CCoV‐HuPn‐2018. On the basis of its favorable inhibition profile across 3CL^pro^s from fifteen CoVs spanning four distinct genera, we selected nirmatrelvir as the starting scaffold for subsequent structure‐based optimization to gain pan‐genus CoV 3CL^pro^ inhibitors.

### 2.3 Mechanism of Action of Nirmatrelvir with PDCoV and CCoV‐HuPn‐2018 3CL^pro^s

**Figure 3:**
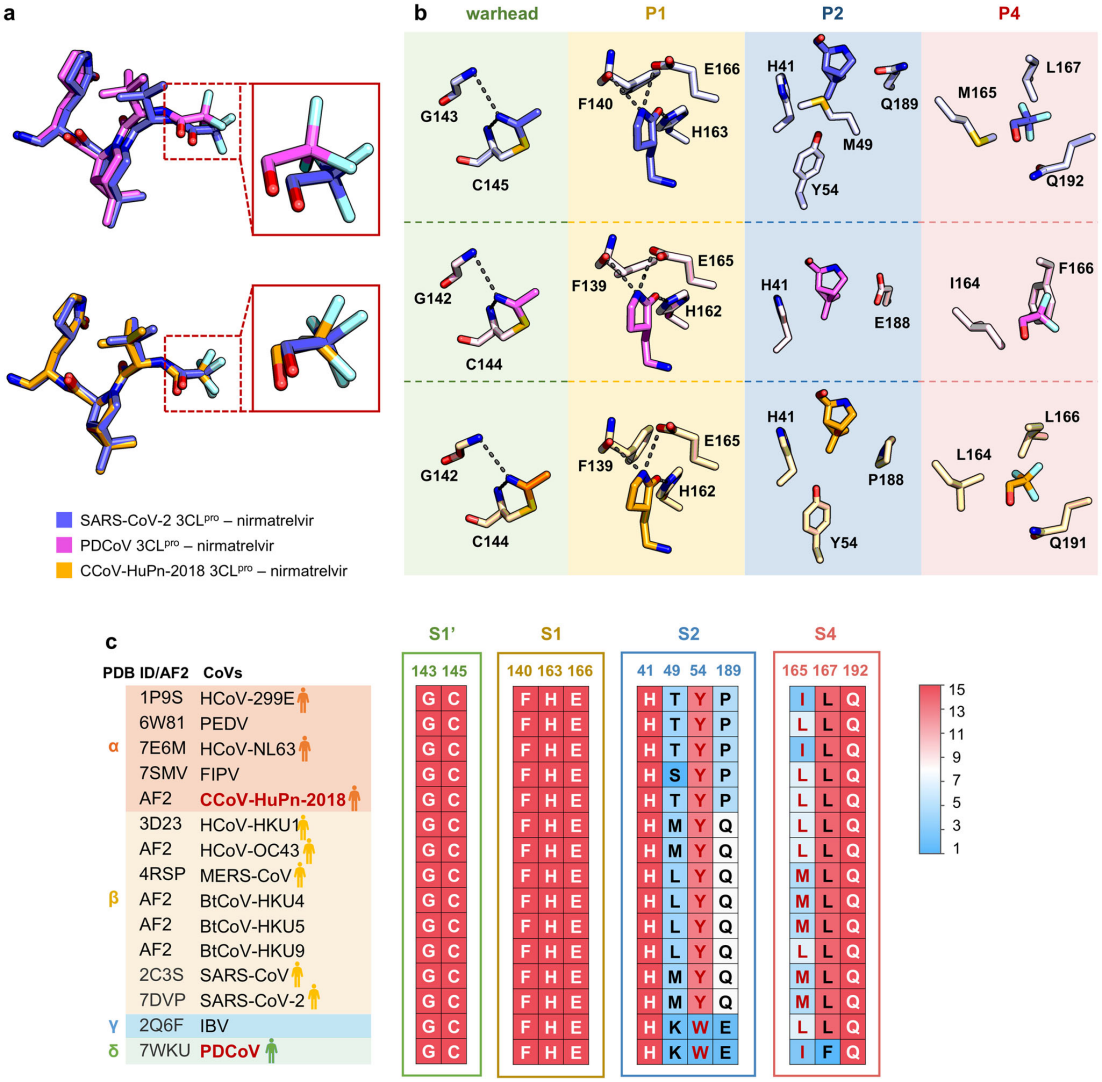
Interactions of nirmatrelvir with 3CL^pro^s from different CoVs. a) The well‐overlapped binding poses of nirmatrelvir with SARS‐CoV‐2 3CL^pro^ (violet, PDB code: 8IGY), PDCoV 3CL^pro^ (magenta, PDB code: 9 Ｘ7D) and CCoV‐HuPn‐2018 3CL^pro^ (orange, PDB code: 9 Ｘ7E) revealed by co‐crystal structures. b) Interaction pattern comparison of the warhead, P1, P2, and P4 segments of nirmatrelvir with SARS‐CoV‐2, PDCoV, and CCoV‐HuPn‐2018 3CL^pro^s, respectively. c) 3D structure‐based sequence conservation analysis of key residues at four subsites (S1ʹ, S1, S2, and S4) of SARS‐CoV‐2 3CL^pro^ interacting with nirmatrelvir in the fifteen CoV 3CL^pro^s. Heatmap colors indicate the frequency. HCoVs are marked with a man icon. 3D structures of 3CL^pro^s predicted by AlphaFold2 are marked as “AF2”, otherwise PDB codes are provided. The residue numberings are for SARS‐CoV‐2 3CL^pro^.

To understand the molecular mechanism of action responsible for reduced inhibition of nirmatrelvir against PDCoV and CCoV‐HuPn‐2018 3CL^pro^s compared to SARS‐CoV‐2 3CL^pro^, crystal structures of PDCoV and CCoV‐HuPn‐2018 3CL^pro^s in complex with nirmatrelvir were determined at 2.3 and 2.4 Å resolution, respectively (Table S1, Supporting Information). In addition, the dissociation constant (*K*_d_) measured by isothermal titration calorimetry (ITC) was determined using 3CL^pro^ mutants in which the catalytic cysteine was substituted with glycine to evaluate the non‐covalent binding affinity of nirmatrelvir. Aligning with the trend in IC_50_ values, the *K*_d_ values of nirmatrelvir binding to PDCoV and CCoV‐HuPn‐2018 3CL^pro^s were determined to be 15030 and 3688 nm, respectively (Figure 2d; Table S2, Supporting Information), demonstrating approximately a 24‐ and 6‐fold decrease compared to its binding affinity to SARS‐CoV‐2 3CL^pro^ (*K*_d_ = 620 nm). By contrast, the co‐crystal structures reveal that the overall binding modes of nirmatrelvir to PDCoV and CCoV‐HuPn‐2018 3CL^pro^s closely resemble its binding mode to SARS‐CoV‐2 3CL^pro^ (**Figure** **3**a). However, subtle conformational changes have been observed for the P4 segment of nirmatrelvir bound in PDCoV or CCoV‐HuPn‐2018 3CL^pro^s (Figure 3a). A comprehensive analysis of interactions between nirmatrelvir and these three 3CL^pro^s was thus conducted to uncover the molecular implications for the difference in the binding affinity as well as the configurational changes.

The interactions of nirmatrelvir with PDCoV and CCoV‐HuPn‐2018 3CL^pro^s mainly resemble those observed in its complex with SARS‐CoV‐2 3CL^pro^: (1) the nitrile warhead forms a covalent thioimidate ester conjugate with the catalytic cysteine and is stabilized by hydrogen bonds (H ‐bonds) with residues at the oxyanion hole of three 3CL^pro^s (Figure 3b); (2) the P1‐5‐membered lactam of nirmatrelvir fits well into the S1 subsite of the three 3CL^pro^s, making favorable H‐bonds with H163/E166/F140 of SARS‐CoV‐2 3CL^pro^ and H162/E165/F139 of PDCoV and CCoV‐HuPn‐2018 3CL^pro^s, respectively (Figure 3b); (3) the backbone amide groups of nirmatrelvir form three H ‐bonds with the main chains of E165/H163 of PDCoV 3CL^pro^ (H‐bond lengths: 2.9, 3.0, and 3.1 Å, Figure S4, Supporting Information) and CCoV‐HuPn‐2018 3CL^pro^ (H‐bond lengths: 3.0, 2.9, and 3.0 Å, Figure S4, Supporting Information), similar to those H ‐bonds with E166/H164 of SARS‐CoV‐2 3CL^pro^ (H‐bond lengths: 2.9, 2.9, and 2.9 Å, Figure S4, Supporting Information); and (4) the P3 segment of nirmatrelvir always orients toward the solvent region of three 3CL^pro^s.

The primary distinction lies in the interactions between nirmatrelvir and S2/S4 subsites of three 3CL^pro^s. The P2/P4 segments of nirmatrelvir establish a large set of favorable hydrophobic interactions with the surrounding residues at the S2 (H41, Y54, M49, and Q189) as well as the S4 (M165, L167, and Q192) subsites of SARS‐CoV‐2 3CL^pro^ (Figure 3b). In case of CCoV‐HuPn‐2018 3CL^pro^, it has relatively weaker, less‐oriented hydrophobic interactions with residues at the S2 (H41, Y54, and P188) and the S4 (L164, L166, and Q191) subsites (Figure 3b). In contrast, nirmatrelvir engages with only two residues at the S2 (H41 and E188) and the S4 (I164 and F166) subsites of PDCoV 3CL^pro^, resulting in the least interactions formed among the three complexes (Figure 3b). In line with the structural information, thermodynamic profiles revealed by ITC measurements indicate that the decreased non‐covalent binding affinity of nirmatrelvir to PDCoV and CCoV‐HuPn‐2018 3CL^pro^s is attributed to a significantly lower enthalpic gain compared to its binding enthalpy to SARS‐CoV‐2 3CL^pro^ (Figure 2d). Therefore, biophysical, structural, and biochemical data together demonstrate that the favorable interaction pattern of the P2/P4 segments with the S2/S4 subsites results in an enthalpy‐driven binding signature of nirmatrelvir to SARS‐CoV‐2 3CL^pro^. Otherwise, it leads to an entropy‐driven binding signature of nirmatrelvir to the other two 3CL^pro^s (Figure 2d). Moreover, sequence alignment analysis was performed on the fifteen 3CL^pro^s to evaluate the conservation of key residues involved in the interactions of nirmatrelvir with SARS‐CoV‐2 3CL^pro^. It is indicated that residues engaged in interactions with nirmatrelvir at the S1' and S1 subsites remain conserved across all fifteen CoVs, while those at the S2 and S4 subsites show significant variation (Figure 3c). This is well consistent with the above‐mentioned conserved features of the substrate binding pocket of 59 3CL^pro^s. Accordingly, these observations suggest interactions with the S2/S4 subsites are crucial for the incorporation of broad‐spectrum inhibitors into variable binding pockets of 3CL^pro^s, and also raise a key question as to how the inhibitors achieve favorable interactions with the S2/S4 subsites of various 3CL^pro^s by optimizing the P2/P4 segments.

**Figure 4:**
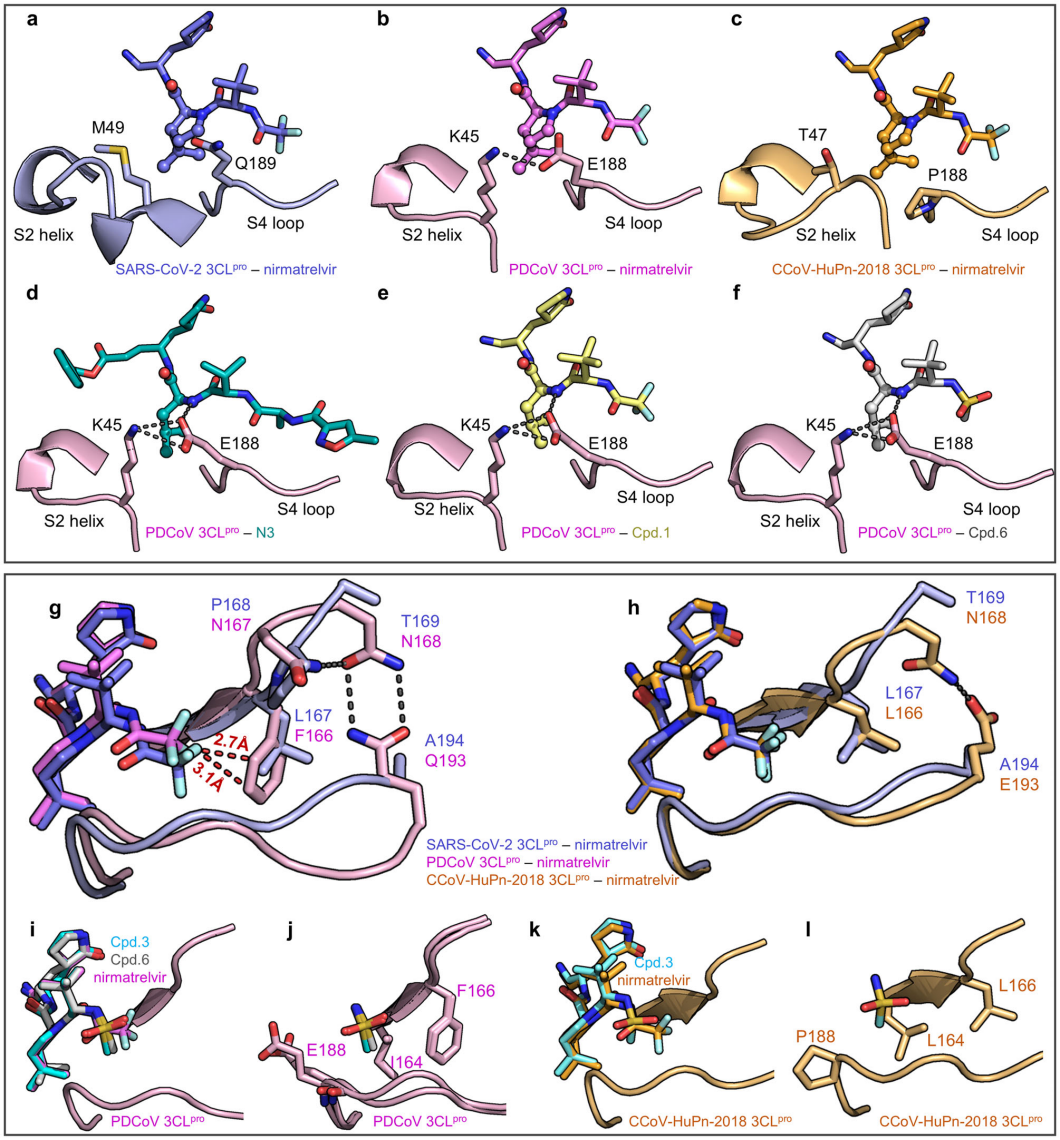
Key interactions of P2/P4 segments of inhibitors with SARS‐CoV‐2, PDCoV, and CCoV‐HuPn‐2018 3CL^pro^s revealed by co‐crystal structures. a–c) Key interactions of nirmatrelvir P2 segment with the S2 subsite of SARS‐CoV‐2 3CL^pro^ (violet cartoons, PDB code: 8IGY), PDCoV 3CL^pro^ (pink cartoons, PDB code: 9 X7D) and CCoV‐HuPn‐2018 3CL^pro^ (light brown cartoons, PDB code: 9X7E). d–f) Interactions of N3 (d, PDB code: 7WKU), **1** (e, PDB code: 9X7F), and **6** (f, PDB code: 9X7H) P2 segments with PDCoV 3CL^pro^. Compounds N3, **1**, and **6** are shown as deep teal, yellow, and white sticks, respectively. PDCoV 3CL^pro^ is shown as pink cartoons. g, h) The overlap of nirmatrelvir P4 segment interacting with SARS‐CoV‐2 3CL^pro^ (violet cartoons) and PDCoV 3CL^pro^ (pink cartoons) (g) or CCoV‐HuPn‐2018 3CL^pro^ (light brown cartoons) (h) based on the co‐crystal structures. i) Comparison of interaction patterns of nirmatrelvir (PDB code: 9X7D), **3** (PDB code: 9X7G), and **6** (PDB code: 9X7H) P4 segments with PDCoV 3CL^pro^ by superimposition of their co‐crystal structures. Nirmatrelvir, **3**, and **6** are represented by magenta, cyan, and white sticks, respectively. j) Interactions of the P4 segments of **3** (PDB code: 9X7G) and **6** (PDB code: 9X7H) with PDCoV 3CL^pro^. PDCoV 3CL^pro^ is shown as pink cartoons. k) Comparison of the interaction patterns of nirmatrelvir (PDB code: 9X7E) and **3** (PDB code: 9X7I) P4 segments with CCoV‐HuPn‐2018 3CL^pro^ by superimposition of their co‐crystal structures. Nirmatrelvir and **3** are represented by orange and cyan sticks, respectively. l) Interactions between the P4 segment of **3** and CCoV‐HuPn‐2018 3CL^pro^ (PDB code: 9X7I) that is shown as light brown cartoons.

A detailed analysis of nirmatrelvir P2 segment interacting with the three 3CL^pro^s reveals a distinctive K45‐E188 salt bridge at the S2 subsite of PDCoV 3CL^pro^, which is absent in SARS‐CoV‐2 and CCoV‐HuPn‐2018 3CL^pro^s due to equivalent residues of M and Q in the former and T and P in the latter (**Figure** **4**a–c). This salt bridge stabilizes the S2 helix and S4 loop, resulting in a more rigid and smaller S2 subsite compared to those of SARS‐CoV‐2 and CCoV‐HuPn‐2018 3CL^pro^s. Additionally, upon examining a previously reported crystal structure of PDCoV 3CL^pro^ in complex with N3, identified as a moderate inhibitor with an IC_50_ value of 683 nm in our assay, a strong H‐bond is observed between E188 and the backbone amide group of N3 (Figure 4d). However, such a H‐bond is absent in the complex of nirmatrelvir binding with PDCoV 3CL^pro^ (Figure 4b), as the backbone amide group at the equivalent position of nirmatrelvir is cyclized. Moreover, an observed conformational change occurs around E188 of PDCoV 3CL^pro^ when bound to nirmatrelvir as compared to N3, leading to the weakened salt bridge between E188 and K45 (Figure 4b,d). Therefore, both the sequence variation of 3CL^pro^s and the chemical structure modification of inhibitors contribute to the change of inhibitor‐S2 interaction pattern.

An exhaustive comparative analysis of interactions of nirmatrelvir P4 segment with 3CL^pro^s was subsequently conducted. The crystal structure of SARS‐CoV‐2 3CL^pro^ bound with nirmatrelvir was superimposed onto that of PDCoV or CCoV‐HuPn‐2018 3CL^pro^ (Figure 4g,h). The P4 segment of nirmatrelvir establishes strong hydrophobic interactions with L167 at the S4 subsite of SARS‐CoV‐2 3CL^pro^. However, this residue is substituted with phenylalanine (F166) in PDCoV 3CL^pro^, resulting in a steric hindrance to the binding of the P4 segment. The hindrance is further augmented by a bidentate H‐bond network involving residues N167, N168, and Q193, which is completely absent in SARS‐CoV‐2 3CL^pro^ (Figure 4g). Consequently, the large side‐chain of F166 results in a conformation shift of the P4 segment of nirmatrelvir when bound to PDCoV 3CL^pro^ compared to that in the complex with SARS‐CoV‐2 3CL^pro^ (Figures 3a and 4g). In CCoV‐HuPn‐2018 3CL^pro^, it is also a leucine (L166) at the position equivalent to L167 of SARS‐CoV‐2 3CL^pro^. The side‐chain conformation of L166 is governed by the H‐bonding of N168 to E193, leading to a more rigid S4 subsite in CCoV‐HuPn‐2018 3CL^pro^ compared to SARS‐CoV‐2 3CL^pro^ (Figure 4h). Therefore, it is rational to introduce a group more flexible or smaller than the trifluoroacetyl group at the P4 position of nirmatrelvir so as to enhance the inhibitor's potency against PDCoV and CCoV‐HuPn‐2018 3CL^pro^s.

### 2.4 Structure‐Based Design of pan‐3CL^pro^ inhibitors

**Figure 5:**
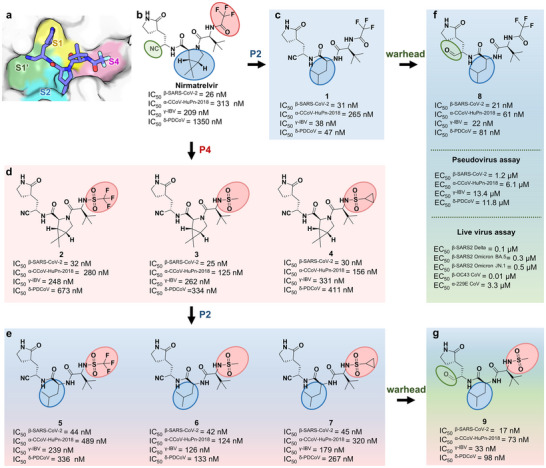
Structure‐guided design, chemical structures, and inhibitory activities of new compounds at the molecular and cellular levels. a) Molecular surface representation of nirmatrelvir interacting with the S1ʹ‐S4 subsites of SARS‐CoV‐2 3CL^pro^ (PDB code: 8IGY). Nirmatrelvir is shown in violet sticks, while the S1ʹ, S1, S2, and S4 subsites are colored light green, light yellow, light cyan, and light pink, respectively. b) The chemical structure of nirmatrelvir and its IC_50_ values toward α‐CCoV‐HuPn‐2018, β‐SARS‐CoV‐2, γ‐IBV, and δ‐PDCoV 3CL^pro^s. c–e) Chemical structures and inhibitory activities of new compounds that were designed to favorably interact with the S2 (c), S4 (d), and S2/S4 (e) subsites of α‐CCoV‐HuPn‐2018, β‐SARS‐CoV‐2, γ‐IBV, and δ‐PDCoV 3CL^pro^s. f, g) Chemical structures and inhibitory activities at the molecular and cellular levels of new compounds further modified with the covalent warhead. Three independent experiments were performed to determine the IC_50_ values.

The comprehensive analysis of interactions of nirmatrelvir with SARS‐CoV‐2, PDCoV, and CCoV‐HuPn‐2018 3CL^pro^s based on a series of co‐crystal structures, coupled with thermodynamic profile studies and sequence conservation analysis, highlights the potential utility of modifying nirmatrelvir's P2 and P4 segments to enhance the broad‐spectrum inhibitory activity. Based on our findings that nirmatrelvir, simnotrelvir, and previously reported cyclized P2 analogues (**1a**–**1c**)^[^ ^8^ ^]^ are potent against SARS‐CoV‐2 3CL^pro^ but exhibit poor inhibition of PDCoV 3CL^pro^ (IC_50_ > 1 µm), we decided to avoid cyclic P2 segments. This decision was further guided by structural insight that a free (non‐cyclized) P2 amine enables to form a hydrogen bond with E188 in PDCoV 3CL^pro^, a key interaction disrupted upon cyclization. In addition, we systematically analyzed the substrate preferences of diverse CoV 3CL^pro^s. Although some variation exists, leucine is consistently favored at the P2 position across CoVs (Figure S5, Supporting Information). Accordingly, we incorporated an isobutyl group—structurally identical to the leucine side chain and the P2 substituent in N3—and synthesized compound **1** (**Figure** **5**a–c). Encouragingly, compound **1** exhibits an IC_50_ value of 47 nm against PDCoV 3CL^pro^ and 265 nm against CCoV‐HuPn‐2018 3CL^pro^ (Figures 2b,c and 5c; Table 1), achieving a 29‐fold improvement in potency against PDCoV 3CL^pro^ compared to nirmatrelvir. The inhibitory activity against CCoV‐HuPn‐2018 3CL^pro^ is also slightly higher for compound **1** than that of nirmatrelvir (IC_50_: 265 vs 313 nm, Table 1). Consistently, the ITC measured *K*_d_ value of compound **1** binding to PDCoV 3CL^pro^ enhances ≈16‐fold compared to nirmatrelvir (*K*_d_: 962 vs 15030 nm, Figure 2d,e), and two compounds have similar binding affinity to CCoV‐HuPn‐2018 3CL^pro^ (*K*_d_: 5514 vs 3688 nm, Figure 2d,f). The determined crystal structure of PDCoV 3CL^pro^ bound with compound **1** reveals a new H‐bond formed between E188 and the backbone amide group of **1** (Figure 4e), partly accounting for the improved IC_50_ and *K*_d_ values of **1** compared to nirmatrelvir. Additionally, the formation of this H‐bond is accompanied by an exceptionally large enthalpy gain, indicating more specific inhibitor‐protease interactions formed and a more thermodynamically favorable binding for compound **1** over nirmatrelvir to PDCoV 3CL^pro^ (Figure 2d,e). Moreover, IC_50_s of compound **1** against the other thirteen CoV 3CL^pro^s were determined to assess its broad‐spectrum activity (Figure 2a; Table 1). Impressively, compound **1** is also 6‐fold more potent than nirmatrelvir against 3CL^pro^ of γ‐IBV (IC_50_: 38 vs 209 nm, Figure 2a; Table 1), another 3CL^pro^ that nirmatrelvir struggled to have an effective inhibition. Compound **1** also retains the potent inhibitory activity against 3CL^pro^s of α‐FIPV and eight β‐CoVs (Figure 2a; Table 1). Nevertheless, toward 3CL^pro^s of α‐HCoV‐NL63, α‐HCoV‐229E, and α‐PEDV, compound **1** demonstrates a 3‐, 4‐, and 3‐fold decrease in IC_50_ values compared to nirmatrelvir (Figure 2a; Table 1), respectively. Overall, structure‐guided replacement of the P2‐dimethyl cyclopropyl‐proline of nirmatrelvir by the P2‐isopentane segment allows compound **1** to improve, in general, the broad‐spectrum inhibitory activity against fifteen 3CL^pro^s, in particular to significantly increase the potency against 3CL^pro^s of two newly characterized HCoVs (PDCoV and CCoV‐HuPn‐2018).

Subsequently, we focused on optimization of the P4 segment. A thorough examination of S4 subsites indicated that a smaller and more flexible P4 moiety than the P4‐trifluoroacetyl group of nirmatrelvir would be better tolerated, particularly in PDCoV and CCoV‐HuPn‐2018 3CL^pro^s. Therefore, to replace the relatively rigid and bulky amide‐based P4 group of nirmatrelvir, we employed a sulfonyl linker, which offers greater conformational flexibility, a more compact profile, and synthetic tractability for efficient analog generation. Accordingly, compounds **2**–**4** derived from nirmatrelvir with diverse P4 segments, namely P4‐trifluoromethylsulfonyl (**2**), P4‐methylsulfonyl (**3**), and P4‐cyclopropanesulfonyl (**4**) groups, were designed, synthesized, and subjected to biochemical evaluation (Figure 5d). The measured IC_50_ values of compounds **2**–**4** are 673, 334, and 441 nm against PDCoV 3CL^pro^, respectively (Figures 2b and 5d; Table 1), improving the potency compared to nirmatrelvir (IC_50_: 1350 nm). Similarly, their IC_50_ values toward CCoV‐HuPn‐2018 3CL^pro^ are 280, 125, and 156 nm (Figures 2c and 5d; Table 1), respectively, indicating comparable or enhanced inhibitory activity relative to nirmatrelvir (IC_50_: 313 nm). Additionally, three compounds exhibit comparable inhibition against the other thirteen 3CL^pro^s compared to nirmatrelvir (Figure 2a; Table 1). Among them, compound **3**, bearing the smallest P4 segment, shows the most potent inhibitory activity against PDCoV and CCoV‐HuPn‐2018 3CL^pro^s (Figures 2b,c and 5d; Table 1), with approximately a 4‐ and 3‐fold improvement in potency relative to nirmatrelvir. In parallel, the *K*_d_ values of compound **3** binding to PDCoV and CCoV‐HuPn‐2018 3CL^pro^s also demonstrate significant enhancements with an 8‐ and 3‐fold over those of nirmatrelvir, respectively (Figure 2d–f). The determined crystal structures of PDCoV and CCoV‐HuPn‐2018 3CL^pro^s in complex with **3** reveal a resemble binding mode of **3** and nirmatrelvir to PDCoV or CCoV‐HuPn‐2018 3CL^pro^, albeit with variations in their interactions with the S4 subsites (Figure 4i–l). As expected, the smaller P4 segment of compound **3** fits better into the rigid S4 subsite of both PDCoV and CCoV‐HuPn‐2018 3CL^pro^s. It engages in optimal hydrophobic interactions with I164/F166/E188 and L164/L166/P188 at the S4 subsite of PDCoV (Figure 4j) and CCoV‐HuPn‐2018 3CL^pro^s (Figure 4l), respectively. Aligned with the structural insights, the thermodynamic binding of compound **3** to these two 3CL^pro^s is mainly driven by enthalpy, in contrast to the entropy‐driven binding signature of nirmatrelvir (Figure 2d–f). Therefore, the replacement of the P4 segment of nirmatrelvir with a smaller one also leads to a pronounced potency gain of new compounds against PDCoV and CCoV‐HuPn‐2018 3CL^pro^s.

Given the encouraging outcomes from the P4 segment modifications of nirmatrelvir, we added these new P4 segments to compound **1** for potentially additive or synergistic effects on the broad‐spectrum potency. The resulting compounds **5**–**7**, bearing the identical P4 segment of compounds **2**–**4** (Figure 5e), display IC_50_ values of 336, 133, and 267 nm respectively, toward PDCoV 3CL^pro^, and 489, 124, and 320 nm, respectively toward CCoV‐HuPn‐2018 3CL^pro^ (Figures 2b,c and 5e; Table 1). Among them, compound **6**, containing the P4 segment identical to that of compound **3**, exhibits the most potent inhibitory activity against PDCoV and CCoV‐HuPn‐2018 3CL^pro^s. The *K*_d_ values of compound **6** binding to PDCoV and CCoV‐HuPn‐2018 3CL^pro^s also show 15‐ and 4‐fold enhancements, respectively, relative to nirmatrelvir. The crystal structure of PDCoV 3CL^pro^ bound with **6** reveals that the backbone amide group of P2 segment forms a H‐─bond with E188 of the S2 subsite (Figure 4f) while the P4‐methylsulfonyl moiety fits well into the S4 subsite (Figure 4i,j). Additionally, the binding modes of compound **6** with the 3CL^pro^s from α‐CCoV‐HuPn‐2018, β‐SARS‐CoV‐2, γ‐IBV, and δ‐PDCoV are similar, which is revealed by the determined co‐crystal structures (Figure S6a, Supporting Information). Thermodynamic profiles measured by ITC also reveal a more favorable enthalpy‐driven binding to PDCoV and CCoV‐HuPn‐2018 3CL^pro^s for **6** compared to nirmatrelvir (Figure 2d–f). Remarkably, compound **6** exhibits enhanced inhibitory potency against 3CL^pro^s of α‐CCoV‐HuPn‐2018, γ‐IBV, and δ‐PDCoV, whereas nirmatrelvir struggles to inhibit them effectively (Figure 2a–c; Table 1). Overall, compound **6** demonstrates potent inhibitory activity against the 3CL^pro^s of 15 CoVs, with IC_50_ values ranging from 28 to 133 nM (Figure 2a; Table 1), providing a congener of nirmatrelvir but with superior broad‐spectrum inhibition against various 3CL^pro^s from four genera of CoVs.

**Figure 6:**
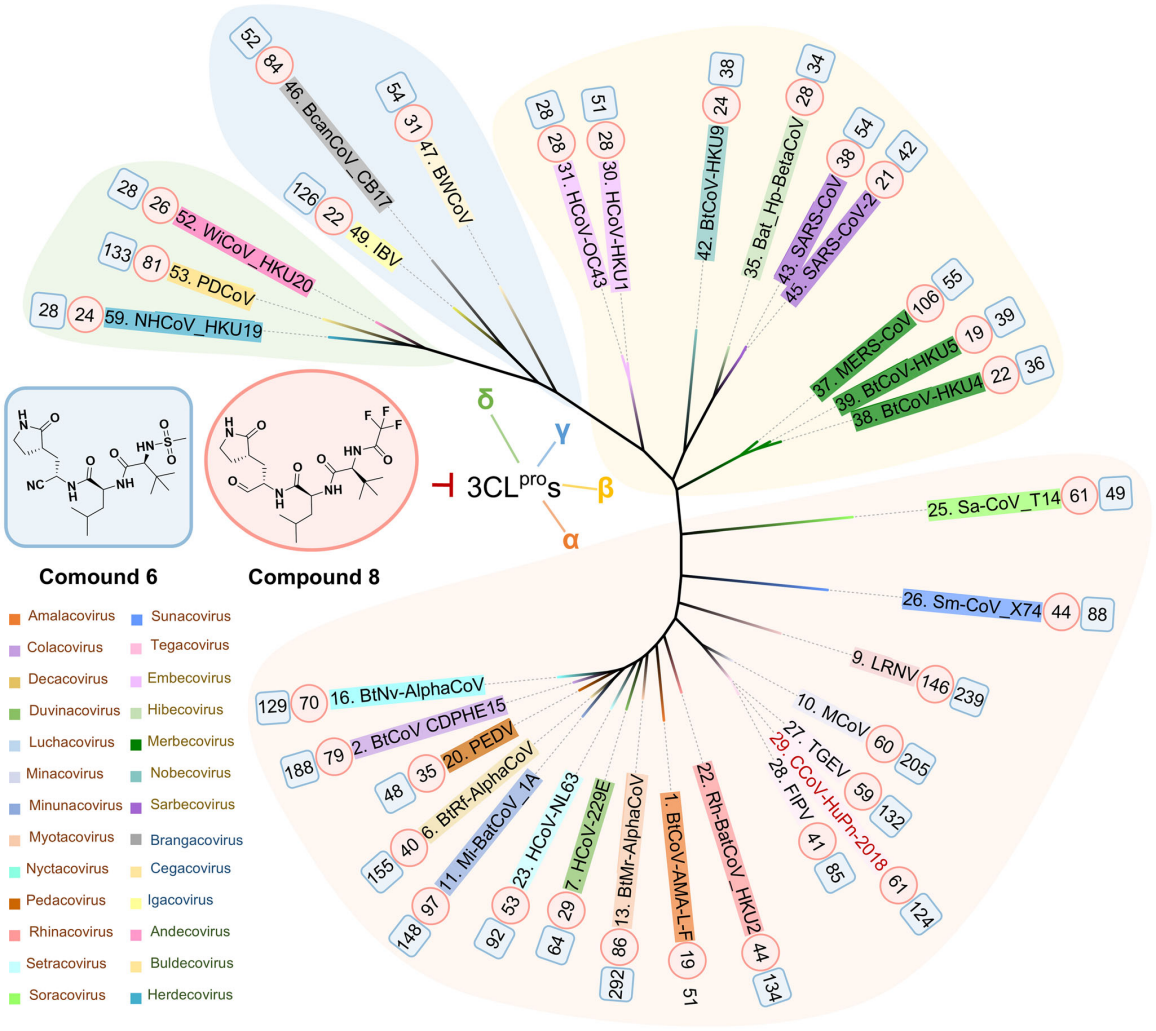
Broad‐spectrum inhibition of compounds **6** and **8** against 32 3CL^pro^s covering 26 known subgenera. The unrooted phylogenetic tree of 32 3CL^pro^s was conducted based on sequences. CoVs from various subgenera are indicated by unique colors. The numerical values within the light blue rounded rectangles and pink ovals are the IC_50_ values (nM) of compounds **6** and **8**, respectively, against the 3CL^pro^s of the relevant CoVs. Experiments were performed in triplicate to determine the IC_50_ values.

Encouraged by the remarkable inhibitory potency of compound **6** against the 15 CoV 3CL^pro^s, we expanded the assessment to include 3CL^pro^s of 32 CoVs in total, covering 26 subgenera known (Figure 1b). It is surprisingly revealed that compound **6** maintains strong inhibitory activity across this broader spectrum, with IC_50_ values spanning from 28 to 292 nm (Figure 6), revealing that compound **6** is an ultra‐broad‐spectrum 3CL^pro^ inhibitor. In addition, we examined the selectivity of compound **6** by testing their inhibitory activities against SARS‐CoV‐2 PL^pro^ and several ｍammalian proteases including cathepsins B/L/G, chymotrypsin, and caspase 6. At a concentration of 20 µm, compound **6** shows no significant inhibition on these proteases (Figure 2a), indicating a high on‐target selectivity profile.

Subsequently, we utilized the ODD‐luciferase pseudovirus model^[^ ^9^ ^]^ to assess the inhibition of the compounds against α‐CCoV‐HuPn‐2018, β‐SARS‐CoV‐2, γ‐IBV, and δ‐PDCoV 3CL^pro^s at concentrations that have no cytotoxicity in HEK293T cells (Figure S7, Supporting Information). In this assay, the ODD domain is linked to luciferase via a 3CL^pro^ cleavage sequence. If 3CL^pro^ cleaves the sequence, luciferase is not degraded. Accordingly, the luciferase intensity indicates the degree of 3CL^pro^ inhibition. Using this assay, we first tested the inhibition of nirmatrelvir against 3CL^pro^s from the four coronaviruses in HEK293T cells. Nirmatrelvir effectively inhibits β‐SARS‐CoV‐2 3CL^pro^ at the cellular level (EC_50_ = 6.2 µm) but shows weaker potency against α‐CCoV‐HuPn‐2018 3CL^pro^ (EC_50_ = 44.7 µm) and no significant inhibition of γ‐IBV and δ‐PDCoV 3CL^pro^s at 200 µm (Figure S8, Supporting Information). Instead, compound **1**, which also shows superior broad‐spectrum activity compared to nirmatrelvir (Table 1), inhibits γ‐IBV and δ‐PDCoV 3CL^pro^s with a ratio of 56.1% and 62.4% at 200 µm, respectively. Unfortunately, the cellular efficacy of compound **6** under the same conditions was hampered by its poor membrane permeability, which is about one‐quarter of nirmatrelvir determined by the cell membrane permeability experiment (Figure S8, Supporting Information).

The poor membrane permeability of compounds **1** and **6,** with cLogP values of 0.62 and −0.34 respectively, led to unsatisfied antiviral activity in pseudovirus assays. To this end, we replaced the cyano covalent warhead with an aldehyde covalent warhead of similar size and properties, resulting in the development of new compounds **8** and **9** (Figure 5f,g), which exhibit improved cLogP of 0.92 and −0.04, respectively. We first tested the inhibitory activity of compounds **8** and **9** against the 3CL^pro^s of α‐CCoV‐HuPn‐2018, β‐SARS‐CoV‐2, γ‐IBV, and δ‐PDCoV at the molecular level, finding that both compounds show IC_50_ values below 100 nm (Figure 5f,g). Similar to compound **6**, the co‐crystal structures revealed that the binding modes of compound **8** with the 3CL^pro^s of four different genera (α‐CCoV‐HuPn‐2018, β‐SARS‐CoV‐2, γ‐IBV, and δ‐PDCoV) are largely conserved (Figure S6b, Supporting Information). Next, we evaluated the cellular antiviral activity of the two compounds using the ODD‐luciferase pseudovirus model. Notably, compound **8** shows significantly enhanced EC_50_ values in this cellular assay (Figure 5f; Figure S8, Supporting Information), with EC_50_ values of 1.2 µm (β‐SARS‐CoV‐2), 6.1 µm (α‐CCoV‐HuPn‐2018), 13.4 µm (γ‐IBV), and 11.8 µm (δ‐PDCoV). Compared to nirmatrelvir, compound **8** exhibits significantly enhanced inhibition of α‐CCoV‐HuPn‐2018, γ‐IBV, and δ‐PDCoV in the pseudovirus assay, with improvements of ≈7‐fold, >15‐fold, and >17‐fold, respectively (Figure S8, Supporting Information). Furthermore, compound **8** was tested against 32 3CL^pro^s covering 26 coronavirus subgenera, demonstrating IC_50_ values ranging from 19 to 146 nm (Figure 6). Compared to nirmatrelvir, compound **8** possesses ≈17‐ and 18‐fold enhanced inhibition against 3CL^pro^s of δ‐PDCoV and δ‐Sp‐CoV_HKU17 (*Buldecovirus* subgenus), ≈10‐fold stronger toward 3CL^pro^ of γ‐IBV (*Igacovirus* subgenus), and 7‐fold enhancement against 3CL^pro^ of α‐BtMr‐AlphaCoV (*Myotacovirus* subgenus) (Table S3, Supporting Information). Additionally, it also shows ≈5‐fold improved inhibition against 3CL^pro^s from α‐BtRf‐AlphaCoV (*Decacovirus* subgenus), α‐Rh‐BatCoV_HKU2 (*Rhinacovirus* subgenus), α‐Sm‐CoV_X74 (*Sunacovirus* subgenus), and both α‐TGEV and α‐CCoV‐HuPn‐2018 (*Tegacovirus* subgenus) (Table S3, Supporting Information). The stronger inhibitory activity exhibited against PDCoV and CCoV‐HuPn‐2018 3CL^pro^ is also consistent with the ITC results. Moreover, it demonstrates high selectivity over a panel of host and viral proteases, including cathepsin G, chymotrypsin, caspase 6, and SARS‐CoV‐2 PL^pro^, with IC_50_ values all exceeding 20 µm (Table S4, Supporting Information). However, its selectivity against cathepsins B and L is relatively limited, with IC_50_ values of 208 and 12 nm, respectively, similar to the profile observed for nirmatrelvir.

To further evaluate the therapeutic potential of compound **8**, we assessed its activity against drug‐resistant mutants and diverse coronavirus strains. Notably, compound **8** strongly inhibits nirmatrelvir‐resistant 3CL^pro^ mutants identified from passage experiments and naturally occurring viruses,^[^ ^10^, ^11^, ^12^, ^13^, ^14^, ^15^ ^]^ with a particularly notable 55‐fold improvement in IC_50_ against the E166V mutant compared to nirmatrelvir (Table S5, Supporting Information). Finally, we tested the antiviral efficacy of compound **8** against authentic virus strains, including the β‐SARS‐CoV‐2 Delta variant, Omicron BA.5, Omicron JN.1, as well as β‐OC43 CoV and α‐229E CoV. The results show that compound **8** effectively inhibits these viruses in cells, with EC_50_ values of 0.1, 0.3, 0.5, 0.01, and 3.3 µm, respectively (Figure 5f). These data confirm that compound **8** possesses the broad‐spectrum antiviral activity at both the molecular and cellular levels.

In the end, to demonstrate the in vivo efficacy of the ultra‐broad‐spectrum 3CL^pro^ inhibitor, as a proof‐of‐concept, we synthesized compound **10**, a phosphate prodrug of compound **8** which contains the aldehyde covalent warhead (Figure S9a,b, Supporting Information). A well‐characterized HCoV‐OC43 infection model using 5‐day‐old suckling Balb/c mice was utilized since the emerging zoonotic coronaviruses, such as PDCoV and CCoV‐HuPn‐2018 are difficult to access for in vivo studies, and compound **8** shows the most potent cellular antiviral activity against β‐OC43 CoV compared to other authentic virus strains (Figure 5f). The mice were intranasally challenged with HCoV‐OC43 and then orally treated once daily with compound **10** in combination with ritonavir (Figure S9c, Supporting Information). Nirmatrelvir, co‐administered with ritonavir, was used as a positive control. At 5 days post‐infection, viral RNA levels in the vehicle‐treated group reached ≈7.5 log copies per g in the brain. Treatment with compound **10** led to a significant, dose‐dependent reduction in viral RNA levels in the brain, with the 250 and 500 mpk doses reducing viral loads by 3.6 and 4.2 log units, respectively. Consistent with the reductions observed in the brain, viral RNA levels in the spinal cord and lung tissues also showed a decreasing trend; however, the changes were not statistically significant, likely because these tissues are not the primary sites of HCoV‐OC43 replication (Figure S9d, Supporting Information). These findings demonstrate that compound **10** is effective in vivo and holds promise as a broad‐spectrum anti‐coronavirus candidate.

## 3 Discussion

Despite considerable advances in developing vaccines, monoclonal antibodies, and small molecule drugs for the treatment of COVID‐19, there remains an unmet need for broad‐spectrum anti‐coronavirus agents that are effective against all CoV strains, including those in animal reservoirs, to prevent current and future outbreaks. 3CL^pro^ has been considered as a promising target for pan‐CoV drug development due to its proven druggability and highly conserved structure.^[^ ^3^, ^16^, ^17^, ^18^, ^19^ ^]^ However, comprehensive studies on the substrate binding pocket of 3CL^pro^ across all CoV species are lacking. In this study, an in‐depth analysis of the substrate binding pockets of 3CL^pro^s across the entire spectrum of known coronavirus species, i.e., 59 CoV 3CL^pro^s across all 26 subgenera of four genera, was carried out for the first time. A wealth of sequence and structural data allows us to reveal the significant conservation of S1'/S1 subsites alongside the notable variability of S2/S4 subsites in addition to the extremely conserved overall structure of 3CL^pro^s. The variability of the S2 and S4 subsites leads to a categorization of 3CL^pro^s into three distinct groups: class I (α‐CoV 3CL^pro^s), class II (β‐CoV 3CL^pro^s), and class III (γ‐ and δ‐CoV 3CL^pro^s). Consistent with this, the marketed inhibitors exhibit similar inhibitory potency to one class of 3CL^pro^s but not the other classes, while modifications of compounds to identify pan‐3CL^pro^ inhibitors cause either enhanced or reduced inhibitory activity against 3CL^pro^s of the same class. Nirmatrelvir, simnotrelvir, and ensitrelvir potently inhibit the enzymatic activity of class II 3CL^pro^s but display weak activity against the 3CL^pro^s from other classes. Optimization of the P2 segment of inhibitors, represented by compound **1** derived from nirmatrelvir, greatly improves the inhibitory activities against class III 3CL^pro^s, the ones that nirmatrelvir fails to inhibit. P4 modification also contributes to improvement in broad‐spectrum activity. For example, compounds **2**–**4** showed modest potency improvements relative to nirmatrelvir against α‐CoV (CCoV‐HuPn‐2018) and δ‐CoV (PDCoV) 3CL^pro^s. Therefore, such a systematic study on the sequence and structure conservation of all 3CL^pro^s and, in particular, the identification of different conservative features of S1'/S1/S2/S4 subsites at the substrate binding pockets provides valuable structural insights for the rational design of pan‐coronavirus inhibitors targeting 3CL^pro^, with P2 optimization emerging as the key determinant for achieving broad‐spectrum inhibition.

Currently, the broad‐spectrum 3CL^pro^ inhibitors have primarily emerged from previously developed inhibitors targeting the 3CL^pro^s of SARS‐CoV, MERS‐CoV, and SARS‐CoV‐2. They are known to inhibit the 3CL^pro^s of several other CoVs, such as the other four HCoVs (HCoV‐HKU1, HCoV‐OC43, HCoV‐229E, and HCoV‐NL63), however, their effectiveness across the full spectrum of known CoV species has not been exhaustively evaluated. As a result, the feasibility of rational design to identify universally effective inhibitors against the 3CL^pro^s of all 26 subgenera of the four genera remains elusive. The broad‐spectrum inhibitory activities of three approved SARS‐CoV‐2 3CL^pro^ inhibitors, nirmatrelvir, simnotrelvir, and ensitrelvir, were first assessed against fifteen 3CL^pro^s from CoVs of concern across four genera in this study, revealing a pronounced potency against β‐CoV 3CL^pro^s but significantly lower activity against α‐, γ‐, and δ‐CoV 3CL^pro^s. Utilizing bioinformatic analysis and structural insights, we found pivotal variations of the S2 and S4 subsites that limit the broad‐spectrum activity of approved 3CL^pro^ inhibitors. To address these limitations, we employed a structure‐guided approach to develop novel covalent inhibitors. Compounds **1** and **6**, featuring a nitrile warhead, exhibited potent and broad enzymatic inhibition across multiple coronavirus genera, validating the compatibility of nitrile‐based covalent warheads with diverse 3CL^pro^ active sites. However, their limited membrane permeability constrained their antiviral efficacy evaluation in cell‐based assays. To overcome this, we replaced the nitrile warhead with an aldehyde moiety of comparable size and reactivity but with improved physicochemical properties, leading to the development of compound **8**. Such a substitution markedly enhanced the cellular potency of the compound against α‐, β‐, γ‐, and δ‐coronaviruses, although it resulted in a slight reduction in selectivity, particularly against host cathepsin B/L. Finally, the in vivo antiviral efficacy was demonstrated with the prodrug form of compound **8**. Importantly, compound **8** demonstrates promising broad‐spectrum inhibition of 3CL^pro^s but remains an early‐stage lead compound rather than an optimized clinical candidate, requiring further optimization of drug‐like properties such as pharmacokinetic profile. Subsequent work will focus on systematic exploration of the structure‐activity relationship through modifications to the warhead and P2/P4 segments, aiming to improve pharmacokinetic properties while preserving or enhancing the broad‐spectrum 3CL^pro^ inhibitory profile.

Despite these limitations, compounds **6** and **8** exhibited unprecedented inhibitory breadth, effectively targeting 32 distinct 3CL^pro^s across all 26 subgenera of the four coronavirus genera, including emerging and zoonotic strains such as CCoV‐HuPn‐2018 and PDCoV. This marks the first exploration of truly ultra‐broad‐spectrum 3CL^pro^ inhibitors. More importantly, our study provides fundamental insights into the design principles required to achieve such breadth. Rather than merely identifying potent candidates, we establish a comprehensive and robust structure‐based framework—integrating cross‐genus enzymatic and cellular inhibition profiling, co‐crystal structure determination, and rational optimization of covalent and non‐covalent interactions. Our results demonstrate that with precise molecular design, it is indeed feasible to create inhibitors capable of broadly inhibiting the entire coronavirus family. These findings represent a major step toward the development of next‐generation pan‐coronavirus therapeutics with translational potential.

## 4 Experimental Section

### Chemistry

Procedure and synthetic schemes for compounds **1–10** are shown in Figures S10‐S13 (Supporting Information). Spectral data for compounds **1–10** were included in the Spectral Data for Synthetic Compounds section of the SupportingInformation. All commercially available chemicals and solvents were used directly without further purification. All reactions were monitored by thin‐layer chromatography (TLC) on silica gel plates (GF‐254). All compounds were characterized by their NMR and MS spectra. ^1^H NMR and ^[^ ^13^ ^]^C NMR spectra were recorded on a Bruker Avance 600 or a Bruker Avance 500 using TMS as the internal standard. NMR data were recorded using TopSpin software (v3.0 or v3.2) and analyzed using MestReNova (v12.0.0‐20080). HPLC traces were collected and analyzed on an Elite 3150 system using Chromsoft software (v8.2.0.82). High‐resolution mass spectrometry (HRMS) spectra were acquired with an Agilent G6520 Q‐TOF mass spectrometer using MassHuter Workstation software (vB.05.01) and analyzed with Xcalibur software (v2.0.5). Low‐resolution mass spectrometry (LRMS) spectra were acquired with a Thermo Fisher FINNIGAN LTQ spectrometer and analyzed using Xcalibur software (v2.0.5). The purities of compounds used in the biochemical tests were over 95%. Purity was determined by the following method: column, WondaSil C18 Superb (5 µm, 4.6 mm × 250 mm); mobile phase A: water containing 0.1% trifluoroacetic acid (TFA); mobile phase B: acetonitrile containing 0.1% trifluoroacetic acid (TFA); gradient, 10% B–100% B over 15 min, 100% B for 5 min, 100% B–10% B over 5 min; flow rate, 1.0 mL min^−1^; detective wavelength, 220 nm; column temperature, 25 °C.

### Protein Expression and Purification

The cDNA sequences encoding 32 3CL^pro^s of various coronaviruses, each tagged with an N‐terminal SUMO for enhanced solubility and purification ease, were cloned into the pET‐15b expression vector. The GenBank codes for each coronavirus under study are detailed in Figure S1 (Supporting Information). Mutations in the 3CL^pro^s, tailored for isothermal titration calorimetry (ITC) analysis, were introduced using the restriction‐free cloning method. These modified plasmids were subsequently transformed into *Escherichia coli* BL21 (DE3) cells for protein expression. The expressed 3CL^pro^ proteins were initially purified using nickel‐nitrilotriacetic acid (Ni‐NTA) column chromatography (GE Healthcare). This step was followed by the removal of the SUMO tag using SUMO‐specific peptidase 2 (SENP2), ensuring the retrieval of the native protein sequence. Final purification stages involved anion exchange chromatography with Q‐sepharose and size‐exclusion chromatography (GE Healthcare), to achieve high‐purity protein samples. For enzymatic inhibition assays and protein crystallization, proteins were stored in a buffer containing 10 mm Tris, pH 7.5, and 2 mm DTT. Alternatively, for ITC measurements, the proteins were suspended in a 50 mm Tris buffer at pH 7.5, with an added 4 mm 2‐mercaptoethanol for the specific case of PDCoV and CCoV‐HuPn‐2018 3CL^pro^s, to ensure stability and hydrolytic activity of the protease throughout the measurements.

### 3CL^pro^ and PL^pro^ Enzymatic Inhibition Assays

The inhibitory activities of compounds against 3CL^pro^s were determined by fluorescence resonance energy transfer (FRET) protease assays. The fluorogenic substrate DABCYL‐KTSAVLQSGFRKME‐EDANS (GenScript, China) can be cleaved by 3CL^pro^, producing an EDANS peptide fragment that emits an intense fluorescence signal at excitation/emission wavelengths of 340/490 nm. The FRET‐based enzymatic assay was performed as follows. Recombinant 3CL^pro^ at a final concentration of 100 nm was mixed with serial dilutions of each compound in 80 µL of assay buffer (50 mm Tris, pH 7.3, and 1 mm EDTA) and incubated for 10 min at room temperature. The reaction was initiated by adding 40 µL of fluorogenic substrate to a final concentration of 10 µm. After that, the fluorescence signal at 340 nm (excitation)/490 nm (emission) was immediately measured every 1 min for 10 min with a microplate reader. The initial velocities of the reactions with added compounds compared to the reaction with DMSO were calculated and used to plot IC_50_ curves. At least six concentrations of each compound were used to determine the IC_50_ values that were calculated by GraphPad Prism (v9.1.2). Three independent experiments, each carried out in triplicate, were performed to determine the IC_50_ values of the compounds against PDCoV and CCoV‐HuPn‐2018 3CL^pro^s. The IC_50_ values were expressed as the mean ± SD. Experiments were performed in triplicate to determine the IC_50_ values of the compounds against 3CL^pro^s of other CoVs.

The inhibition assay for SARS‐CoV‐2 PL^pro^ was conducted in the following manner. Recombinant PL^pro^, at a final concentration of 50 nm, was incubated with the tested compound in 80 µL of assay buffer (50 mm HEPES, pH 7.5, and 0.1 mg mL^−1^ Bovine Serum Albumin [BSA]) for 30 min at room temperature. The enzymatic reaction was then initiated by the addition of 40 µL of the fluorogenic substrate RLRGG‐AMC, reaching a final concentration of 20 µm. Subsequently, the fluorescence signal, with an excitation wavelength of 360 nm and an emission wavelength of 460 nm, was measured at 1‐min intervals for a duration of 10 min using a microplate reader. The inhibitory potency of the compound was quantified by comparing the reaction rates in the compound‐containing wells to those in the DMSO‐containing wells, thereby determining the inhibition ratio. All experiments were conducted in triplicate.

### Enzymatic Inhibition Assays of Mammalian Proteases

Cathepsin B (2 nm), cathepsin L (20 nm), cathepsin G (5 nm), and bovine chymotrypsin (5 nm) were respectively added to plates containing compounds and incubated at room temperature for 10–30 min. The reactions were started by the addition of 2 µm cathepsin B substrate Z‐RR‐AMC, 20 µm cathepsin L substrate Z‐FR‐AMC, 10 µm cathepsin G substrate Suc‐AAPF‐AMC, and 10 µm chymotrypsin substrate Suc‐AAPF‐AMC, respectively. The reactions proceeded for ≈10–60 min and were monitored by a microplate reader.

The caspase 6 inhibition assay was meticulously executed using a kit provided by BPS Bioscience (catalog number 80703), following the detailed instructions supplied by the manufacturer. The procedure was conducted in a total volume of 50 µL, starting with a mixture of the tested compound and the caspase 6 substrate (a final concentration of 1 µm) in 30 µL assay buffer enriched with 5 mm DTT. The reaction was initiated by adding 20 µL of a diluted caspase 6 solution to a final concentration of 0.125 ng µL^−1^. This mixture was incubated at room temperature for 30 min. After that, the fluorescence emission was measured using a microplate reader at an excitation of 380 nm and an emission of 500 nm. The reaction rates of the wells containing the compound were compared to those of the wells containing DMSO to determine the inhibition ratio. Experiments were performed in triplicate.

### ITC Measurements

ITC experiments were conducted at 20 °C to obtain the thermodynamic binding profile of each compound with SARS‐CoV‐2 3CL^pro^ C145G, PDCoV 3CL^pro^ C144G, and CCoV‐HuPn‐2018 3CL^pro^ C144G, respectively. All measurements were performed in ITC buffer containing 50 mm Tris (pH 7.5) and 4 mm 2‐mercaptoethanol with an iTC_200_ calorimeter (GE Healthcare). Compounds were diluted in the ITC buffer to final concentrations of 0.5–3 mm. The purified SARS‐CoV‐2 3CL^pro^ C145G, PDCoV 3CL^pro^ C144G, and CCoV‐HuPn‐2018 3CL^pro^ C144G were diluted in the ITC buffer to final concentrations of 0.05–0.3 mm. The final concentration of DMSO in the reaction was less than 1%. All titrations were performed using an initial injection of 0.4 µL followed by 19 identical injections of 2 µL each with a duration of 4 s per injection and a spacing of 120 s between injections. The last three data points were averaged as the heat of dilution and subtracted from each titration. Three independent experiments were carried out to ensure accuracy and reproducibility. The values were expressed as the mean ± SD of three independent experiments.

### Protein Crystallization and Structure Determination

The purified CCoV‐HuPn‐2018, SARS‐CoV‐2, IBV, and PDCoV 3CL^pro^s were concentrated to ≈9–20 mg mL^−1^ for crystallization. The 3CL^pro^s were incubated with 1–4 mm inhibitors for 1 h before crystallization. Crystals of CCoV‐HuPn‐2018 3CL^pro^ in complex with the inhibitor were obtained under the conditions of 12% PEG3350 and 100 mm sodium malonate, pH 5.0. Crystals of SARS‐CoV‐2 3CL^pro^ in complex with the inhibitor were obtained under the conditions of 2–8% PEG6000, 100 mm MES, pH 6.0–7.25, and 3% DMSO. Crystals of IBV 3CL^pro^ in complex with the inhibitor were obtained under the conditions of 20% PEG3350, 100 mm Bis‐Tris propane, pH 7.5, and 200 mm potassium thiocyanate. Crystals of PDCoV 3CL^pro^ in complex with the inhibitor were obtained under the conditions of 0.5–2% PEG6000 and 100 mm sodium citrate, pH 4.6–5.25. All crystals were flash frozen in liquid nitrogen in the presence of the reservoir solution supplemented with 20% glycerol. X‐ray diffraction data were collected at beamlines BL02U1 and BL10U2 at the Shanghai Synchrotron Radiation Facility. The diffraction data were auto‐processed by the Aquarium pipeline.^[^ ^20^ ^]^ The complex structures were solved by molecular replacement using the program CCP4 (v7.0.078).^[^ ^21^ ^]^ The final complex structures were manually refined with COOT (v0.8.9.2)^[^ ^22^ ^]^ and PHENIX (v1.17.1–3660),^[^ ^23^ ^]^ and further analyzed with PyMol (v2.4.0). The refined structures were deposited in the Protein Data Bank with the accession codes listed in Table S1 (Supporting Information). The complete statistical data, as well as the quality of the solved structures, are also shown in Table S1 (Supporting Information). 2*Fo*‐*Fc* electron density maps of the bound compounds are shown in Figure S14 (Supporting Information).

### Bioinformatic Analysis and Structural Clustering of CoV 3CL^pro^s

Amino acid sequences of 59 CoV 3CL^pro^s were retrieved from the National Center for Biotechnology Information (NCBI) database, referencing their specific GenBank accession numbers detailed in Figure S1 (Supporting Information). A sequence pairwise similarity matrix for these 59 3CL^pro^s and the phylogenetic tree was constructed using TBtool,^[^ ^24^ ^]^ facilitating a comparative analysis of their genetic relationships. For those 3CL^pro^s lacking available crystal structures, 3D models were predicted using ColabFold (v1.5.5).^[^ ^25^ ^]^ This advanced prediction method integrates MMseqs2 for sequence searching and AlphaFold2 for accurate structure estimation. Alignments of both crystal and predicted structures were carried out using PyMol (v2.4.0), enabling a detailed structural comparison. The sequence and structural data pertaining to the substrate binding pockets of 32 selected CoV 3CL^pro^s were then meticulously extracted for in‐depth analysis. In terms of the clustering analysis of the structural data, using the crystal structure of SARS‐CoV‐2 3CL^pro^ in complex with nirmatrelvir (PDB ID: 7RFW) as a reference, residues within 8 Å of the bound inhibitor and representing complete secondary structure elements were selected, resulting in the following residues, 25–28, 39–53, 84, 117–119, 139–147, 161–174, and 181–193. Local paired structure similarity assessments were conducted using US‐align^[^ ^26^ ^]^ based on TM‐score normalized by the average length of the structures, revealing intricate details of pocket conservation and variability. A dendrogram of a structural similarity matrix was constructed employing the clustering method Unweighted Pair Group Method with Arithmetic Mean (UPGMA) via DendroUPGMA.^[^ ^27^ ^]^ The unrooted tree of the sequence similarity matrix and the circular tree of the structural similarity matrix were both visualized with the Interactive Tree Of Life (iTOL) software (v6.8.2).^[^ ^28^ ^]^ Heatmap diagrams, illustrating various analyzed aspects of the study, were generated in TBtool,^[^ ^24^ ^]^ whereas protein structure diagrams were expertly crafted in PyMol (v2.4.0).

### Luciferase‐Based Intracellular 3CL Protease Reporter Assay

The ODD‐luciferase pseudovirus assay was used to assess the inhibitory potency of compounds against α‐CCoV‐HuPn‐2018, β‐SARS‐CoV‐2, γ‐IBV, and δ‐PDCoV 3CL^pro^s at the cellular level. In this assay, the N‐terminus of the reporter enzyme *Renilla reniformis* luciferase was connected via a 3CL^pro^ cleavage site (VARLQSGF) to the HIF‐1α oxygen‐dependent degradation domain (ODD). When the fusion protein was not cleaved by 3CL^pro^, the ODD domain initiates rapid degradation of the fusion protein by the proteasome, resulting in a decrease in luciferase activity. Thus, the luminescence intensity reflects the inhibition degree of the compound against 3CL^pro^s.

The assay was carried out as follows: HEK293T (RRID: CVCL_0063) cells, purchased from ATCC, were seeded into 6‐well plates and incubated overnight to reach 70–80% confluency. 60 ng of human codon‐optimized 3CL^pro^ (WT or catalytic inactive mutant) plasmid and 1500 ng luciferase‐based reporter plasmid transient transfer to cells were co‐transfected into each well with transfection reagent X‐tremeGENE (Roche). The cells were trypsinized and seeded into 96‐well plates (30000 cells per well) after 3 h of transfection. After that, the medium containing the compound at different concentrations was added. After incubation for 20 h, the medium was removed, and cells were lysed by adding 120 µL assay buffer (40 mm Tris, pH 7.8, 2 mm MgCO_3_, 5 mm MgSO_4_, 2 mm EDTA, 4 mm DTT, 1% Triton X‐100, 250 µm ATP, and 200 µm luciferase substrate). 80 µL cell lysate was added to white 96‐well plates to record the luciferase intensity using a microplate reader. Percentage inhibition was normalized to the luciferase intensity of the DMSO control wells. The EC_50_ values were calculated by GraphPad Prism (v9.1.2). Three independent experiments (each in duplicate) were performed. Seven concentrations of each compound were used to determine the EC_50_ values. The EC_50_ values were expressed as the mean ± SD.

### Cell‐Based Antiviral Assays

RD (RRID: CVCL_1649), and Huh‐7 (RRID: CVCL_0336) cell lines were purchased from the cell bank of CAS, hACE2‐expressing HEK293T (HEK293T‐hACE2) cells were kindly provided by Dr. Lanying Du. All cell lines were cultured in Dulbecco's Modified Eagle Medium (DMEM; Gibco Invitrogen) supplemented with 10% fetal bovine serum (FBS; Gibco Invitrogen), 1% antibiotic/antimycotic (Gibco Invitrogen), at 37 °C in a humidified 5% CO_2_ incubator. For SARS‐CoV‐2 variants, HEK293T‐hACE2 cells were pre‐seeded to 48‐well plates (5 × 10^4^ cells well^−1^). RD and Huh‐7 cells were pre‐seeded to 48‐well plates (50 000 cells well^−1^) for HCoV‐OC43 and HCoV‐229E infection. To evaluate the anti‐SARS‐CoV‐2 activity of compound **8**, pre‐seeded HEK293T‐hACE2 cells (5 × 10^4^ cells well^−1^) were treated with the different concentrations of the compound for 1 h, and then were infected with SARS‐CoV‐2 Delta (B.1.617.2), Omicron BA.5, and Omicron JN.1 at a MOI of 0.05. One hour later, the supernatant was removed, and cells were further cultured with drug containing medium. At 24 h p.i., the supernatant was collected for viral RNA copy number determination using real‐time fluorescence quantitative PCR (qRT‐PCR). The primers of qRT‐PCR of SARS‐CoV‐2 variants were RBD‐qF1: 5′‐CAATGGTTTAACAGGCACAGG‐3′ and RBD‐qR1: 5′‐CTCAAGTGTCTGTGGATCACG‐3′. To assess the anti‐HCoV‐OC43 and anti‐HCoV‐229E activity of compound **8**, pre‐seeded RD or Huh‐7 cells (5 × 10^4^ cells well^−1^) were treated with the different concentrations of compound **8** for 1 h, and then were infected with HCoV‐OC43 or HCoV‐229E at an MOI of 0.1, followed by removing the virus‐drug mixture after 2 h, and cells were further cultured with drug containing medium. At 48 h p.i., the supernatant was collected for viral RNA copy number determination using qRT‐PCR. The primers of qRT‐PCR of OC43 were OC43‐NP‐F: 5′‐CGATGAGGCTATTCCGACTAGGT‐3′ and OC43‐NP‐R: 5′‐CCTTCCTGAGCCTTCAATATAGTAACC‐3′, of 229E were 229E‐NP‐F: 5′‐ CAGTCAAATGGGCTGATGCA‐3′ and 229E‐NP‐R: 5′‐ AAAGGGCTATAAAGAGAATAAGGTATTCT‐3′. Three independent experiments were performed to determine the EC_50_ of compound **8** against each viral strain, and EC_50_ values were fitted and calculated in GraphPad Prism (v9.1.2). All viral strains in this study were obtained from the National Virus Resource Center, Chinese Academy of Sciences (CAS). All experiments with authentic SARS‐CoV‐2 viruses were carried out in the Biosafety Level 3 facility (BSL‐3) of the Wuhan Institute of Virology, Chinese Academy of Sciences (CAS).

### Cytotoxicity Test

HEK293T (RRID: CVCL_0063) cells, purchased from ATCC, were plated into 96‐well plates (30000 cells per well) to perform cell viability experiments. All compounds were diluted 2‐fold across 7 concentrations starting at 200 or 400 µm in maintenance medium, with each concentration tested in triplicate. After incubation for 24 h, 10 µL of WST‐8 reagent (Yeasen Biotechnology) was added to each well. Following a 1 h incubation at 37 °C, absorbance at 450 nm was recorded using a microplate reader, and cell viability was calculated.

### Cell Membrane Permeability Experiment

The intracellular concentrations of compound **6** and nirmatrelvir, mainly reflecting the equilibrium between membrane penetration and intracellular degradation of the compounds, were determined in HEK293T (RRID: CVCL_0063, purchased from ATCC) cells. One million cells were plated into a 6‐well plate and incubated overnight to reach 95% confluency. Then, the test compound was added to the culture medium (DMEM supplemented with 10% FBS) at a final concentration of 100 µm. After 8 h of incubation, cells were harvested and washed with phosphate‐buffered saline (PBS). The resulting cell pellets were then resuspended in 70% methanol solution and subjected to boiling at 95 °C for 5 min. The boiled suspension was cooled to ambient temperature and centrifuged at 12000 rpm for 10 min to separate cell debris from the solvent extract. The supernatants were transferred into new tubes, and the solvent was evaporated overnight at 70 °C. 50 µL DMSO was added to the dried tubes, followed by a 1 h incubation at room temperature with shaking. Samples were then analyzed using TOF LC/MS. The amount of compound obtained in the extracts was determined by comparison to the standards of each purified compound in DMSO.

### In Vivo Pharmacokinetics Study of Compound 10 in Mice

For pharmacokinetic studies in mice, experiments (dosing and blood sample collection) were carried out at the Shanghai Institute of Materia Medica (SIMM). Six ICR male mice, weighting 18–22 g each were randomly divided into two groups. The first group received an intravenous injection of compound **10** at a dose of 10 mg kg^−1^. The second group received an oral dose of compound **10** at 200 mg kg^−1^ combined with or without ritonavir at 50 mg kg^−1^. Blood samples at various time points were also collected. Plasma concentrations of **10** were analyzed using LC‐MS/MS. The animal experiments were in accordance with regulations and established guidelines and were reviewed and approved by the Institutional Animal Care and Use Committee of SIMM (SYXK2020‐0042), following the National Institutes of Health Guidelines for the Care and Use of Experimental Animals (WIVA25202202).

### In Vivo Efficacy against HCoV‐OC43 in Suckling Mice

The BALB/c mice were bred and maintained in a specific‐pathogen‐free (SPF) environment at the Laboratory Animal Center of Wuhan Institute of Virology, CAS. The pregnant mice with the same expected delivery date were acclimated in individually ventilated cages in an SPF environment under standard conditions. On the 5th day after birth, suckling mice were divided randomly into 4 groups, namely, the vehicle group, the group receiving compound **10** 500 mpk with ritonavir 50 mpk, the group receiving compound **10** 250 mpk with ritonavir 50 mpk, and the group receiving nirmatrelvir 50 mpk. All groups were orally treated by vehicle or compounds quaque die (QD). Mice were anesthetized by isoflurane inhalation and then intranasally infected with 1 × 10^4^ TCID50 of HCoV‐OC43. One hour after viral infection, mice were orally treated with vehicle or compounds according to the group description as described above (day 0). Mice were orally treated in the following days. On Day 5, every mouse in each group was sacrificed for tissue collection. Multiple organs and tissues from mice, including brains, spinal cords, and lungs, were collected for viral copy determination. Part of the organs and tissues were homogenized with DMEM and subsequently centrifuged at 3000 rpm for 10 min at 4 °C. Viral and host RNA from the organs and tissues were extracted with the RNeasy Mini Kit (Qiagen) and reverse transcribed (PrimeScript Reverse Transcriptase, Takara) according to the operation instruction, then the absolute viral RNA copy in the organs and tissues was detected quantitatively by real‐time fluorescence quantitative PCR. The viral RNA copies were calculated by the concentration of standard plasmids for the HCoV‐OC43 nucleocapsid proteins. The animal experiments were in accordance with regulations and established guidelines and were reviewed and approved by the Institutional Review Board of the Wuhan Institute of Virology (SYXK2022‐0131), following the National Institutes of Health Guidelines for the Care and Use of Experimental Animals (WIVA25202202). Viral infections were performed in an animal biosafety level 2 (BSL‐2) facility.

### Statistical Analysis

All statistical analyses were performed according to standard procedures for biochemical, cellular, and in vivo studies. Quantitative data from biochemical and cellular assays were presented as mean ± SD, whereas data from in vivo antiviral experiments were presented as mean ± SEM. Sample sizes were defined as follows: enzymatic inhibition assays of CCoV‐HuPn‐2018 and PDCoV 3CL^pro^s were conducted in three independent biological experiments (n = 3), each consisting of three technical replicates, assays of other 3CL^pro^s and proteases were conducted with three technical replicates at each inhibitor concentration; pseudovirus assays were performed in three independent biological experiments (n = 3), each consisting of two technical replicates; cellular antiviral assays were performed in three independent biological experiments (n = 3), each consisting of three technical replicates; ITC measurements were carried out as three independent titrations for each compound–protein pair (n = 3); and the in vivo antiviral study initially included five mice per group (n = 5), with final group sizes reduced due to filial cannibalism, as indicated in the corresponding figure legends. Statistical hypothesis testing was applied only to the in vivo antiviral data. Comparisons between two groups were performed using a two‐tailed unpaired Student's *t*‐test, with statistical significance defined as ^*^ *P* < 0.05, ^**^ *P* < 0.01, and ^***^ *P* < 0.001. All statistical analyses and curve fitting were performed using GraphPad Prism (v9.1.2).

## Conflict of Interest

The authors declare no conflict of interest.

## Author Contributions

Haixia Su, Tianqing Nie, Guofeng Chen, Muya Xiong, and Yumin Zhang contributed equally to this work. Y.X. (Xu) and H.S. conceived and designed the project. Y.X. (Xu) and H.S. designed the experiments; Y.X. (Xu), H.S., and G.C. performed the drug design; G.C., J.H., and G.W. performed the chemical experiments and collected the data; M.X. performed bioinformatic analysis and structural clustering; H.S., T.N., M.Y., H.X., Y.X. (Xiong), H.H., W.Z. and M.L. performed protein expression and purification, crystallization, X‐ray diffraction data collection, solved and analyzed the crystal structures; T.N. and H.S. performed enzymatic assays and determined the IC_50_ values of the compounds; T.N. and H.S. performed the Luciferase‐based intracellular 3CL^pro^ reporter assays; Y.Z., G.X. and L.Z. performed the authentic virus assays and in vivo efficacy assessment; Y.X. (Xu) and H.S. wrote and revised the manuscript with input from all other authors.

## Supporting information



Supporting Information

## Data Availability

The data that support the findings of this study are available from the corresponding author upon reasonable request.
