# Supplementary material for: Structure‐Based Development of Ultra‐Broad‐Spectrum 3C‐Like Protease Inhibitors
Source: Adv Sci (Weinh). 2025 Dec 12;13(11):e12342. doi: 10.1002/advs.202512342 (PMC12931257; doi:10.1002/advs.202512342)

**Supporting Information**

**Structure-Based Development of** **Ultra-Broad-Spectrum 3C-Like Protease Inhibitors**

Haixia Su^2,4,9^*, Tianqing Nie^2,5,7,9^, Guofeng Chen^1,2,9^, Muya Xiong^1,2,9^, Yumin Zhang^3,9^, Guoqing Wu^1,2^, Mengyuan You^2^, Hang Xie^2^, Jian He^2,4^, Yanchao Xiong^2,4^, Hangchen Hu^2,4^, Wenfeng Zhao^2,4^, Minjun Li^8^, Gengfu Xiao^3^, Leike Zhang^3,6^*, Yechun Xu^1,2,4^*

^1^School of Pharmaceutical Science and Technology, Hangzhou Institute for Advanced Study, University of Chinese Academy of Sciences, Hangzhou 310024, China

^2^State Key Laboratory of Drug Research, Shanghai Institute of Materia Medica, Chinese Academy of Sciences, Shanghai 201203, China

^3^State Key Laboratory of Virology, Wuhan Institute of Virology, Center for Biosafety Mega-Science, Chinese Academy of Sciences, Wuhan, Hubei, 430071, China

^4^University of Chinese Academy of Sciences, Beijing 100049, China

^5^Lingang Laboratory, Shanghai 200031, China

^6^Hubei Jiangxia Laboratory, Wuhan, 430200, China

^7^School of Physical Science and Technology, ShanghaiTech University, Shanghai 201210, China

^8^Shanghai Synchrotron Radiation Facility, Shanghai Advanced Research Institute, Chinese Academy of Sciences, Shanghai 201204, China

^9^These authors contributed equally

*Correspondence: suhaixia1@simm.ac.cn; zhangleike@wh.iov.cn; [ycxu@simm.ac.cn](mailto:ycxu@simm.ac.cn)

**Table of Contents**

|  | **Initial page** |
| --- | --- |
| **Supplementary Figures** | **S3** |
| **Supplementary Tables** | **S17** |
| **Spectral Data for Synthetic Compounds** | **S26** |

# S1: Supplementary Figures


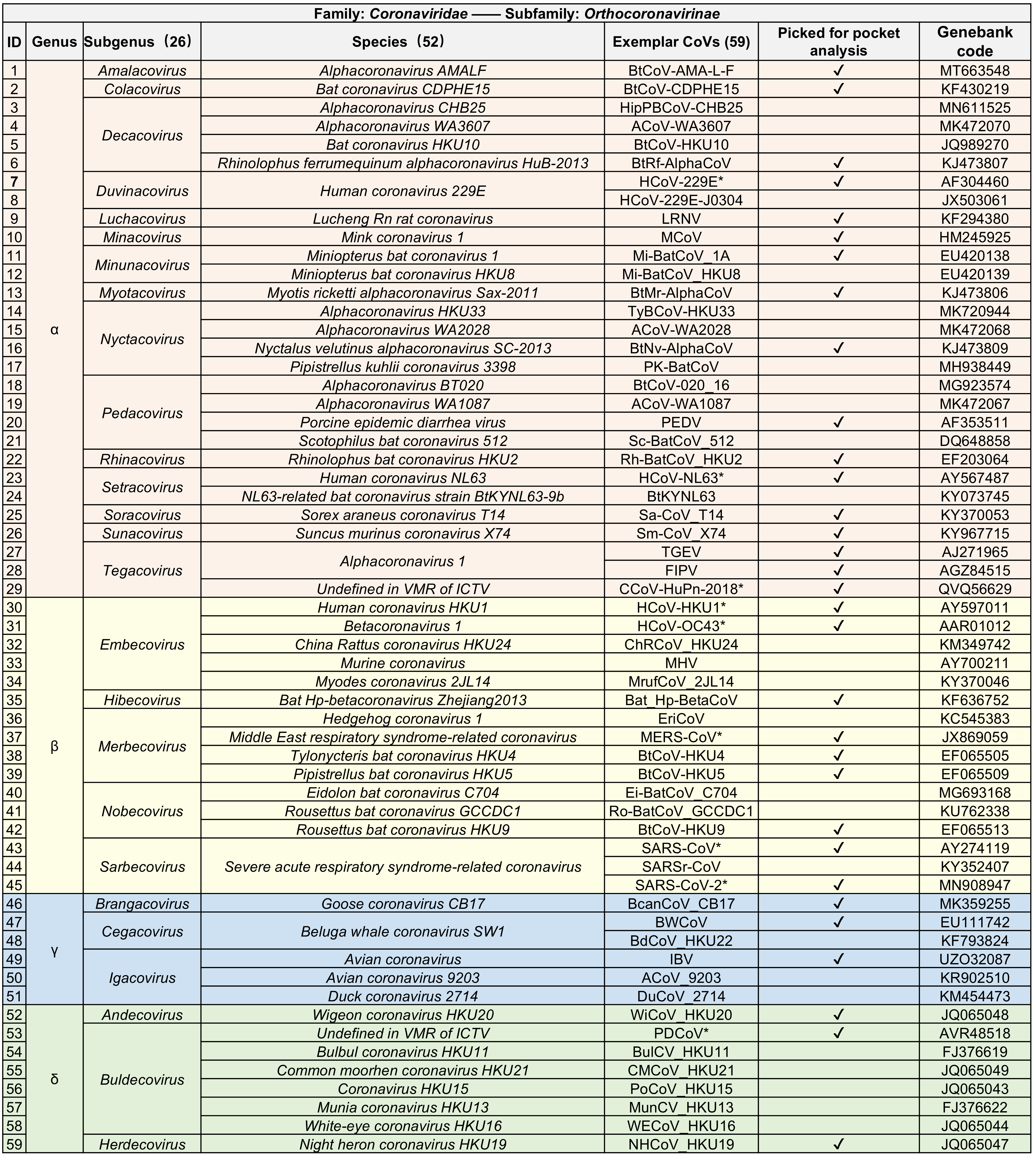


**Figure S1.** **Taxonomic classification of 59 representative coronaviruses.** Nine HCoVs are marked with asterisks. Coronaviruses selected for the conservation investigation of 3CL^pro^ substrate binding pocket are marked with checkmarks.


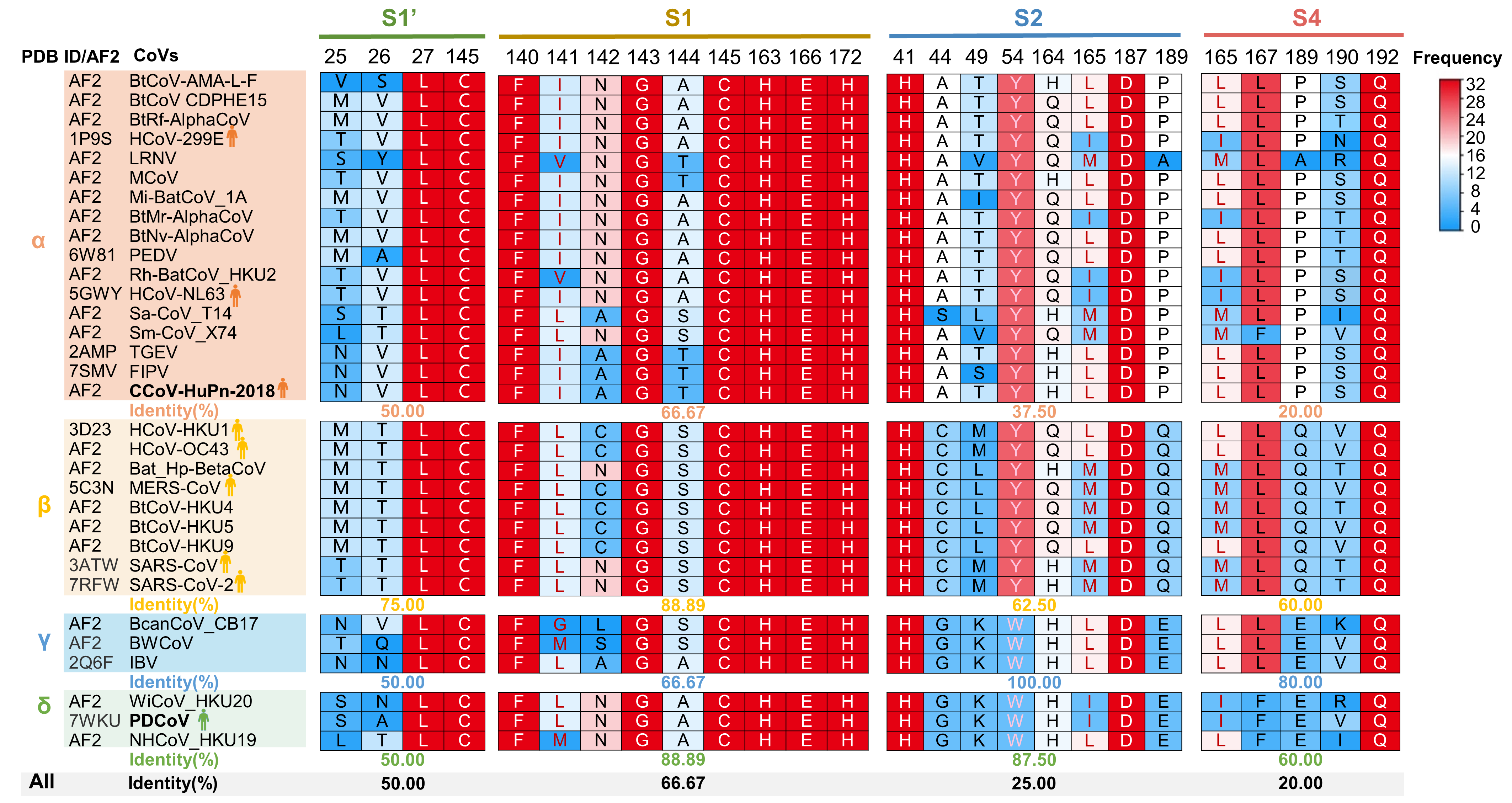


**Figure S2. 3D structure-based sequence alignment map depicting the conservation of residues at four subsites (S1ʹ, S1, S2, and S4) of selected 32 3CL^pro^s across 26 subgenera.** Heatmap colors represent the frequency of the residue appeared in 32 3CL^pro^s. 3D structures of 3CL^pro^s predicted by AlphaFold2 are marked as ‘AF2’, otherwise PDB codes are provided. HCoVs are marked with man icons. The residue numberings are for SARS-CoV-2 3CL^pro^.


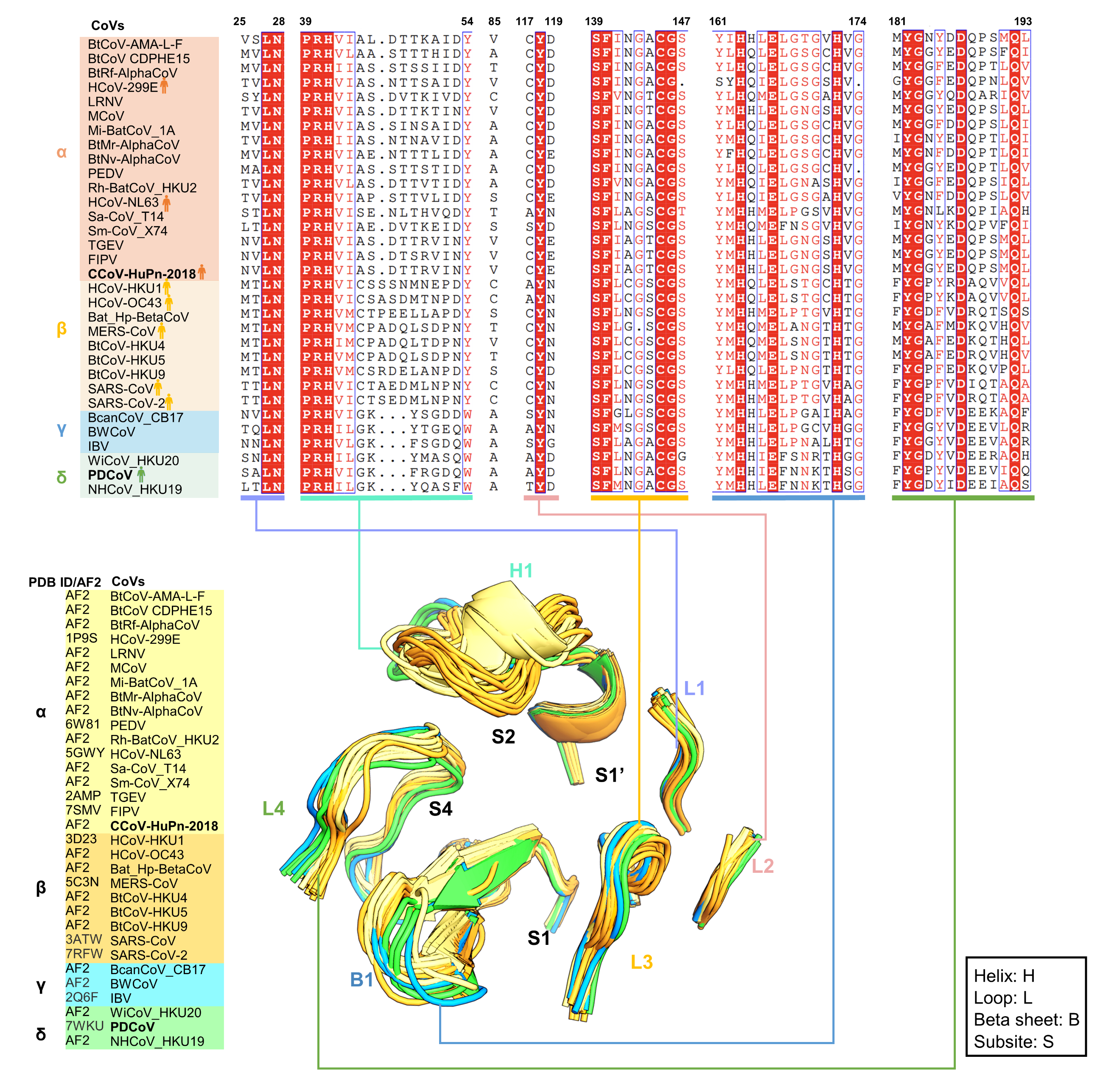


**Figure S3. Structure and sequence alignment map of substrate binding pockets across 32 3CL^pro^s.** Four genera of CoVs are distinguished by colors: orange (α), yellow (β), blue (γ), and green (δ). 3D structures of 3CL^pro^s predicted by AlphaFold2 are marked as ‘AF2’, otherwise PDB codes are provided. The residue numberings are for SARS-CoV-2 3CL^pro^.


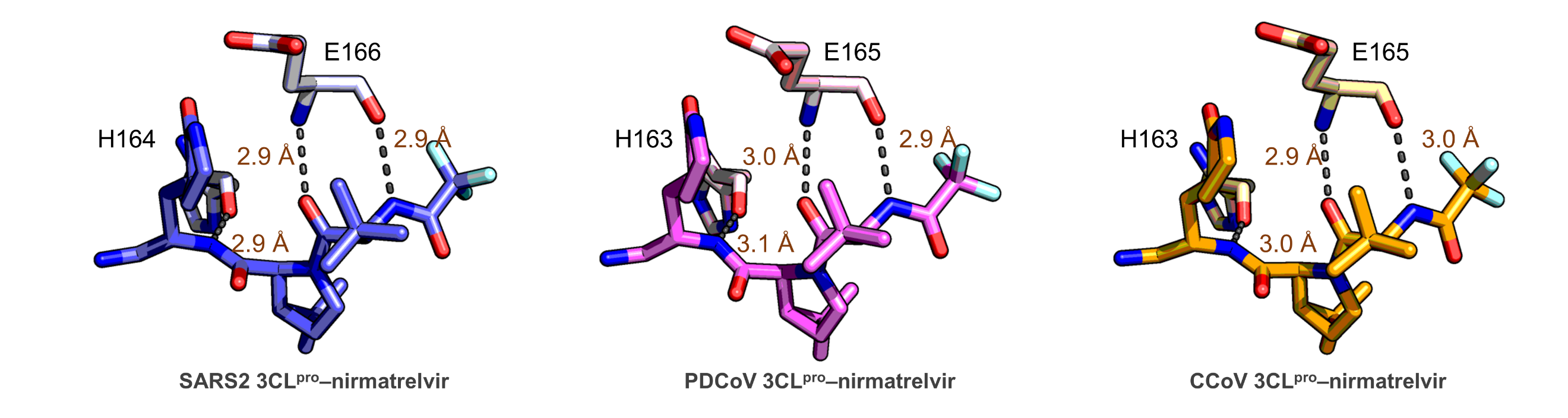


**Figure S4. H-bonds formed between nirmatrelvir and three different 3CL^pro^s.** Nirmatrelvir and residues are shown as sticks, and the H-bonds are represented by dashed lines.


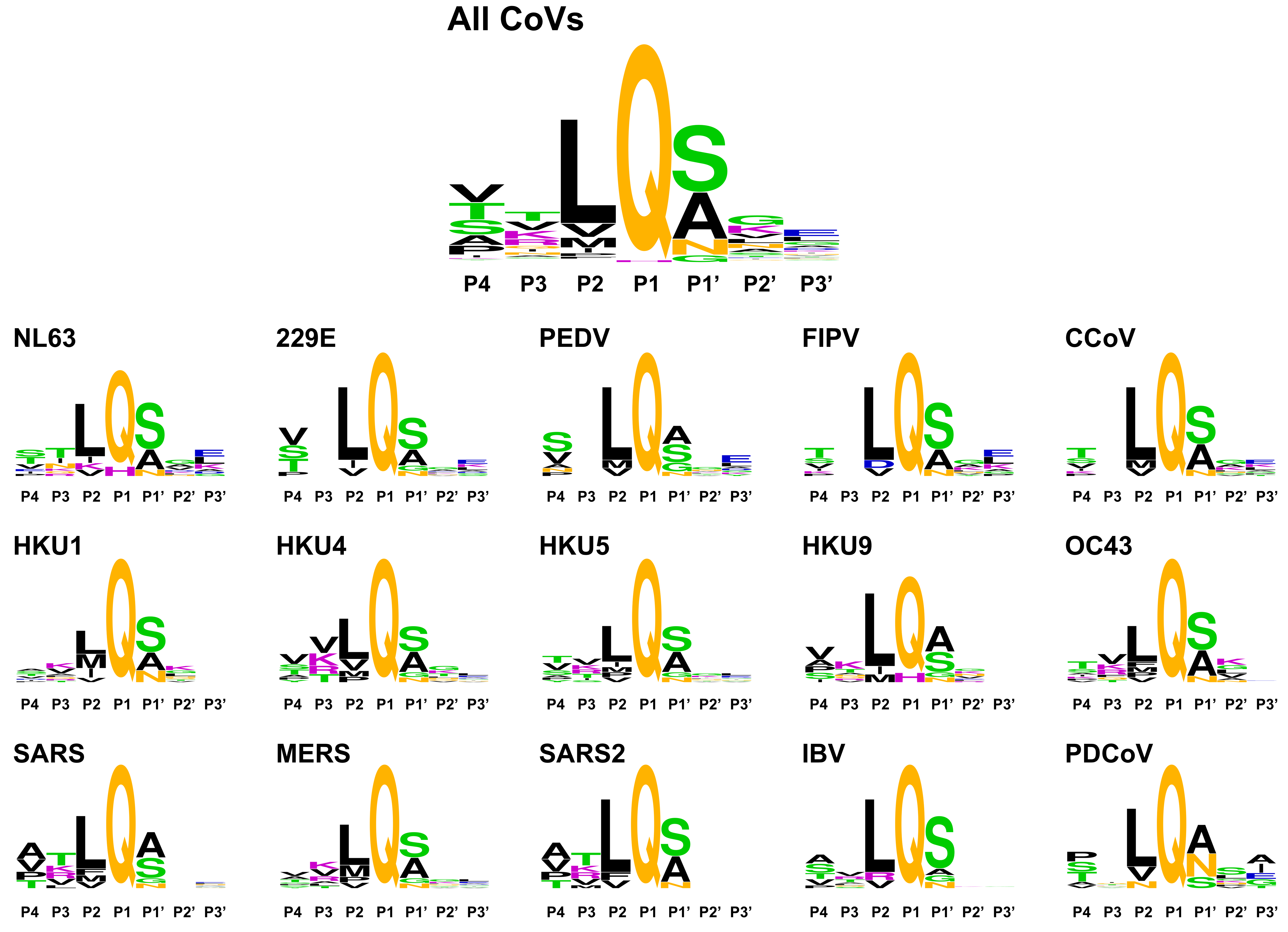


**Figure S5. Substrate bias across diverse CoV 3CL^pro^s from four genera in broad-spectrum profiling assays**. Sequence logos indicating the amino acid specificity in the cleavage site of substrates for 15 CoV 3CL^pro^s.


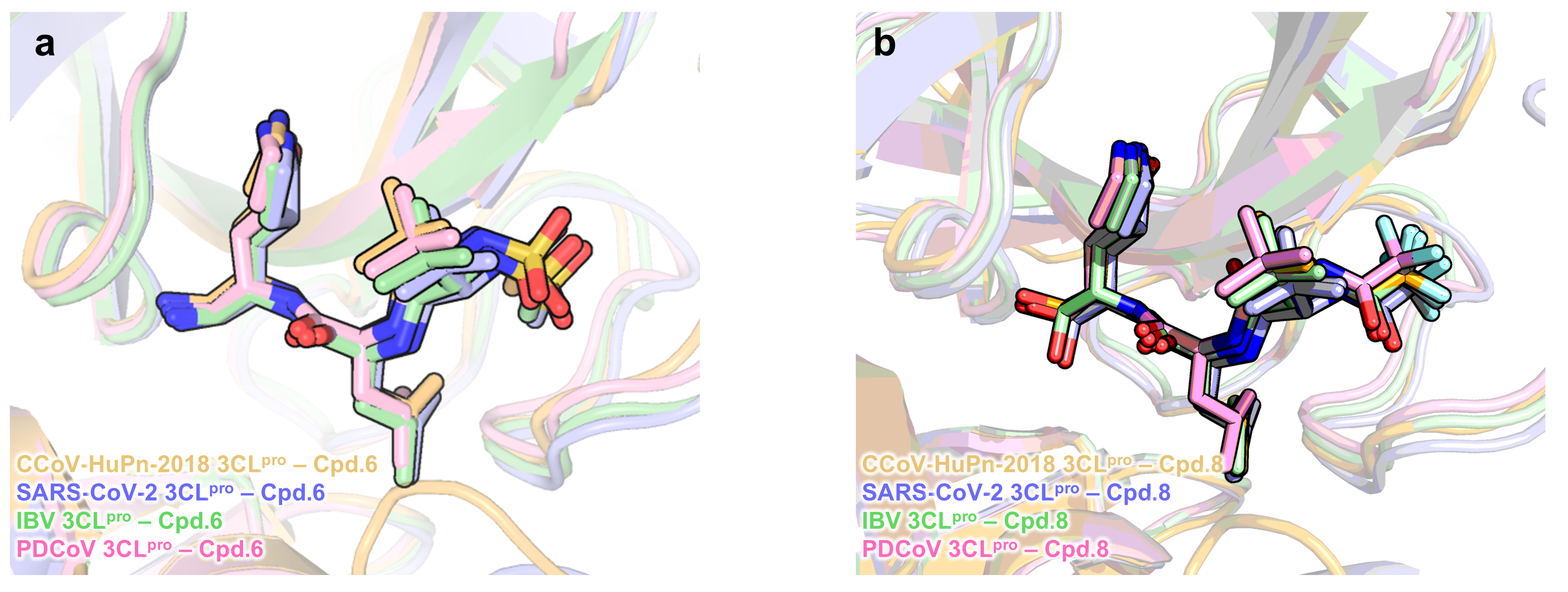


**Figure S6. Superimposed binding modes of compound 6 (a) and 8 (b) with four different 3CL^pro^s revealed by the determined co-crystal structures.** The compound **6** and **8** are shown as sticks.


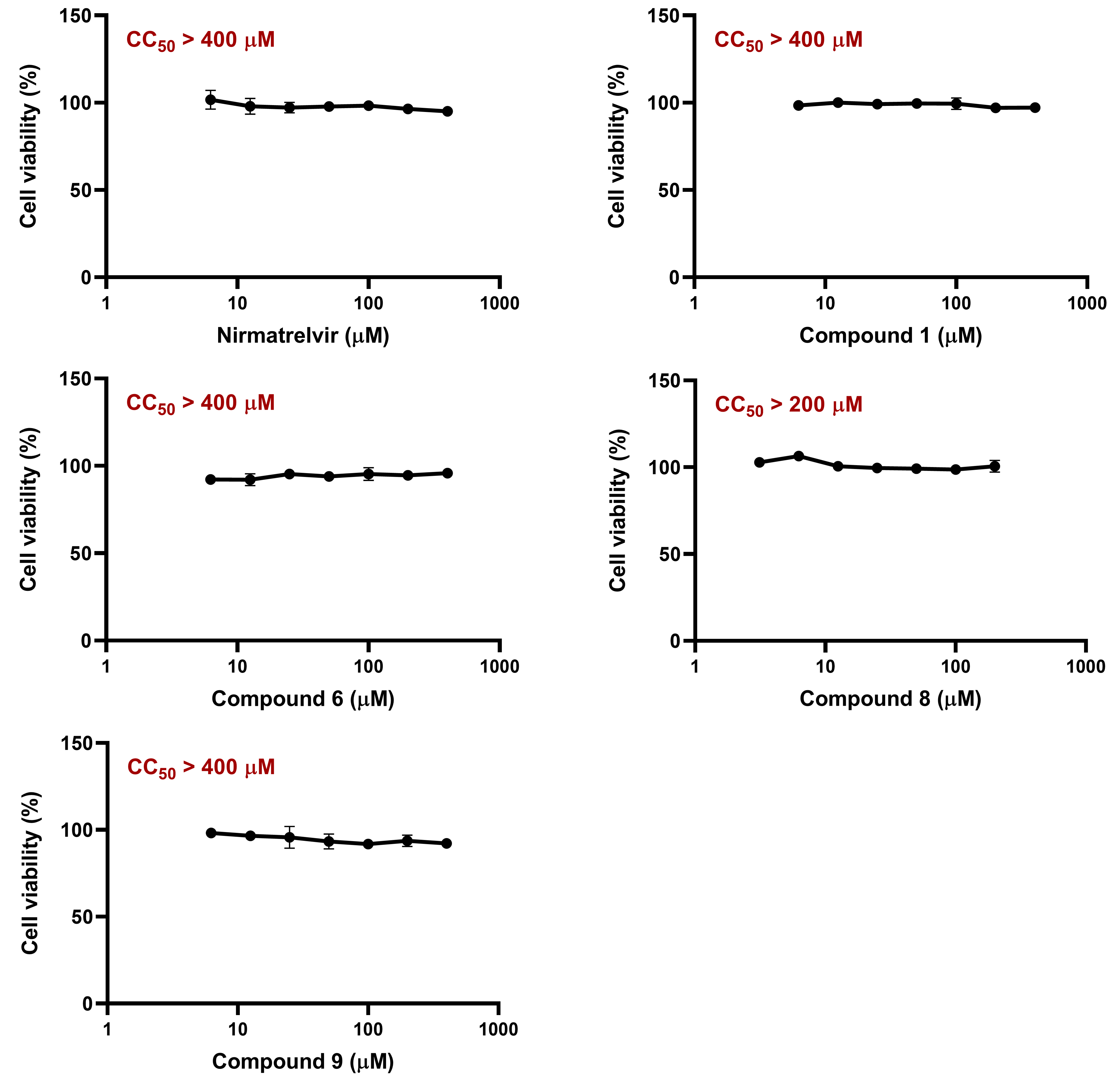


**Figure S7. Cytotoxicities of nirmatrelvir, compounds 1, 6, 8, and 9 in HEK293T cells.** Error bars represent mean ± SD. Experiments were performed in triplicate.


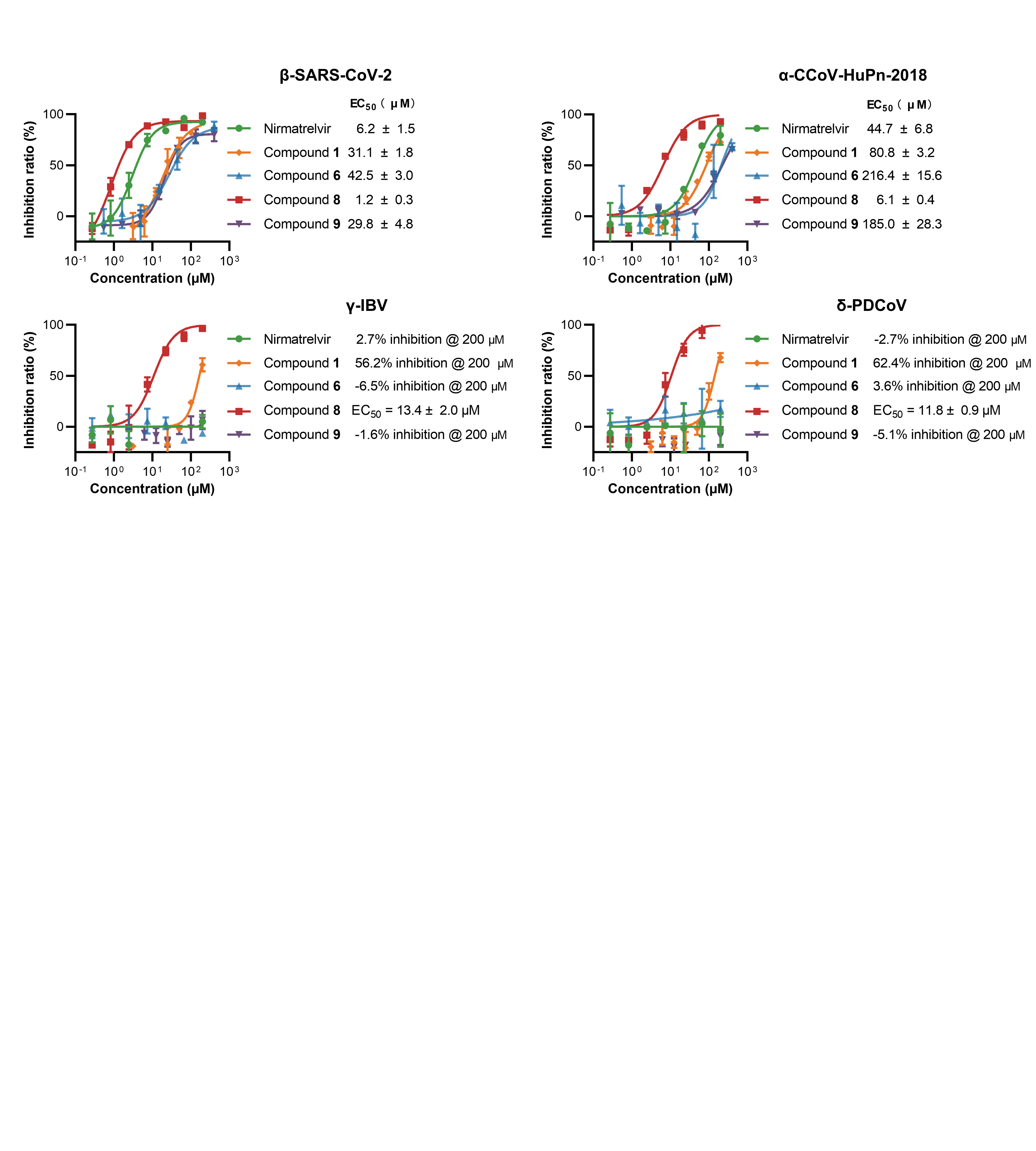


**Figure S8. Representative inhibitory profiles for the compounds against CoV 3CL^pro^s determined by the ODD-luciferase pseudovirus assay.** The data are plotted as the mean ± SD. Three independent experiments were performed.


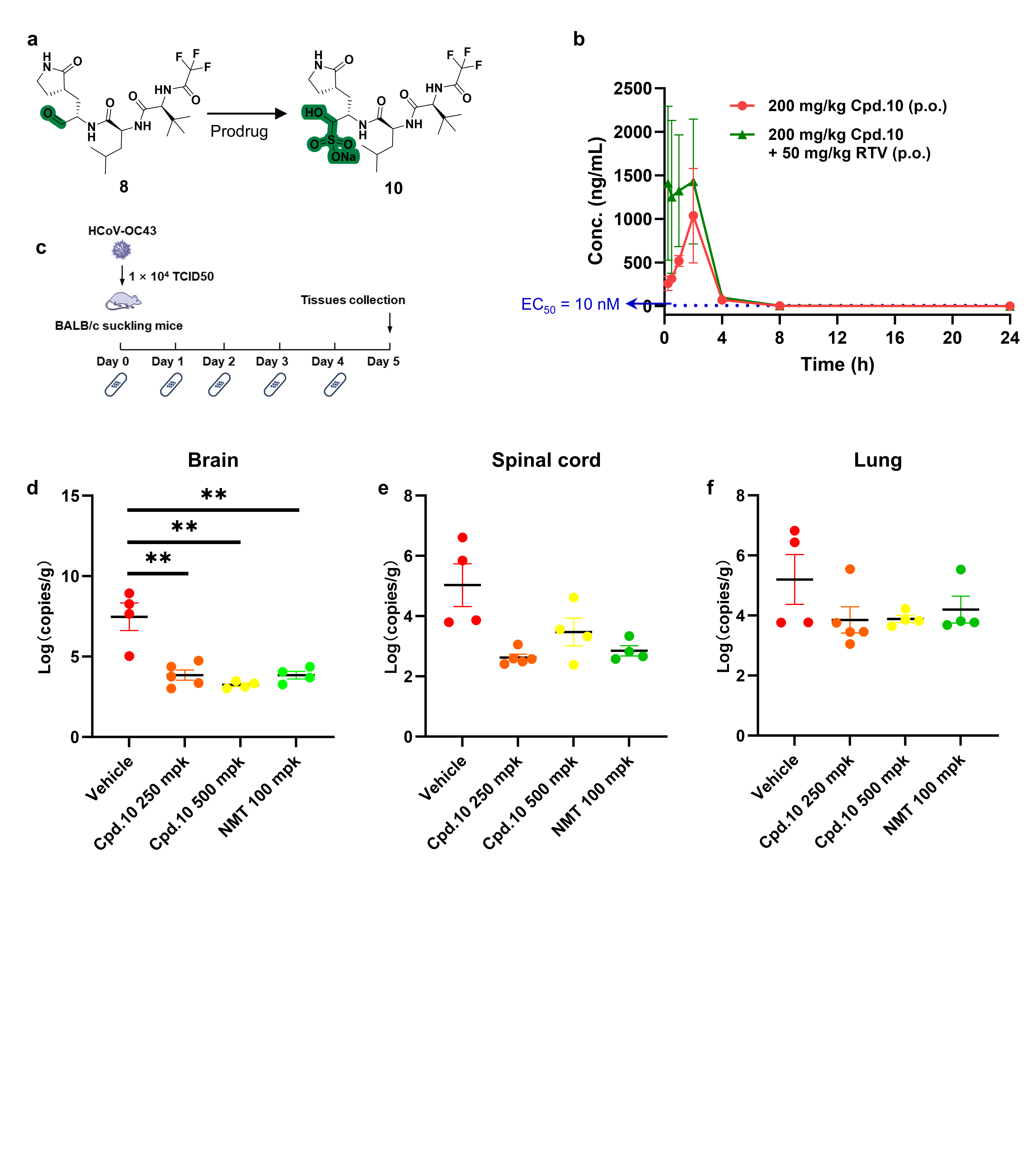


**Figure S9. Pharmacokinetic profiling and *in vivo* antiviral evaluation of compound 10 against HCoV-OC43.** (a) Prodrug design of compound **8**, with the prodrug modification site highlighted in green. (b) Plasma concentration-time profiles of compound **10** following oral administration. Red circles: 200 mg/kg compound **10** (p.o.); green triangles: 200 mg/kg compound 10 co-administered with 50 mg/kg ritonavir (p.o.). (c) Schematic of the in vivo experiment protocol: oral administration once daily for 5 days. (d–f) Viral RNA copies detected in the brain (d), spinal cord (e), and lung (f) tissues of HCoV-OC43-infected suckling mice, collected on day 5 post-treatment with vehicle, compound **10**, or nirmatrelvir. Nirmatrelvir (100 mg/kg), co-administered with ritonavir (50 mg/kg), served as a positive control. Data are shown as mean ± SEM; statistical significance was determined by two-tailed unpaired t-test. ^**^P < 0.01. The initial group size was five mice, with reductions due to filial cannibalism.

**Figure S10. Procedure and synthetic scheme for compound 1.**

Reagents and conditions: (a) Isobutyl chloroformate, *N*-methyl morphofine (NMM), tetrahydrofuran (THF), -20 °C; (b) Pd/C, H_2_, methanol, rt; (c) (S)-2-Amino-3-((S)-2-oxopyrrolidin-3-yl) propanenitrile hydrochloride, isobutyl chloroformate, NMM, THF, -20 °C.

**Figure S11. Procedure and synthetic scheme for compounds 2-4.**

Reagents and conditions: (a) LiOH, H_2_O, tetrahydrofuran (THF), methanol, 0 °C – rt; (b) (S)-2-Amino-3-((S)-2-oxopyrrolidin-3-yl)propanenitrile hydrochloride, 2-hydroxypyridine 1-oxide (HOPO), 1-[3-(dimethylamino)propyl]-3-ethylcarbodiimide hydrochloride (EDCI), *N*,*N*-diisopropylethyl amine (DIPEA), butan-2-one (MEK), 0 °C – rt; (c) 4 M HCl in dioxane, 1,4-dioxane, rt; (d) Trifluoromethanesulfonic anhydride, triethylamine (TEA), dichloromethane (DCM), 0 °C – rt; (e) Methanesulfonic anhydride, TEA, DCM, 0 °C – rt; (f) Cyclopropanesulfonyl chloride, TEA, DCM, 0 °C – rt.

**Figure S12. Procedure and synthetic scheme for compounds 5 - 7.**

Reagents and conditions: (a) Isobutyl chloroformate, NMM, THF, -20 °C; (b) 4 M HCl in dioxane, DCM, rt; (c) Trifluoromethanesulfonic anhydride, TEA, DCM, -78 °C; (d) Pd/C, H_2_, methanol, rt; (e) (S)-2-Amino-3-((S)-2-oxopyrrolidin-3-yl)propanenitrile hydrochloride, isobutyl chloroformate, NMM, THF, -20 °C; (f) *p*-Toluenesulfonic acid (TsOH), hexafluoroisopropanol (HFIP), 0 °C – rt; (g) Methanesulfonic anhydride, NMM, DCM, 0 °C – rt; (h) Cyclopropanesulfonyl chloride, NMM, DCM, 0 °C – rt.

**Figure S13. Procedures and synthetic scheme for compounds 8 - 10.**

Reagents and conditions: (a) Benzoyl chloride, pyridine, 0 °C – rt; (b) 4 M HCl in dioxane, DCM, rt; (c) Isobutyl chloroformate, NMM, THF, -20 °C; (c) Isobutyl chloroformate, NMM, THF, -20 °C; (d) K_2_CO_3_, MeOH, rt; (e) (Diacetoxyiodo)benzene, 2,2,6,6-tetramethyl-1-piperinedinyloxy (TEMPO), DCM, rt (f) Methanesulfonic anhydride, TEA, DCM, -78 °C; (g) Pd/C, H_2_, methanol, rt; (h) LiBH4, THF, 0 °C – rt; (i) Dess-Martin periodinane, NaHCO_3_, DCM, rt. (j) NaHSO3, EA, EtOH, 50 °C.


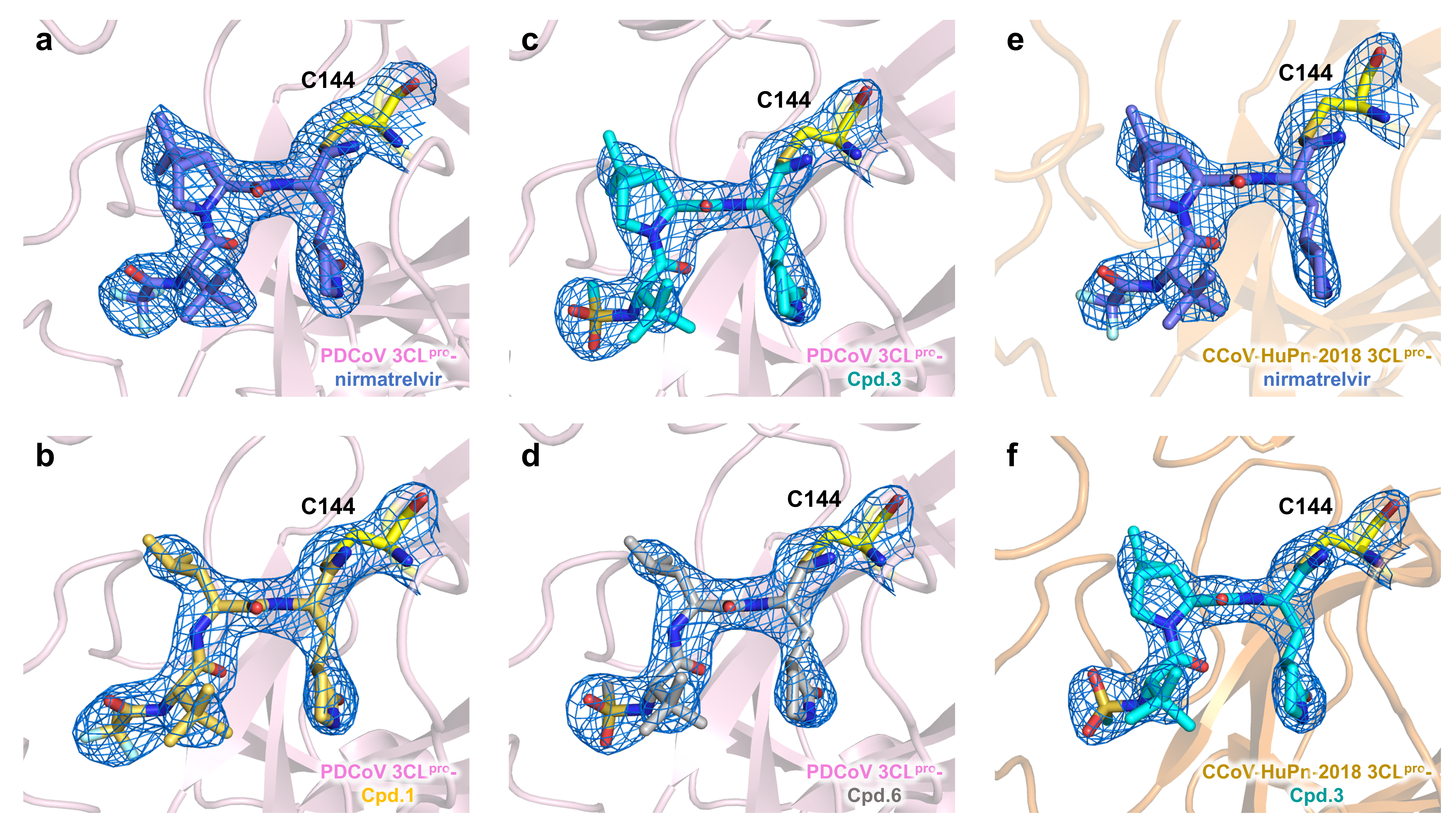


**Figure S14. 2*Fo-Fc* electron density maps contoured at 2.0 σ are shown for compounds and catalytic residue Cys144.** PDCoV 3CL^pro^ (a-d) and CCoV-HuPn-2018 3CL^pro^ (e-f) are shown as light pink and brown cartoon, respectively. Compounds and catalytic residue Cys144 are shown as sticks in different colors.

# S2: Supplementary Tables

**Table S1. Crystallography data collection and refinement statistics of 3CL^pro^s in complex with inhibitors.**

| **PDB ID** | **9X7D** | **9X7E** | **9X7F** |
| --- | --- | --- | --- |
| Protein | PDCoV 3CL^pro^ | CCoV-HuPn-2018 3CL^pro^ | PDCoV 3CL^pro^ |
| Ligand | nirmatrelvir | nirmatrelvir | **1** |
| Space Group | *P 61* | *C 1 2 1* | *P 61* |
| Cell Dimension: a (Å) | 122.521 | 159.988 | 123.577 |
| b (Å) | 122.521 | 126.453 | 123.577 |
| c (Å) | 292.153 | 159.802 | 291.312 |
| Wavelength (Å) | 0.97918 | 0.97918 | 0.97918 |
| Reflections (unique) | 108571 (11209) | 122447 (12792) | 73484 (7310) |
| Resolution Range (Å) | 30.46-2.28 | 38.67-2.37 | 40.45-2.64 |
| Highest-Resolution Shell (Å) | 2.36-2.28 | 2.46-2.37 | 2.73-2.64 |
| Redundancy | 10.2 (10.5) | 4.0 (4.3) | 18.2 (17.2) |
| I/σ (I) | 20.89 (2.42) | 8.10 (2.09) | 14.16 (2.39) |
| Highest-Resolution Shell CC_1/2_ | 0.849 | 0.885 | 0.792 |
| Completeness (%) | 96.36 (99.46) | 94.85 (99.73) | 99.72 (99.54) |
| Rwork/Rfree | 0.2082/0.2488 | 0.2628/0.3003 | 0.2034/0.2439 |
| **RMS Values** | | | |
| Bond length (Å) | 0.003 | 0.003 | 0.004 |
| Bond angle (°) | 0.56 | 0.62 | 0.60 |
| **Numbers of Non-hydrogen Atoms** | | | |
| Protein | 13713 | 15483 | 13487 |
| Inhibitor | 210 | 245 | 198 |
| Water Oxygen | 174 | 41 | 76 |
| Others | 0 | 0 | 0 |
| Clashscore | 3.08 | 4.55 | 4.29 |
| MolProbity Score | 1.24 | 1.58 | 1.45 |
| **B-factor (Å^2^)** | | | |
| Protein | 53.11 | 52.29 | 50.34 |
| Inhibitor | 51.34 | 50.40 | 48.77 |
| Water Oxygen | 46.60 | 47.22 | 38.98 |
| **Ramachandran plot** | | | |
| Favored (%) | 97.25 | 94.90 | 96.37 |
| Allowed (%) | 2.69 | 4.69 | 3.52 |
| Outliers (%) | 0.06 | 0.41 | 0.12 |

**Table S1. Crystallography data collection and refinement statistics of 3CL^pro^s in complex with inhibitors. (continued)**

| **PDB ID** | **9X7G** | **9X7I** | **9X7H** |
| --- | --- | --- | --- |
| Protein | PDCoV 3CL^pro^ | CCoV-HuPn-2018 3CL^pro^ | PDCoV 3CL^pro^ |
| Ligand | **3** | **3** | **6** |
| Space Group | *P 61* | *C 1 2 1* | *P 61* |
| Cell Dimension: a (Å) | 122.1 | 159.119 | 122.926 |
| b (Å) | 122.1 | 125.566 | 122.926 |
| c (Å) | 291.55 | 158.604 | 291.591 |
| Wavelength (Å) | 0.97856 | 0.97918 | 0.97918 |
| Reflections (unique) | 77895 (7777) | 150547 (14977) | 79835 (7915) |
| Resolution Range (Å) | 39.97-2.57 | 31.72-2.23 | 53.23-2.56 |
| Highest-Resolution Shell (Å) | 2.66-2.57 | 2.31-2.23 | 2.65-2.56 |
| Redundancy | 10.3 (10.5) | 6.4 (6.7) | 9.5 (9.8) |
| I/σ (I) | 11.60 (1.38) | 12.31 (2.27) | 18.22 (2.55) |
| Highest-Resolution Shell CC_1/2_ | 0.583 | 0.882 | 0.813 |
| Completeness (%) | 99.84 (99.70) | 99.20 (98.89) | 99.78 (99.57) |
| Rwork/Rfree | 0.2089/0.2577 | 0.2368/0.2804 | 0.1983/0.2439 |
| **RMS Values** | | | |
| Bond length (Å) | 0.003 | 0.003 | 0.003 |
| Bond angle (°) | 0.61 | 0.53 | 0.60 |
| **Numbers of Non-hydrogen Atoms** | | | |
| Protein | 13601 | 16989 | 13866 |
| Inhibitor | 198 | 264 | 186 |
| Water Oxygen | 84 | 154 | 112 |
| Others | 0 | 0 | 0 |
| Clashscore | 4.33 | 4.58 | 4.57 |
| MolProbity Score | 1.44 | 1.53 | 1.43 |
| **B-factor (Å^2^)** | | | |
| Protein | 57.42 | 52.54 | 56.44 |
| Inhibitor | 53.08 | 51.34 | 54.31 |
| Water Oxygen | 49.51 | 44.34 | 48.18 |
| **Ramachandran plot** | | | |
| Favored (%) | 96.57 | 95.66 | 96.80 |
| Allowed (%) | 3.14 | 4.21 | 3.14 |
| Outliers (%) | 0.29 | 0.13 | 0.06 |

**Table S1. Crystallography data collection and refinement statistics of 3CL^pro^s in complex with inhibitors. (continued)**

| **PDB ID** | **9IR8** | **9IR9** | **9IRA** |
| --- | --- | --- | --- |
| Protein | CCoV-HuPn-2018 3CL^pro^ | SARS-CoV-2 3CL^pro^ | IBV 3CL^pro^ |
| Ligand | **6** | **6** | **6** |
| Space Group | *P 21 21 2* | *P 21 21 2* | *P 1 21 1* |
| Cell Dimension: a (Å) | 112.102 | 45.49 | 55.43 |
| b (Å) | 127.579 | 63.43 | 95.08 |
| c (Å) | 111.908 | 105.60 | 71.50 |
| Wavelength (Å) | 0.97918 | 0.97918 | 0.97910 |
| Reflections (unique) | 90118 (9034) | 50327 (7240) | 80389 (8008) |
| Resolution Range (Å) | 111.9-2.12 | 41.78-1.49 | 34.81-1.66 |
| Highest-Resolution Shell (Å) | 2.20-2.12 | 1.55-1.49 | 1.72-1.66 |
| Redundancy | 10.8 (8.9) | 6.5 (4.8) | 6.5 (5.3) |
| I/σ (I) | 21.97 (2.38) | 13.7 (0.9) | 15.62 (1.60) |
| Highest-Resolution Shell CC_1/2_ | 0.931 | 0.459 | 0.636 |
| Completeness (%) | 98.25 (99.37) | 99.58 (96.27) | 99.72 (99.68) |
| Rwork/Rfree | 0.2427/0.2729 | 0.1968/0.2329 | 0.2239/0.2534 |
| **RMS Values** | | | |
| Bond length (Å) | 0.003 | 0.012 | 0.006 |
| Bond angle (°) | 0.55 | 1.33 | 0.83 |
| **Numbers of Non-hydrogen Atoms** | | | |
| Protein | 8649 | 2326 | 4545 |
| Inhibitor | 124 | 31 | 62 |
| Water Oxygen | 133 | 198 | 259 |
| Others | 0 | 0 | 0 |
| Clashscore | 4.06 | 3.69 | 2.22 |
| MolProbity Score | 1.45 | 1.16 | 1.00 |
| **B-factor (Å^2^)** | | | |
| Protein | 53.74 | 27.6 | 33.39 |
| Inhibitor | 49.62 | 21.5 | 31.02 |
| Water Oxygen | 47.52 | 37.1 | 40.50 |
| **Ramachandran plot** | | | |
| Favored (%) | 96.14 | 98.68 | 98.99 |
| Allowed (%) | 3.86 | 1.32 | 0.84 |
| Outliers (%) | 0.00 | 0.00 | 0.17 |

**Table S1. Crystallography data collection and refinement statistics of 3CL^pro^s in complex with inhibitors. (continued)**

| **PDB ID** | **9UOP** | **9UOQ** | **9UOR** |
| --- | --- | --- | --- |
| Protein | CCoV-HuPn-2018 3CL^pro^ | SARS-CoV-2 3CL^pro^ | IBV 3CL^pro^ |
| Ligand | **8** | **8** | **8** |
| Space Group | *P 21 21 2* | *P 21 21 2* | *P 1 21 1* |
| Cell Dimension: a (Å) | 112.26 | 45.71 | 55.30 |
| b (Å) | 127.53 | 63.58 | 94.69 |
| c (Å) | 111.199 | 105.64 | 71.45 |
| Wavelength (Å) | 0.97918 | 0.97918 | 0.97918 |
| Reflections (unique) | 206465 (29886) | 25383 (2374) | 89570 (8945) |
| Resolution Range (Å) | 22.20-1.61 | 105.64-1.89 | 34.33-1.60 |
| Highest-Resolution Shell (Å) | 1.70-1.61 | 1.99-1.89 | 1.62-1.60 |
| Redundancy | 13.4 (12.9) | 11.3 (9.1) | 6.4 (5.2) |
| I/σ (I) | 11.1 (0.8) | 10.28 (0.98) | 11.33 (1.66) |
| Highest-Resolution Shell CC_1/2_ | 0.458 | 0.515 | 0.637 |
| Completeness (%) | 100.0 (100.0) | 99.35 (94.87) | 99.80 (99.68) |
| Rwork/Rfree | 0.2005/0.2302 | 0.2154/0.2576 | 0.2019/0.2283 |
| **RMS Values** | | | |
| Bond length (Å) | 0.014 | 0.004 | 0.014 |
| Bond angle (°) | 1.26 | 0.82 | 1.34 |
| **Numbers of Non-hydrogen Atoms** | | | |
| Protein | 9119 | 2331 | 4565 |
| Inhibitor | 264 | 66 | 66 |
| Water Oxygen | 798 | 131 | 419 |
| Others | 0 | 0 | 0 |
| Clashscore | 2.46 | 2.79 | 1.88 |
| MolProbity Score | 1.05 | 1.07 | 0.95 |
| **B-factor (Å^2^)** | | | |
| Protein | 30.22 | 31.37 | 22.74 |
| Inhibitor | 27.18 | 26.96 | 19.32 |
| Water Oxygen | 37.03 | 35.18 | 30.16 |
| **Ramachandran plot** | | | |
| Favored (%) | 97.89 | 98.68 | 99.00 |
| Allowed (%) | 2.11 | 1.32 | 0.83 |
| Outliers (%) | 0.00 | 0.00 | 0.17 |

**Table S1. Crystallography data collection and refinement statistics of 3CL^pro^s in complex with inhibitors. (continued)**

| **PDB ID** | **9UOS** |
| --- | --- |
| Protein | PDCoV 3CL^pro^ |
| Ligand | **8** |
| Space Group | *P 61* |
| Cell Dimension: a (Å) | 122.82 |
| b (Å) | 122.82 |
| c (Å) | 290.91 |
| Wavelength (Å) | 0.97918 |
| Reflections (unique) | 65498 (6128) |
| Resolution Range (Å) | 61.41-2.73 |
| Highest-Resolution Shell (Å) | 2.77-2.73 |
| Redundancy | 9.8 (9.9) |
| I/σ (I) | 8.14 (0.94) |
| Highest-Resolution Shell CC_1/2_ | 0.404 |
| Completeness (%) | 99.00 (94.28) |
| Rwork/Rfree | 0.2233/0.2594 |
| **RMS Values** | |
| Bond length (Å) | 0.004 |
| Bond angle (°) | 0.593 |
| **Numbers of Non-hydrogen Atoms** | |
| Protein | 13558 |
| Inhibitor | 198 |
| Water Oxygen | 22 |
| Others | 0 |
| Clashscore | 5.29 |
| MolProbity Score | 1.56 |
| **B-factor (Å^2^)** | |
| Protein | 65.19 |
| Inhibitor | 62.69 |
| Water Oxygen | 50.14 |
| **Ramachandran plot** | |
| Favored (%) | 95.94 |
| Allowed (%) | 4.68 |
| Outliers (%) | 0.17 |

**Table S2. Thermodynamic parameters of compounds binding to 3CL^pro^s.**

| **Target** | **Compounds** | ***K*_d_ (M)** | **Δ*G* (kJ/mol)** | **Δ*H* (kJ/mol)** | **-*T*Δ*S* (kJ/mol)** |
| --- | --- | --- | --- | --- | --- |
| SARS-CoV-2 3CL^pro^ | Nirmatrelvir | (6.20 ± 0.58)*10^-7^ | -36.03 ± 0.24 | -25.71 ± 0.35 | -10.32 ± 0.30 |
| PDCoV 3CL^pro^ | Nirmatrelvir | (1.50 ± 0.26)*10^-5^ | -27.10 ± 0.41 | -12.11 ± 2.54 | -14.99 ± 2.95 |
| PDCoV 3CL^pro^ | 1 | (9.62 ± 0.81)*10^-7^ | -33.78 ± 0.21 | -54.41 ± 0.98 | 20.63 ± 1.18 |
| PDCoV 3CL^pro^ | 3 | (1.83 ± 0.36)*10^-6^ | -32.26 ± 0.53 | -40.58 ± 1.78 | 8.32 ± 1.75 |
| PDCoV 3CL^pro^ | 6 | (9.91 ± 0.97)*10^-7^ | -33.71 ± 0.25 | -40.17 ± 0.64 | 6.46 ± 0.88 |
| CCoV 3CL^pro^ | Nirmatrelvir | (3.69 ± 1.46)*10^-6^ | -30.71 ± 1.08 | -11.82 ± 0.62 | -18.89 ± 1.22 |
| CCoV 3CL^pro^ | 1 | (5.51 ± 1.59)*10^-6^ | -29.60 ± 0.65 | -20.09 ± 0.32 | -9.51 ± 0.92 |
| CCoV 3CL^pro^ | 3 | (1.21 ± 0.36)*10^-6^ | -33.31 ± 0.68 | -21.97 ± 0.27 | -11.34 ± 0.78 |
| CCoV 3CL^pro^ | 6 | (9.50 ± 1.22)*10^-7^ | -33.82 ± 0.33 | -32.13 ± 0.20 | -1.69 ± 0.39 |

**Table S3. The determined IC_50_ values of nirmatrelvir and compound 8 against 3CL^pro^s from CoVs covering 26 subgenera.**

| **Genus** | **Subgenus（26）** | **Exemplar CoVs name** | **IC_50_ ^nirmatrelvir^ (nM)** | **IC_50_ ^compound 8^ (nM)** | **IC_50_ ^nirmatrelvir^/ IC_50_ ^compound 8^** |
| --- | --- | --- | --- | --- | --- |
| α | *Amalacovirus* | BtCoV-AMA-L-F | 46 | 19 | 2.4 |
|  | *Colacovirus* | BtCoV-CDPHE15 | 334 | 79 | 4.2 |
|  | *Decacovirus* | BtRf-AlphaCoV | 195 | 40 | 4.9 |
|  | *Duvinacovirus* | HCoV-229E* | 48 | 29 | 1.7 |
|  | *Luchacovirus* | LRNV | 399 | 146 | 2.7 |
|  | *Minacovirus* | MCoV | 254 | 60 | 4.2 |
|  | *Minunacovirus* | Mi-BatCoV_1A | 212 | 97 | 2.2 |
|  | *Myotacovirus* | BtMr-AlphaCoV | 556 | 86 | 6.5 |
|  | *Nyctacovirus* | BtNv-AlphaCoV | 151 | 70 | 2.2 |
|  | *Pedacovirus* | PEDV | 65 | 35 | 1.9 |
|  | *Rhinacovirus* | Rh-BatCoV_HKU2 | 207 | 44 | 4.7 |
|  | *Setracovirus* | HCoV-NL63^*^ | 132 | 53 | 2.5 |
|  | *Soracovirus* | Sa-CoV_T14 | 40 | 61 | 0.7 |
|  | *Sunacovirus* | Sm-CoV_X74 | 221 | 44 | 5.0 |
|  | *Tegacovirus* | TGEV | 263 | 59 | 4.5 |
|  |  | FIPV | 101 | 41 | 2.5 |
|  |  | CCoV-HuPn-2018^*^ | 313 | 61 | 5.1 |
| β | *Embecovirus* | HCoV-HKU1^*^ | 31 | 28 | 1.1 |
|  |  | HCoV-OC43^*^ | 30 | 28 | 1.1 |
|  | *Hibecovirus* | Bat_Hp-BetaCoV | 31 | 28 | 1.1 |
|  | *Merbecovirus* | MERS-CoV^*^ | 133 | 106 | 1.3 |
|  |  | BtCoV-HKU4 | 39 | 22 | 1.8 |
|  |  | BtCoV-HKU5 | 34 | 19 | 1.8 |
|  | *Nobecovirus* | BtCoV-HKU9 | 37 | 24 | 1.5 |
|  | *Sarbecovirus* | SARS-CoV^*^ | 70 | 38 | 1.8 |
|  |  | SARS-CoV-2^*^ | 26 | 21 | 1.2 |
| γ | *Brangacovirus* | BcanCoV_CB17 | 106 | 84 | 1.3 |
|  | *Cegacovirus* | BWCoV | 80 | 31 | 2.6 |
|  | *Igacovirus* | IBV | 209 | 22 | 9.5 |
| δ | *Andecovirus* | WiCoV_HKU20 | 55 | 26 | 2.1 |
|  | *Buldecovirus* | PDCoV^*^ | 1350 | 81 | 16.7 |
|  |  | Sp-CoV_HKU17 | 840 | 47 | 17.9 |
|  | *Herdecovirus* | NHCoV_HKU19 | 52 | 24 | 2.2 |

^*^ Human CoVs

**Table S4. Inhibitory activities of compound 8 against other proteases**

| **Proteases** | **IC_50_ (nM)** |
| --- | --- |
| SARS-CoV-2 PL^pro^ | > 50000 |
| Cathepsin B | 208 |
| Cathepsin L | 12 |
| Chymotrypsin | > 20000 |
| Caspase-6 | > 50000 |
| Cathepsin G | > 20000 |

**Table S5. The determined IC_50_ values of nirmatrelvir and compound 8 against SARS-CoV-2 3CL^pro^ and its nirmatrelvir-resistant mutants.**

| **3CL^pro^s** | **IC_50_ ^nirmatrelvir^ (nM)** | **IC_50_ ^compound 8^ (nM)** | **IC_50_ ^nirmatrelvir^/ IC_50_ ^compound 8^** |
| --- | --- | --- | --- |
| **WT** | 26 | 21 | 1.2 |
| **S144A** | 117 | 41 | 2.9 |
| **E166V** | 16190 | 294 | 55.1 |
| **L50F** | 42 | 47 | 0.9 |
| **L167F** | 184 | 89 | 2.1 |
| **△P168** | 65 | 52 | 1.3 |
| **A173V** | 66 | 51 | 1.3 |

# S3: Synthetic Procedures and Spectral Data for Synthetic Compounds

Abbreviations

| DCM: | dichloromethane |
| --- | --- |
| DIPEA: | *N*,*N*-diisopropylethyl amine |
| EA: | ethyl acetate |
| EDCI: | 1-[3-(dimethylamino)propyl]-3-ethylcarbodiimide hydrochloride |
| HFIP: | hexafluoroisopropanol |
| HOPO: | 2-hydroxypyridine 1-oxide |
| MEK: | butan-2-one |
| NMM: | *N*-methyl morphofine |
| PE: | petroleum ether |
| TEA： | triethylamine |
| TEMPO | 2,2,6,6-tetramethyl-1-piperinedinyloxy |
| TsOH: | *p*-toluenesulfonic acid |

Synthesis of **1**

***Benzyl ((S)-3,3-dimethyl-2-(2,2,2-trifluoroacetamido)butanoyl)-L-******leucinate (T1).*** Isobutyl chloroformate (174 μL, 1.27 mmol) and NMM (146 μL, 1.32 mmol) were added to a -20 °C solution of (S)-3,3-dimethyl-2-(2,2,2-trifluoroacetamido)butanoic acid (300 mg, 1.32 mmol) in THF (5 mL) dropwise. The solution was stirred at -20 °C for 30 min. Then a solution of benzyl *L*-leucinate hydrochloride (306 mg, 1.19 mmol) and NMM (146 μL, 1.32 mmol) in THF (2 mL) was added, and stirring was continued for 5 h. The resulting mixture was concentrated, extracted with ethyl acetate, washed with saturated aqueous sodium chloride solution, dried over sodium sulfate, and concentrated. Silica gel column chromatography (Gradient: 0% to 10% EA in PE) provided **T1** as a white solid. Yield: 220 mg, 39%. ^1^H NMR (500 MHz, Chloroform-*d*) δ 7.41–7.31 (m, 5H), 7.07 (d, *J* = 9.2 Hz, 1H), 5.96 (d, *J* = 8.0 Hz, 1H), 5.17 (q, *J* = 12.2 Hz, 2H), 4.65 (td, *J* = 8.4, 5.3 Hz, 1H), 4.26 (d, *J* = 9.2 Hz, 1H), 1.72–1.65 (m, 1H), 1.64–1.51 (m, 2H), 1.00 (s, 9H), 0.92 (dd, *J* = 6.4, 3.9 Hz, 6H).

***((S)-3,3-Dimethyl-2-(2,2,2-trifluoroacetamido)butanoyl)-L-leucine (T2).*** 10% Pd/C (22 mg, 10wt% of the substrate) was added to a solution of **T1** (220 mg, 0.51 mmol) in MeOH (3 mL). Then the reaction was stirred at room temperature under a hydrogen atmosphere (balloon) for 12 h. The mixture was filtrated, and the filtrate was concentrated to afford **T2** as a white solid, which was used in the next step without further purification. Yield: 154 mg, 89%. ^1^H NMR (500 MHz, Methanol-*d*_4_) δ 4.49–4.44 (m, 2H), 1.76–1.70 (m, 1H), 1.68–1.63 (m, 2H), 1.07 (s, 9H), 0.96 (dd, *J* = 23.5, 6.4 Hz, 6H).

***(S)-N-((S)-1-Cyano-2-((S)-2-oxopyrrolidin-3-yl)ethyl)-2-((S)-3,3-dimethyl-2-(2,2,2-trifluoroacetamido)butanamido)-4-methylpentanamide (1).*** Isobutyl chloroformate (40 μL, 0.28 mmol) and NMM (34 μL, 0.29 mmol) were added to a -20 °C solution of **T2** (100 mg, 0.29 mmol) in THF (5 mL) dropwise. The reaction mixture was stirred at -20 °C for 30 min. A solution of (S)-2-amino-3-((S)-2-oxopyrrolidin-3-yl)propanenitrile hydrochloride (57 mg, 0.29 mmol) and NMM (34 μL, 0.29 mmol) in THF (2 mL) was added, and stirring was continued for 16 h. The resulting mixture was concentrated, extracted with ethyl acetate, washed with aqueous sodium chloride solution, dried over sodium sulfate, and concentrated. Silica gel column chromatography (Gradient: 0% to 3.3% MeOH in DCM) provided **1** as a white solid with a small amount of byproduct. Yield, 100 mg, 72%. Further purification via reversed-phase HPLC (Column: Waters Sunfire C18, 19 x 150 mm, 5 µm; Mobile phase A: water containing 0.1% trifluoroacetic acid; Mobile phase B: acetonitrile containing 0.1% trifluoroacetic acid; Gradient: 10% to 50% B over 15 minutes, then 50% to 65% B over 10 min, then 65% to 100% B for 7 min; Flow rate: 10 mL/min) afforded **1** as trifluoroacetic acid salt form. HPLC purity: 97.1% (RT = 15.23 min). ^1^H NMR (500 MHz, Chloroform-*d*) δ 8.30 (d, *J* = 6.7 Hz, 1H), 7.31 (d, *J* = 9.2 Hz, 1H), 7.02–6.94 (m, 2H), 4.89 (dt, *J* = 10.2, 6.3 Hz, 1H), 4.46 (td, *J* = 7.9, 5.7 Hz, 1H), 4.42 (d, *J* = 9.3 Hz, 1H), 3.51–3.38 (m, 2H), 2.67–2.58 (m, 1H), 2.49–2.41 (m, 1H), 2.36–2.27 (m, 1H), 2.10–2.02 (m, 1H), 1.97–1.86 (m, 1H), 1.71–1.54 (m, 3H), 1.01 (s, 9H), 0.93 (dd, *J* = 10.3, 6.0 Hz, 6H). ^13^C NMR (126 MHz, Chloroform-*d*) δ 180.49, 172.32, 169.41, 157.35 (q, *J* = 37.8 Hz), 117.75, 116.20 (q, *J* = 287.4 Hz), 61.06, 52.18, 41.30, 41.15, 39.55, 38.35, 35.20, 33.51, 27.96, 26.41, 24.80, 22.56, 22.01. NMR (471 MHz, Chloroform-*d*) δ -75.52, -76.04 (trifluoroacetic acid as counterion). ESI-HRMS Calcd for C_21_H_33_F_3_N_5_O_4_ [M+H]^+^: 476.2479, found 476.2478.

Synthesis of **2**

***(1R,2S,5S)-3-((S)-2-((Tert-butoxycarbonyl)amino)-3,3-dimethylbutanoyl)-6,6-dimethyl-3-azabicyclo[3.1.0]hexane-2-carboxylic acid (T3).*** To a 0 °C solution of methyl (1R,2S,5S)-3-((S)-2-((tert-butoxycarbonyl)amino)-3,3-dimethylbutanoyl)-6,6-dimethyl-3-azabicyclo[3.1.0]hexane-2-carboxylate (1.00 g, 2.61 mmol) in THF and MeOH (5 mL + 5 mL) was added a solution of LiOH (3.92 mL, 3.92 mmol, 1M in H_2_O) dropwise. The reaction mixture was stirred at 0 °C for 1 h and stirred at room temperature for another 1 h. The resulting mixture was then adjusted to a pH of 3 to 4 by addition of 1 M HCl, whereupon it was concentrated, extracted with ethyl acetate, washed with saturated aqueous sodium chloride solution, and dried over sodium sulfate. The organic phase was concentrated *in vacuo*, and used in the next step without further purification. Yield: 950 mg, 99%. ^1^H NMR (500 MHz, Methanol-*d*_4_) δ 4.35 (s, 1H), 4.24–4.20 (m, 1H), 4.05–4.01 (m, 1H), 3.96–3.90 (m, 1H), 1.60–1.56 (m, 1H), 1.52–1.49 (m, 1H), 1.44 (s, 9H), 1.09 (s, 3H), 1.04 (s, 9H), 0.95 (s, 3H).

***Tert-butyl ((S)-1-((1R,2S,5S)-2-(((S)-1-cyano-2-((S)-2-oxopyrrolidin-3-yl)ethyl)carbamoyl)-6,6-dimethyl-3-azabicyclo[3.1.0]hexan-3-yl)-3,3-dimethyl-1-oxobutan-2-yl)carbamate (T4).*** 2-Hydroxypyridine 1-oxide (59 mg, 0.54 mmol) was added to a solution of **T3** (787 mg, 2.13 mmol) and (S)-2-amino-3-((S)-2-oxopyrrolidin-3-yl)propanenitrile hydrochloride (405 mg, 2.13 mol) in butan-2-one (20 mL), and the mixture was cooled to 0 °C. DIPEA (1.06 mL, 6.41 mmol) was then added, followed by the addition of EDCI (490 mg, 2.56 mmol). The reaction mixture was stirred at 25 °C for 16 h, whereupon it was concentrated, extracted with EA, and washed with 1M HCl, 5% sodium bicarbonate, and saturated aqueous sodium chloride solution. The separated organic layer was dried over sodium sulfate, concentrated, and purified by silica gel column chromatography (Gradient: 0% to 3.3% MeOH in DCM) to afford **T4** as a white solid. Yield: 995 mg, 93%. ^1^H NMR (500 MHz, Chloroform-*d*) δ 8.11 (d, *J* = 7.3 Hz, 1H), 6.16 (s, 1H), 5.17 (d, *J* = 10.2 Hz, 1H), 5.01–4.89 (m, 1H), 4.30 (s, 1H), 4.21 (d, *J* = 10.2 Hz, 1H), 3.99 (d, *J* = 10.3 Hz, 1H), 3.92–3.86 (m, 1H), 3.37–3.28 (m, 2H), 2.64–2.49 (m, 1H), 2.46–2.28 (m, 2H), 2.00–1.90 (m, 2H), 1.87–1.79 (m, 1H), 1.40 (s, 9H), 1.06 (s, 3H), 0.98 (s, 9H), 0.89 (s, 3H).

***(1R,2S,5S)-N-((S)-1-Cyano-2-((S)-2-oxopyrrolidin-3-yl)ethyl)-3-((S)-3,3-dimethyl-2-((trifluoromethyl)sulfonamido)butanoyl)-6,6-dimethyl-3-azabicyclo[3.1.0]hexane-2-carboxamide (2).*** A solution of HCl in 1,4-dioxane (4 M; 262 μL, 1.05 mmol) was added to a solution of **T4** (88 mg, 0.17 mmol) in DCM (0.5 mL), and the reaction mixture was stirred at room temperature for 2 h. Then the reaction mixture was concentrated, dissolved in DCM (4 mL), and cooled to 0 °C. To this mixture was added TEA (60.5 µL, 0.434 mmol), followed by trifluoromethanesulfonic anhydride (53 mg, 0.19 mmol). The reaction mixture was warmed to room temperature slowly and stirred for 12 h. It was then extracted with DCM, washed with saturated aqueous sodium chloride solution, dried over sodium sulfate, and concentrated. Silica gel chromatography (Gradient: 0% to 3.3% MeOH in DCM) afforded **2** as a white solid with a small amount of byproduct. Yield: 18 mg, 18%. Further purification via reversed-phase HPLC (Column: Waters Sunfire C18, 19 x 150 mm, 5 µm; Mobile phase A: water containing 0.1% trifluoroacetic acid; Mobile phase B: acetonitrile containing 0.1% trifluoroacetic acid; Gradient: 10% to 50% B over 15 minutes, then 50% to 65% B over 10 min, then 65% to 100% B for 7 min; Flow rate: 10 mL/min) afforded **2** as trifluoroacetic acid salt form. HPLC purity: 98.7% (RT = 15.77 min). ^1^H NMR (500 MHz, DMSO-*d*_6_) δ 9.67 (d, *J* = 9.1 Hz, 1H), 9.09 (d, *J* = 8.7 Hz, 1H), 7.66 (s, 1H), 4.98 (ddd, *J* = 11.1, 8.6, 4.8 Hz, 1H), 4.18 (s, 1H), 3.92–3.87 (m, 2H), 3.50 (d, *J* = 10.4 Hz, 1H), 3.14 (td, *J* = 8.6, 1.7 Hz, 1H), 3.03 (td, *J* = 9.3, 7.1 Hz, 1H), 2.46–2.35 (m, 1H), 2.15 (ddd, *J* = 13.4, 11.1, 4.2 Hz, 1H), 2.11–2.02 (m, 1H), 1.78–1.66 (m, 2H), 1.59 (dd, *J* = 7.7, 5.6 Hz, 1H), 1.34 (d, *J* = 7.7 Hz, 1H), 1.04 (s, 3H), 0.99 (s, 9H), 0.89 (s, 3H). ^13^C NMR (126 MHz, DMSO-*d_6_*) δ 177.37, 170.51, 167.48, 119.52, 119.21 (q, *J* = 322.0 Hz), 62.52, 60.32, 47.44, 39.18, 37.58, 36.56, 35.42, 34.22, 30.35, 27.66, 26.71, 26.09, 25.57, 18.84, 12.28. ^19^F NMR (471 MHz, DMSO-*d_6_*) δ -74.76, -77.24 (trifluoroacetic acid as counterion). ESI-HRMS Calcd for C_22_H_33_F_3_N_5_O_5_S [M+H]^+^: 536.2149, found 536.2152.

Synthesis of **3**

***(1R,2S,5S)-N-((S)-1-Cyano-2-((S)-2-oxopyrrolidin-3-yl)ethyl)-3-((S)-3,3-dimethyl-2-(methylsulfonamido)butanoyl)-6,6-dimethyl-3-azabicyclo[3.1.0]hexane-2-carboxamide (3).*** Compound **3** was prepared according to the procedure of compound **2**. Trifluoroacetic acid salt form, yield: 23 mg, 25%. HPLC purity: 96.2% (RT = 13.50 min). ^1^H NMR (600 MHz, DMSO-*d*_6_) δ 9.04 (d, *J* = 8.7 Hz, 1H), 7.65 (s, 1H), 7.11 (d, *J* = 9.7 Hz, 1H), 4.99–4.94 (m, 1H), 4.17 (s, 1H), 3.85 (dd, *J* = 10.3, 5.6 Hz, 1H), 3.80 (d, *J* = 9.6 Hz, 1H), 3.68 (d, *J* = 10.4 Hz, 1H), 3.13 (t, *J* = 9.2 Hz, 1H), 3.03 (td, *J* = 9.4, 7.1 Hz, 1H), 2.84 (s, 3H), 2.46–2.39 (m, 1H), 2.16 (ddd, *J* = 13.4, 11.1, 4.3 Hz, 1H), 2.10–2.04 (m, 1H), 1.75–1.65 (m, 2H), 1.57 (dd, *J* = 7.7, 5.6 Hz, 1H), 1.30 (d, *J* = 7.7 Hz, 1H), 1.03 (s, 3H), 0.95 (s, 9H), 0.92 (s, 3H). ^13^C NMR (151 MHz, DMSO-*d*_6_) δ 177.39, 170.69, 169.22, 119.57, 60.31, 60.00, 47.41, 40.70, 39.11, 37.53, 36.53, 34.95, 34.12, 30.33, 27.60, 26.69, 26.15, 25.71, 18.83, 12.69. ESI-HRMS Calcd for C_22_H_36_N_5_O_5_S [M+H]^+^: 482.2432, found 482.2433.

Synthesis of **4**

***(1R,2S,5S)-N-((S)-1-Cyano-2-((S)-2-oxopyrrolidin-3-yl)ethyl)-3-((S)-2-(cyclopropanesulfonamido)-3,3-dimethylbutanoyl)-6,6-dimethyl-3-azabicyclo[3.1.0]hexane-2-carboxamide*** (**4**)**.** Compound **4** was prepared according to the procedure of compound **2**. Trifluoroacetic acid salt form, yield: 9 mg, 10%. HPLC purity: 96.9% (RT = 14.15 min). ^1^H NMR (500 MHz, DMSO-*d*_6_) δ 9.04 (d, *J* = 8.6 Hz, 1H), 7.65 (s, 1H), 7.09 (d, *J* = 9.5 Hz, 1H), 4.97 (ddd, *J* = 11.1, 8.6, 5.0 Hz, 1H), 4.16 (s, 1H), 3.85 (dd, *J* = 10.2, 5.6 Hz, 1H), 3.78 (d, *J* = 9.5 Hz, 1H), 3.68 (d, *J* = 10.3 Hz, 1H), 3.13 (t, *J* = 9.1 Hz, 1H), 3.03 (td, *J* = 9.4, 7.0 Hz, 1H), 2.49–2.38 (m, 2H), 2.16 (ddd, *J* = 13.5, 11.1, 4.3 Hz, 1H), 2.11–2.04 (m, 1H), 1.76–1.64 (m, 2H), 1.56 (dd, *J* = 7.7, 5.5 Hz, 1H), 1.30 (d, *J* = 7.7 Hz, 1H), 1.03 (s, 3H), 1.01–0.92 (m, 10H), 0.91 (s, 3H), 0.90–0.83 (m, 3H). ^13^C NMR (126 MHz, DMSO-*d*_6_) δ 177.50, 170.86, 169.39, 119.68, 60.63, 60.11, 47.44, 39.15, 37.65, 36.65, 35.17, 34.21, 30.57, 30.43, 27.73, 26.82, 26.34, 25.80, 18.96, 12.82, 5.60, 4.55. ESI-HRMS Calcd for C_24_H_38_N_5_O_5_S [M+H]^+^: 508.2588, found 508.2591.

Synthesis of **5**

***Benzyl ((S)-2-((tert-butoxycarbonyl)amino)-3,3-dimethylbutanoyl)-L-leucinate (T5).***

Isobutyl chloroformate (1.62 mL, 12.45 mmol) and NMM (2.85 mL, 25.94 mmol) were added to a -20 °C solution of (S)-3,3-dimethyl-2-(2,2,2-trifluoroacetamido)butanoic acid (3.00 g, 12.97 mmol) in THF (30 mL) dropwise. The solution was stirred at -20 °C for 30 min, whereupon benzyl *L*-leucinate hydrochloride (3.01 g, 11.67 mmol) was added, and stirring was continued for 20 h. The resulting mixture was concentrated, extracted with ethyl acetate. The separated organic layer was washed with 1 M HCl, 5% NaHCO_3_ and saturated aqueous sodium chloride solution. Then the organic phase was dried over sodium sulfate, and concentrated. Silica gel column chromatography (Gradient: 0% to 11% EA in PE) provided **T5** as a clear oil. Yield: 4.9 g, 87%. ^1^H NMR (500 MHz, Chloroform-*d*) δ 7.40–7.30 (m, 5H), 5.98 (d, *J* = 8.2 Hz, 1H), 5.24 (d, *J* = 9.6 Hz, 1H), 5.20–5.10 (m, 2H), 4.70–4.62 (m, 1H), 3.81 (d, *J* = 9.5 Hz, 1H), 1.69–1.62 (m, 2H), 1.58–1.52 (m, 1H), 1.43 (s, 9H), 0.98 (s, 9H), 0.91 (d, *J* = 6.1 Hz, 6H).

***Benzyl ((S)-3,3-dimethyl-2-((trifluoromethyl)sulfonamido)butanoyl)-L-leucinate (T6).*** A solution of HCl in 1,4-dioxane (4 M; 805 μL, 3.22 mmol) was added to a solution of **T5** (350 mg, 0.81 mmol) in DCM (1.00 mL), and the reaction mixture was stirred at room temperature for 2 h. Then the reaction mixture was concentrated, dissolved in DCM (4 mL), and cooled to -78 °C. To this mixture was added TEA (280 µL, 2.01 mmol), followed by trifluoromethanesulfonic anhydride (53 mg, 0.19 mmol). The reaction mixture was stirred at -78 °C for 12 h and quenched with ice water. It was then extracted with DCM, washed with saturated aqueous sodium chloride solution, dried over sodium sulfate, and concentrated. Silica gel chromatography (Gradient: 0% to 20% EA in PE) afforded **T6** as a white solid. Yield: 177 mg, 47%. ^1^H NMR (400 MHz, Chloroform-*d*) δ 7.40–7.31 (m, 5H), 6.14 (s, 1H), 5.97 (d, *J* = 8.3 Hz, 1H), 5.24–5.10 (m, 2H), 4.78–4.68 (m, 1H), 3.65 (s, 1H), 1.70–1.64 (m, 2H), 1.61–1.55 (m, 1H), 1.02 (s, 9H), 0.92 (dd, *J* = 6.2, 2.3 Hz, 6H).

***((S)-3,3-Dimethyl-2-((trifluoromethyl)sulfonamido)butanoyl)-L-leucine (T7).*** Compound **T7** was prepared according to the procedure of compound **T2**. Yield: 132 mg, 80%. ^1^H NMR (500 MHz, Methanol-*d*_4_) δ 4.46 (dd, *J* = 9.8, 5.3 Hz, 1H), 3.89 (s, 1H), 1.81–1.72 (m, 1H), 1.71–1.58 (m, 2H), 1.07 (s, 9H), 0.96 (dd, *J* = 23.1, 6.5 Hz, 6H).

***(S)-N-((S)-1-Cyano-2-((S)-2-oxopyrrolidin-3-yl)ethyl)-2-((S)-3,3-dimethyl-2-((trifluoromethyl)sulfonamido)butanamido)-4-methylpentanamide (5).*** Isobutyl chloroformate (44 μL, 0.34 mmol) and NMM (78 μL, 0.70 mmol) were added to a -20 °C solution of **T7** (132 mg, 0.35 mmol) in THF (5 mL) dropwise. The reaction mixture was stirred at -20 °C for 30 min. A solution of (S)-2-amino-3-((S)-2-oxopyrrolidin-3-yl)propanenitrile hydrochloride (67 mg, 0.35 mmol) in THF (2 mL) was added, and stirring was continued for 12 h. The resulting mixture was concentrated, extracted with ethyl acetate, washed with saturated aqueous sodium chloride solution, dried over sodium sulfate, and concentrated. Silica gel column chromatography (Gradient: 0% to 3.3% MeOH in DCM) provided **5** as a white solid. Yield: 120 mg, 67%. HPLC purity: 95.4% (RT = 14.92 min). ^1^H NMR (500 MHz, DMSO-*d*_6_) δ 9.63 (d, *J* = 9.8 Hz, 1H), 8.97 (d, *J* = 8.5 Hz, 1H), 8.33 (d, *J* = 6.7 Hz, 1H), 7.67 (s, 1H), 4.93 (ddd, *J* = 10.9, 8.4, 5.2 Hz, 1H), 4.20 (ddd, *J* = 10.2, 6.7, 5.1 Hz, 1H), 3.86 (d, *J* = 9.8 Hz, 1H), 3.18–3.10 (m, 1H), 3.07–3.01 (m, 1H), 2.40–2.31 (m, 1H), 2.15 (ddd, *J* = 13.4, 10.9, 4.4 Hz, 1H), 2.10–2.00 (m, 1H), 1.77–1.61 (m, 3H), 1.53 (ddd, *J* = 13.5, 10.2, 5.1 Hz, 1H), 1.38-1.29 (m, 1H), 0.96–0.90 (m, 12H), 0.85 (d, *J* = 6.6 Hz, 3H). ^13^C NMR (126 MHz, DMSO-*d*_6_) δ 177.42, 171.86, 168.42, 119.58, 119.42 (q, *J* = 323.0 Hz), 64.62, 51.21, 39.95, 39.25, 37.65, 36.72, 34.29, 33.75, 26.72, 26.30, 23.89, 22.86, 20.98. ^19^F NMR (471 MHz, DMSO-*d*_6_) δ -77.35. ESI-HRMS Calcd for C_20_H_33_F_3_N_5_O_5_S [M+H]^+^: 512.2149, found 512.2148.

Synthesis of **6**

***((S)-2-((Tert-butoxycarbonyl)amino)-3,3-dimethylbutanoyl)-L-leucine (T8).*** Compound **T8** was prepared according to the procedure of compound **T2**. Yield, 1.4 g, 88%. ^1^H NMR (500 MHz, Methanol-*d*_4_) δ 4.51–4.44 (m, 1H), 3.95 (s, 1H), 1.74 (ddd, *J* = 14.7, 12.8, 6.6 Hz, 1H), 1.69–1.61 (m, 2H), 1.46 (s, 9H), 1.02 (s, 9H), 0.98 (d, *J* = 6.5 Hz, 3H), 0.93 (d, *J* = 6.5 Hz, 3H).

***Tert-butyl ((S)-1-(((S)-1-(((S)-1-cyano-2-((S)-2-oxopyrrolidin-3-yl)ethyl)amino)-4-methyl-1-oxopentan-2-yl)amino)-3,3-dimethyl-1-oxobutan-2-yl)carbamate (T9).*** Isobutyl chloroformate (363 μL, 2.79 mmol) and NMM (320 μL, 2.90 mmol) were added to a -20 °C solution of **T8** (1 g, 2.90 mmol) in THF (20 mL) dropwise. The solution was stirred at -20 °C for 30 min. A solution of (S)-2-amino-3-((S)-2-oxopyrrolidin-3-yl)propanenitrile hydrochloride (500 mg, 2.61 mmol) and NMM (320 μL, 2.90 mmol) in THF (5 mL) was added, and stirring was continued for 16 h. Then the reaction mixture was concentrated, extracted with EA. The separated organic layer was washed with 1 M HCl, 5% sodium bicarbonate and saturated aqueous sodium chloride solution. Then the organic phase was concentrated *in vacuo* and the resulting solid was collected via filtration to afford **T9** as a white solid. Yield: 970 mg, 70%. ^1^H NMR (500 MHz, Methanol-*d*_4_) δ 5.03 (dd, *J* = 10.6, 5.4 Hz, 1H), 4.33 (dd, *J* = 9.2, 5.9 Hz, 1H), 3.93 (s, 1H), 3.32–3.23 (m, 2H), 2.65–2.54 (m, 1H), 2.39–2.25 (m, 2H), 1.93–1.78 (m, 2H), 1.75–1.62 (m, 2H), 1.60–1.53 (m, 1H), 1.46 (s, 9H), 1.04–0.97 (m, 12H), 0.96 (d, *J* = 6.5 Hz, 3H). ^13^C NMR (126 MHz, Methanol-*d*_4_) δ 179.48, 172.99, 172.02, 156.37, 118.39, 79.16, 62.02, 51.84, 40.12, 39.97, 38.04, 37.50, 33.92, 33.88, 27.30, 27.09, 25.74, 24.46, 21.83, 20.80.

***(S)-N-((S)-1-Cyano-2-((S)-2-oxopyrrolidin-3-yl)ethyl)-2-((S)-3,3-dimethyl-2-(methylsulfonamido)butanamido)-4-methylpentanamide (6).*** The synthetic method for N-Boc deprotection was reported in previous literature^1^. A solution of **T9** (150 mg, 0.31 mmol) in hexafluoroisopropanol (1.35 mL) was cooled in an ice bath and stirred for 15 min. The solution was then treated with TsOH (63 mg, 0.33 mmol) and stirred for another 5 min before removing the ice bath. After the reaction mixture had been stirred at room temperature for 45 min, it was concentrated. To a 0 °C solution of the reaction residue in DCM (3 mL) was added NMM (86 μL, 0.78 mmol). Then methanesulfonic anhydride (49 μL, 0.37 mmol) was added dropwise. The reaction mixture was warmed to room temperature slowly and stirred for 12 h. DCM and H_2_O were added and then separated. The combined organic phase was washed by saturated aqueous sodium chloride solution, dried over sodium sulfate and concentrated under reduced pressure. Purification via reversed-phase HPLC (Column: Waters Sunfire C18, 19 x 150 mm, 5 µm; Mobile phase A: water containing 0.1% trifluoroacetic acid; Mobile phase B: acetonitrile containing 0.1% trifluoroacetic acid; Gradient: 10% to 50% B over 15 minutes, then 50% to 65% B over 10 min, then 65% to 100% B for 7 min; Flow rate: 10 mL/min) afforded **3** as trifluoroacetic acid salt form. Yield: 21 mg, 15%. HPLC purity: 95.4% (RT = 12.93 min). ^1^H NMR (600 MHz, DMSO-*d*_6_) δ 8.95 (d, *J* = 8.3 Hz, 1H), 8.29 (d, *J* = 7.2 Hz, 1H), 7.68 (s, 1H), 7.13 (d, *J* = 10.2 Hz, 1H), 4.92 (ddd, *J* = 10.5, 8.3, 5.6 Hz, 1H), 4.27–4.22 (m, 1H), 3.62 (d, *J* = 10.2 Hz, 1H), 3.19–3.11 (m, 1H), 3.09–3.02 (m, 1H), 2.78 (s, 3H), 2.39–2.29 (m, 1H), 2.15 (ddd, *J* = 13.5, 10.5, 4.7 Hz, 1H), 2.10–2.02 (m, 1H), 1.77–1.59 (m, 3H), 1.59–1.51 (m, 1H), 1.42–1.34 (m, 1H), 0.92 (d, *J* = 6.6 Hz, 3H), 0.90 (s, 9H), 0.85 (d, *J* = 6.6 Hz, 3H). ^13^C NMR (151 MHz, DMSO-*d*_6_) δ 177.40, 171.91, 170.10, 119.58, 63.71, 51.07, 40.04, 40.03, 39.62, 37.85, 36.84, 34.01, 33.66, 26.84, 26.50, 24.17, 22.89, 21.22. ESI-HRMS Calcd for C_20_H_36_N_5_O_5_S [M+H]^+^: 458.2432, found 458.2435.

Synthesis of **7**

***(S)-N-((S)-1-Cyano-2-((S)-2-oxopyrrolidin-3-yl)ethyl)-2-((S)-2-(cyclopropanesulfonamido)-3,3-dimethylbutanamido)-4-methylpentanamide (7).*** Compound **7** was prepared according to the procedure of compound **6**. Trifluoroacetic acid salt form, yield: 15 mg, 10%. HPLC purity: 97.2% (RT = 13.57 min). ^1^H NMR (600 MHz, DMSO-*d*_6_) δ 8.97 (d, *J* = 8.3 Hz, 1H), 8.22 (d, *J* = 6.9 Hz, 1H), 7.68 (s, 1H), 7.12 (d, *J* = 10.3 Hz, 1H), 4.91 (ddd, *J* = 10.6, 8.3, 5.5 Hz, 1H), 4.22 (ddd, *J* = 9.5, 6.9, 5.7 Hz, 1H), 3.59 (d, *J* = 10.3 Hz, 1H), 3.19–3.11 (m, 1H), 3.10–3.01 (m, 1H), 2.41–2.28 (m, 2H), 2.14 (ddd, *J* = 13.4, 10.5, 4.6 Hz, 1H), 2.09–2.02 (m, 1H), 1.76–1.67 (m, 2H), 1.66–1.58 (m, 1H), 1.55–1.48 (m, 1H), 1.43–1.35 (m, 1H), 0.95–0.91 (m, 4H), 0.91–0.87 (m, 10H), 0.85 (d, *J* = 6.6 Hz, 3H), 0.82–0.78 (m, 2H). ^13^C NMR (151 MHz, DMSO) δ 177.43, 171.98, 170.34, 119.58, 63.80, 51.19, 40.30, 40.06, 37.83, 36.82, 34.17, 33.69, 30.01, 26.84, 26.57, 24.17, 22.82, 21.47, 5.18, 4.42. ESI-HRMS Calcd for C_22_H_38_N_5_O_5_S [M+H]^+^: 484.2588, found 484.2589.

Synthesis of **8**

***(2S)-2-((Tert-butoxycarbonyl)amino)-3-(2-oxopyrrolidin-3-yl)propyl benzoate (T10).*** To a solution of tert-butyl ((2S)-1-hydroxy-3-(2-oxopyrrolidin-3-yl)propan-2-yl)carbamate (1.0 g, 3.87 mmol) in pyridine (5 mL) was added benzoyl chloride (540 μL, 540 mmol) dropwise under an ice bath. The reaction was allowed to slowly warm to room temperature and stirred for 2 hours. Upon completion, the reaction mixture was extracted with ethyl acetate. The organic phase was washed with saturated brine, dried over anhydrous sodium sulfate, and concentrated. The residue was purified by column chromatography (Gradient: 0%–50% EA in PE) to provide **T10** as a white solid. Yield, 1.1 g, 91%. ^1^H NMR (500 MHz, Chloroform-*d*) δ 8.10–8.02 (m, 2H), 7.62–7.56 (m, 1H), 7.51–7.42 (m, 2H), 5.76 (s, 1H), 5.01 (d, *J* = 9.3 Hz, 1H), 4.40–4.30 (m, 2H), 4.14–4.05 (m, 1H), 3.45–3.35 (m, 2H), 2.54 (q, *J* = 5.7, 4.7 Hz, 2H), 2.12 (ddd, *J* = 14.6, 11.7, 3.3 Hz, 1H), 1.92–1.82 (m, 1H), 1.59 (ddd, *J* = 14.2, 9.6, 3.4 Hz, 1H), 1.43 (s, 9H).

***(S)-2-((S)-2-((S)-3,3-Dimethyl-2-(2,2,2-trifluoroacetamido)butanamido)-4-methylpentanamido)-3-((S)-2-oxopyrrolidin-3-yl)propyl benzoate (T11).*** A solution of HCl in 1,4-dioxane (4 M; 2.21 mL, 8.83 mmol) was added to a solution of **T10** (350 mg, 0.81 mmol) in DCM (2.00 mL), and the reaction mixture was stirred at room temperature for 2 h. Then the reaction mixture was concentrated, which was used in the next step without further purification. Isobutyl chloroformate (305 μL, 2.06 mmol) and NMM (396 μL, 4.11 mmol) were added to a -20 °C solution of **T2** (700 mg, 2.06 mmol) in THF (15 mL) dropwise. The solution was stirred at -20 °C for 30 min. Then (2S)-2-amino-3-(2-oxopyrrolidin-3-yl)propyl benzoate hydrochloride (615 mg, 2.06 mmol) and NMM (198 μL, 2.06 mmol) was added, and stirring was continued for 16 h. The reaction mixture was then concentrated, extracted with ethyl acetate, washed with saturated brine, and dried over anhydrous sodium sulfate. The residue was purified by column chromatography (Gradient: 0%–50% EA in PE) to yield **T11** as a white solid. Yield, 525 mg, 44%.

***(S)-2-((S)-3,3-Dimethyl-2-(2,2,2-trifluoroacetamido)butanamido)-N-((S)-1-hydroxy-3-((S)-2-oxopyrrolidin-3-yl)propan-2-yl)-4-methylpentanamide (T12).*** Intermediate **T11** (300 mg, 0.51 mmol) was dissolved in methanol (5 mL), followed by the addition of potassium carbonate (223 mg, 1.62 mmol). The reaction was stirred at room temperature for 2 hours. Upon completion, the reaction mixture was concentrated and purified by column chromatography (Gradient: 0%–10% methanol in DCM) to afford **T12** as a white solid. Yield, 160 mg, 65%. ^1^H NMR (400 MHz, DMSO-*d*_6_) δ 9.14 (d, *J* = 9.4 Hz, 1H), 8.19 (d, *J* = 7.4 Hz, 1H), 7.79 (d, *J* = 9.1 Hz, 1H), 7.49 (s, 1H), 4.64 (t, *J* = 5.8 Hz, 1H), 4.41 (d, *J* = 9.4 Hz, 1H), 4.22 (q, *J* = 7.5 Hz, 1H), 3.83–3.70 (m, 2H), 3.27–3.20 (m, 1H), 3.18–3.09 (m, 1H), 3.06–2.95 (m, 1H), 2.32–2.20 (m, 1H), 2.20–2.08 (m, 1H), 1.80–1.69 (m, 1H), 1.63–1.52 (m, 2H), 1.47–1.35 (m, 2H), 0.93 (s, 9H), 0.91–0.82 (m, 6H).

***(S)-2-((S)-3,3-Dimethyl-2-(2,2,2-trifluoroacetamido)butanamido)-4-methyl-N-((S)-1-oxo-3-((S)-2-oxopyrrolidin-3-yl)propan-2-yl)pentanamide (8).*** Intermediate **T12** (80 mg, 0.166 mmol) was dissolved in DCM (1 mL), followed by the addition of (diacetoxyiodo)benzene (80 mg, 0.250 mmol) and TEMPO (5 mg, 0.03 mmol). The reaction was stirred at room temperature for 4 hours. Upon completion, the reaction mixture was concentrated and purified by column chromatography (Gradient: 0%–5% methanol in DCM) to yield **8** as a white solid. Yield, 43 mg, 54%. Further purification via reversed-phase HPLC (Column: Waters Sunfire C18, 19 x 150 mm, 5 µm; Mobile phase A: water containing 0.1% trifluoroacetic acid; Mobile phase B: acetonitrile containing 0.1% trifluoroacetic acid; Gradient: 10% to 50% B over 15 minutes, then 50% to 65% B over 10 min, then 65% to 100% B for 7 min; Flow rate: 10 mL/min) afforded **8** as trifluoroacetic acid salt form. HPLC purity: 95.9% (RT = 12.73 min). ^1^H NMR (500 MHz, Chloroform-*d*) δ 9.52 (s, 1H), 8.21 (d, *J* = 6.3 Hz, 1H), 7.20 (d, *J* = 9.2 Hz, 1H), 6.64 (d, *J* = 7.4 Hz, 2H), 4.56 (td, *J* = 8.2, 5.6 Hz, 1H), 4.43 (q, *J* = 6.9 Hz, 1H), 4.35 (d, *J* = 9.2 Hz, 1H), 3.44 (dtd, *J* = 16.7, 10.0, 7.4 Hz, 2H), 2.65–2.53 (m, 1H), 2.50–2.40 (m, 1H), 2.01 (t, *J* = 7.1 Hz, 2H), 1.95–1.85 (m, 1H), 1.77–1.65 (m, 2H), 1.65–1.54 (m, 1H), 1.04 (s, 9H), 0.98 (dd, *J* = 11.0, 6.2 Hz, 6H). ^19^F NMR (471 MHz, Chloroform-*d*) δ -75.61 (trifluoroacetic acid as counterion), -76.06. ^13^C NMR (126 MHz, Chloroform-*d*) δ 198.65, 180.88, 172.84, 168.94, 157.13 (q, *J* = 37.6 Hz), 115.82 (q, *J* = 287.6 Hz), 61.08, 57.89, 52.26, 41.70, 41.10, 38.43, 35.37, 29.66, 28.53, 26.42, 24.85, 22.67, 22.04. ESI-HRMS Calcd for C_21_H_34_F_3_N_4_O_5_ [M+H]^+^: 479.2476, found 479.2480.

Synthesis of **9**

***Benzyl ((S)-3,3-dimethyl-2-(methylsulfonamido)butanoyl)-L-leucinate (T13).*** Intermediate **T13** was prepared according to the procedure of compound **T6**. Yield, 1.3 g, 92%. ^1^H NMR (500 MHz, Chloroform-*d*) δ 7.41–7.31 (m, 5H), 6.04 (d, *J* = 8.3 Hz, 1H), 5.24–5.11 (m, 3H), 4.75–4.66 (m, 1H), 3.54 (d, *J* = 9.8 Hz, 1H), 2.88 (s, 3H), 1.72–1.65 (m, 1H), 1.64–1.55 (m, 2H), 1.01 (s, 9H), 0.92 (t, *J* = 5.9 Hz, 6H).

***((S)-3,3-Dimethyl-2-(methylsulfonamido)butanoyl)-L-leucine (T14).*** Intermediate **T14** was prepared according to the procedure of compound **T2**. Yield, 900 mg, 89%.

***Methyl (S)-2-((S)-2-((S)-3,3-dimethyl-2-(methylsulfonamido)butanamido)-4-methylpentanamido)-3-((S)-2-oxopyrrolidin-3-yl)propanoate (T15).*** Intermediate **T15** was prepared according to the procedure of compound **T11**. Yield, 900 mg, 89%.^1^H NMR (500 MHz, Chloroform-*d*) δ 8.01 (d, *J* = 7.0 Hz, 1H), 6.72 (d, *J* = 8.4 Hz, 1H), 6.17 (s, 1H), 5.51 (d, *J* = 9.8 Hz, 1H), 4.69–4.56 (m, 1H), 4.49 (ddd, *J* = 11.2, 7.0, 3.9 Hz, 1H), 3.72 (s, 3H), 3.58 (d, *J* = 9.8 Hz, 1H), 3.44–3.26 (m, 2H), 2.51–2.34 (m, 2H), 2.15 (ddd, *J* = 14.3, 11.6, 5.7 Hz, 1H), 1.97–1.80 (m, 2H), 1.74–1.68 (m, 2H), 1.61–1.54 (m, 1H), 1.02 (s, 9H), 0.96 (t, *J* = 6.1 Hz, 6H).

***(S)-2-((S)-3,3-Dimethyl-2-(methylsulfonamido)butanamido)-N-((S)-1-hydroxy-3-((S)-2-oxopyrrolidin-3-yl)propan-2-yl)-4-methylpentanamide (T16).*** To a solution of **T15** (320 mg, 0.65 mmol) in anhydrous tetrahydrofuran (4 mL) was added lithium borohydride (0.28 mg, 1.30 mmol) at 0 °C. The reaction mixture was then allowed to warm to room temperature and stirred for 2 hours. After the reaction was complete, ice water was added to quench the reaction. The mixture was extracted using a DCM/n-butanol solvent mixture (v/v = 3:1). The organic layer was washed with saturated brine, concentrated, and purified by column chromatography (Gradient: 0%–10% MeOH in DCM) to yield **T16** as a white solid. Yield, 183 mg, 61%. ^1^H NMR (500 MHz, Chloroform-*d*) δ 7.83 (d, *J* = 6.8 Hz, 1H), 6.64 (d, *J* = 8.2 Hz, 1H), 5.80 (s, 1H), 5.43 (d, *J* = 9.4 Hz, 1H), 4.52 (td, *J* = 8.9, 5.0 Hz, 1H), 3.98 (t, *J* = 4.6 Hz, 1H), 3.64–3.55 (m, 3H), 3.36 (dd, *J* = 9.1, 6.3 Hz, 2H), 2.93 (s, 3H), 2.51–2.36 (m, 2H), 2.05–1.92 (m, 1H), 1.91–1.80 (m, 1H), 1.73–1.64 (m, 4H), 1.03 (s, 9H), 0.96 (dt, *J* = 6.2, 5.3 Hz, 6H).

***(S)-2-((S)-3,3-Dimethyl-2-(methylsulfonamido)butanamido)-4-methyl-N-((S)-1-oxo-3-((S)-2-oxopyrrolidin-3-yl)propan-2-yl)pentanamide (9).*** To a solution of **T15** (183 mg, 0.40 mmol) in DCM (3 mL) was added Dess-Martin periodinane (503 mg, 1.19 mmol) and sodium bicarbonate (133 mg, 1.58 mmol). The reaction was stirred at room temperature for 24 hours. After completion, the reaction was quenched by adding a saturated sodium bicarbonate solution containing 10% sodium thiosulfate and stirred until clear. The mixture was extracted with a DCM/n-butanol solvent mixture (v/v = 3:1), and the organic phase was washed with saturated brine and concentrated. The product was purified by column chromatography (Gradient: 0%–10% MeOH in DCM) to yield **9** as a white solid. Yield, 70 mg, 38%. Further purification via reversed-phase HPLC (Column: Waters Sunfire C18, 19 x 150 mm, 5 µm; Mobile phase A: water containing 0.1% trifluoroacetic acid; Mobile phase B: acetonitrile containing 0.1% trifluoroacetic acid; Gradient: 10% to 50% B over 15 minutes, then 50% to 65% B over 10 min, then 65% to 100% B for 7 min; Flow rate: 10 mL/min) afforded **9** as trifluoroacetic acid salt form. HPLC purity: 95.5% (RT = 11.18 min). ^1^H NMR (500 MHz, Chloroform-*d*) δ 9.50 (s, 1H), 8.07 (d, *J* = 6.6 Hz, 1H), 6.79–6.71 (m, 2H), 5.48 (d, *J* = 8.9 Hz, 1H), 4.59 (td, *J* = 8.6, 5.0 Hz, 1H), 4.45–4.38 (m, 1H), 3.60 (d, *J* = 9.0 Hz, 1H), 3.46–3.35 (m, 2H), 2.93 (s, 3H), 2.58 (ddd, *J* = 15.9, 9.2, 6.7 Hz, 1H), 2.43 (dtd, *J* = 15.6, 8.7, 7.9, 2.8 Hz, 1H), 2.02–1.95 (m, 2H), 1.91–1.84 (m, 1H), 1.74–1.69 (m, 1H), 1.68–1.60 (m, 2H), 1.03 (s, 9H), 0.96 (dd, *J* = 13.1, 6.1 Hz, 6H). ^13^C NMR (126 MHz, Chloroform-*d*) δ 198.95, 181.20, 172.96, 170.68, 65.54, 57.66, 52.16, 41.22, 40.31, 38.41, 34.53, 29.79, 28.37, 26.63, 24.89, 22.93, 21.57. ESI-HRMS Calcd for C_20_H_37_N_4_O_6_S [M+H]^+^: 461.2428, found 461.2428.

Synthesis of **10**

***Sodium (2S)-2-((S)-2-((S)-3,3-dimethyl-2-(2,2,2-trifluoroacetamido)butanamido)-4-methylpentanamido)-1-hydroxy-3-((S)-2-oxopyrrolidin-3-yl)propane-1-sulfonate (10).*** To a stirred solution of **8** (500 mg, 0.86 mmol) in EA (5 mL) and EtOH (2.5 mL) at room temperature was added a solution of NaHSO3 (89 mg, 0.86 mmol) in water (178 μL). The reaction mixture was heated at 50 °C for 3.5 h. After completion of the reaction, the reaction mixture was cooled to room temperature and filtered. The filtrate was concentrated under reduced pressure and dissolved in water (20 mL). The aqueous layer was washed with EA (5 x 6 mL), concentrated down to remove remaining EA, and then lyophilized to provide the desired product **10** as white solid. Yield, 420 mg, 84%. ^1^H NMR (500 MHz, DMSO-*d*_6_) δ 9.14 (d, *J* = 9.1 Hz, 1H), 8.23–8.12 (m, 1H), 7.72 (d, *J* = 9.3 Hz, 0.5H), 7.44 (d, *J* = 9.4 Hz, 0.5H), 7.41 (d, *J* = 5.1 Hz, 1H), 5.25 (d, *J* = 5.7 Hz, 0.5H), 5.18 (d, *J* = 5.8 Hz, 0.5H), 4.40 (dd, *J* = 9.0, 5.7 Hz, 1H), 4.30–4.21 (m, 1H), 4.18–4.11 (m, 0.5H), 4.06–3.96 (m, 0.5H), 3.91 (dd, *J* = 5.7, 2.4 Hz, 0.5H), 3.79 (t, *J* = 5.5 Hz, 0.5H), 3.10 (t, *J* = 9.3 Hz, 1H), 3.04–2.92 (m, 1H), 2.26–2.06 (m, 2H), 1.97–1.85 (m, 1H), 1.77–1.50 (m, 3H), 1.49–1.36 (m, 2H), 0.93 (s, 9H), 0.91–0.87 (m, 3H), 0.86–0.81 (m, 3H). ^13^C NMR (126 MHz, DMSO) δ 179.82, 179.57, 171.56, 171.47, 168.59, 168.49, 156.76 (q, *J* = 36.7 Hz), 117.54, 115.25, 85.37, 84.58, 60.90, 60.88, 52.34, 52.07, 49.25, 48.65, 41.13, 40.71, 38.01, 37.89, 35.13, 33.24, 30.09, 28.13, 27.88, 27.00, 26.98, 24.68, 24.51, 23.38, 23.31, 22.45, 22.31. ESI-HRMS Calcd for C_21_H_33_F_3_N_4_NaO_8_S [M-H]^-^: 581.1875, found 581.1874.

**Spectroscopic Data**

^1^H NMR Spectra for **1**


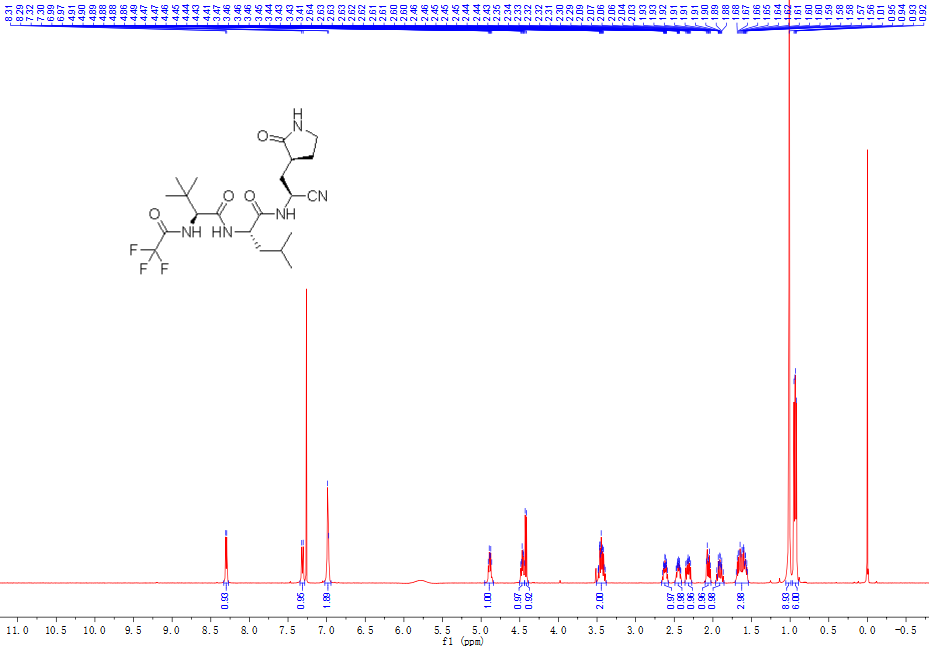


^13^C NMR Spectra for **1**


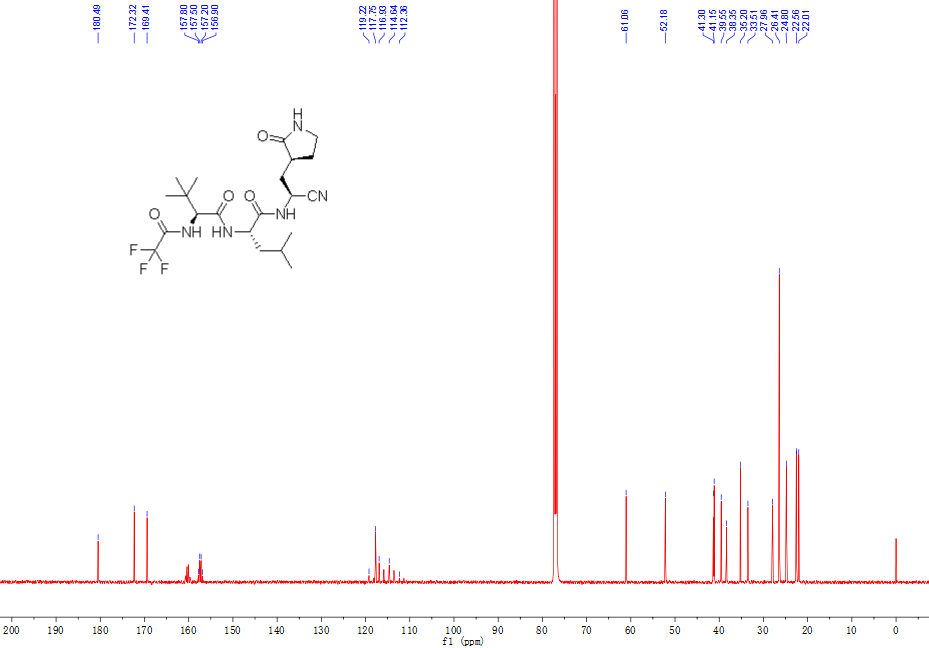


HRMS Spectra for **1**

**
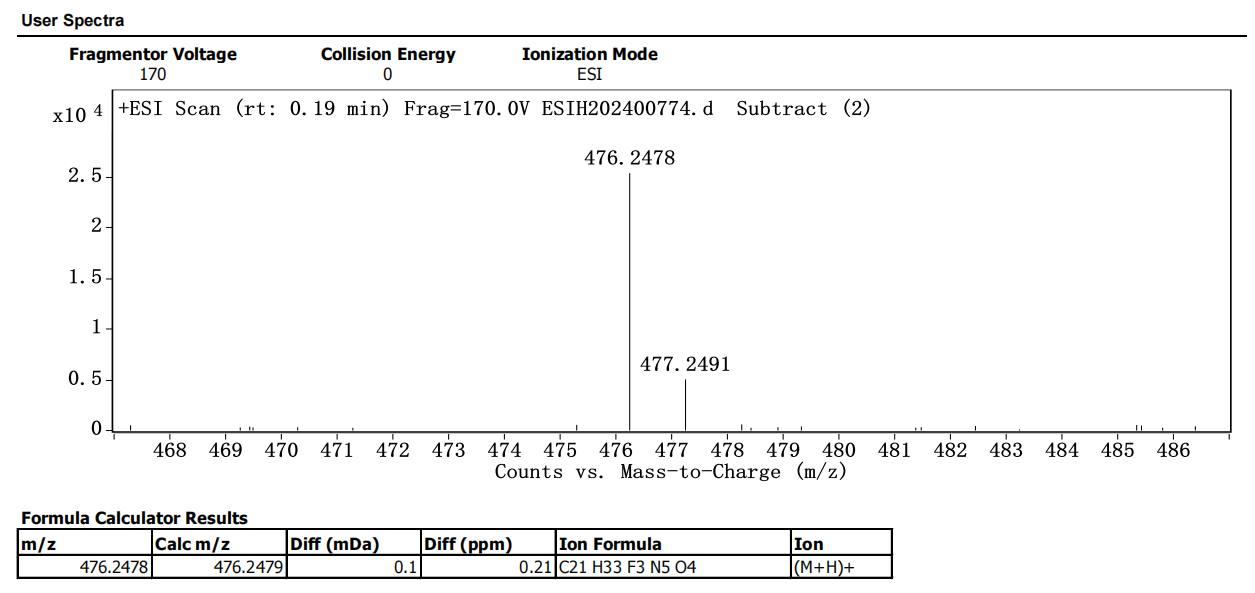
**

HPLC Trace for **1**

**
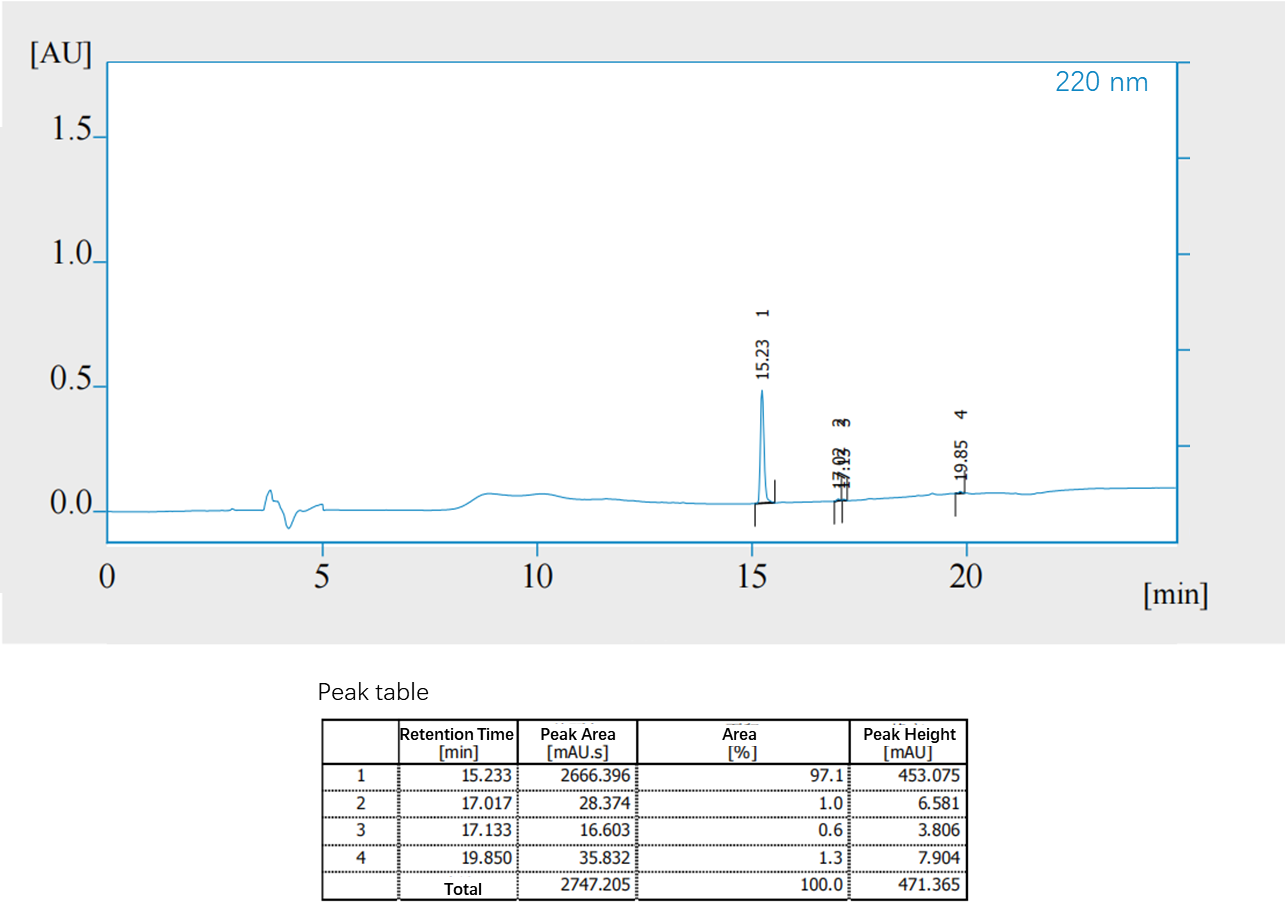
**

^1^H NMR Spectra for **2**

**
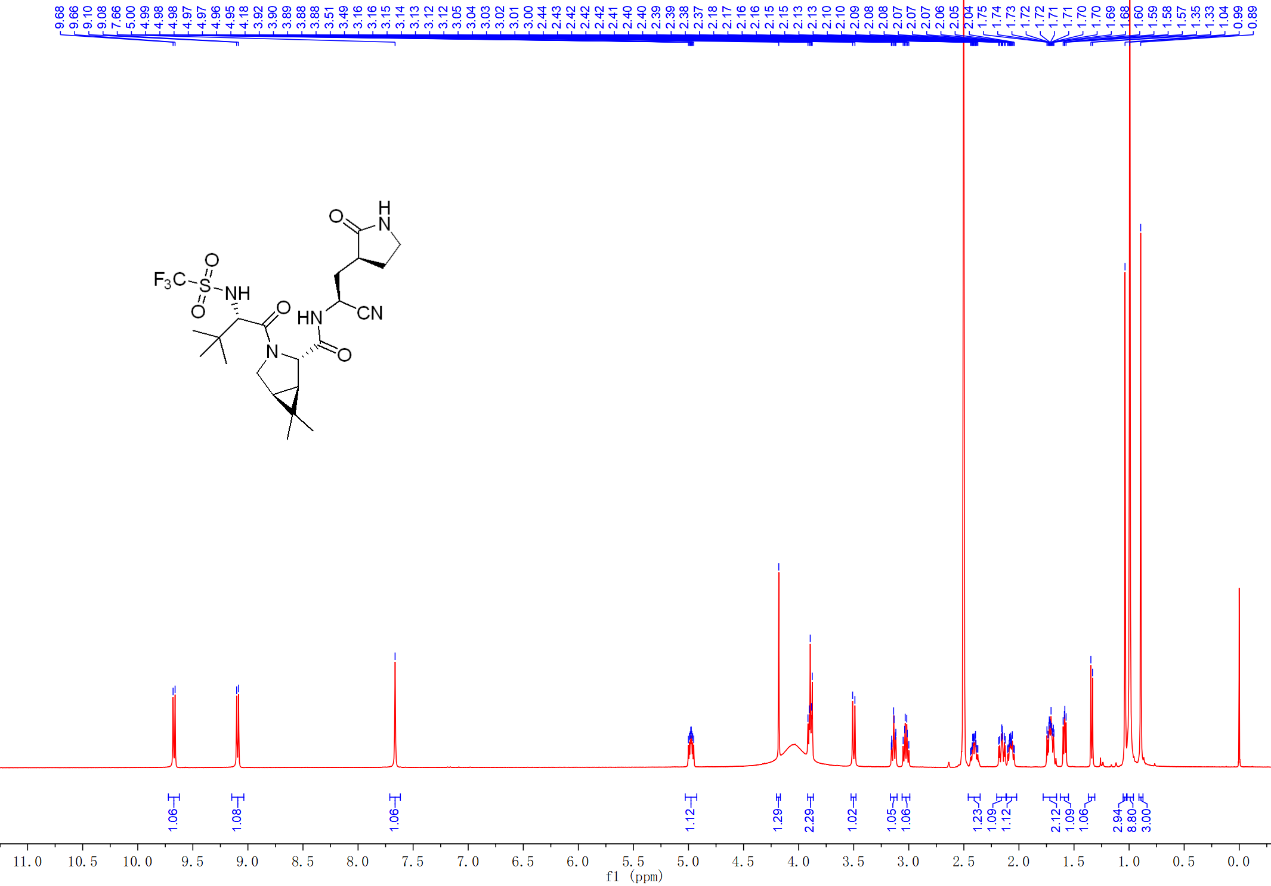
**

^13^C NMR Spectra for **2**


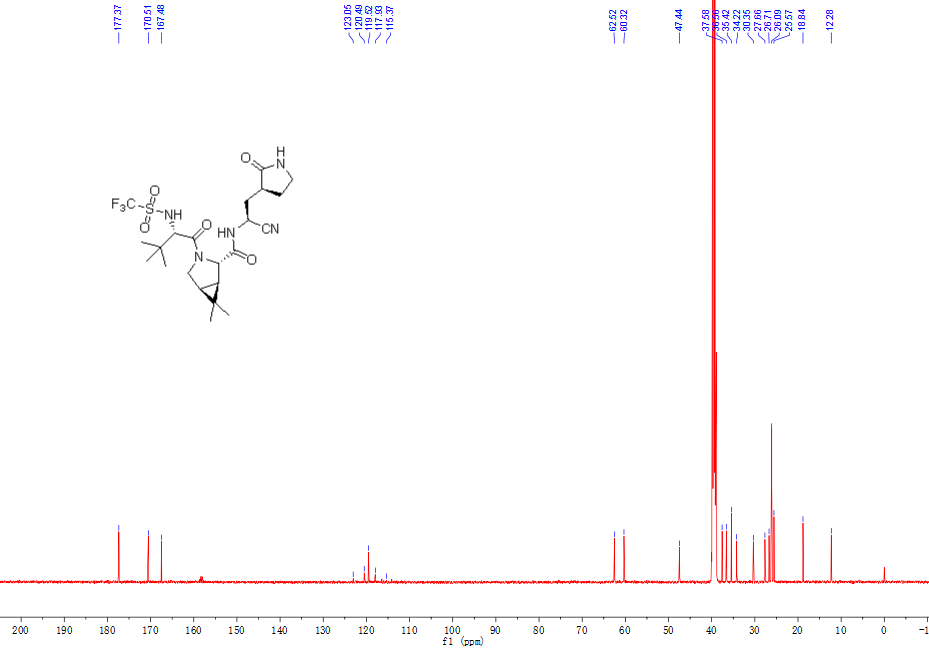


HRMS Spectra for **2**

**
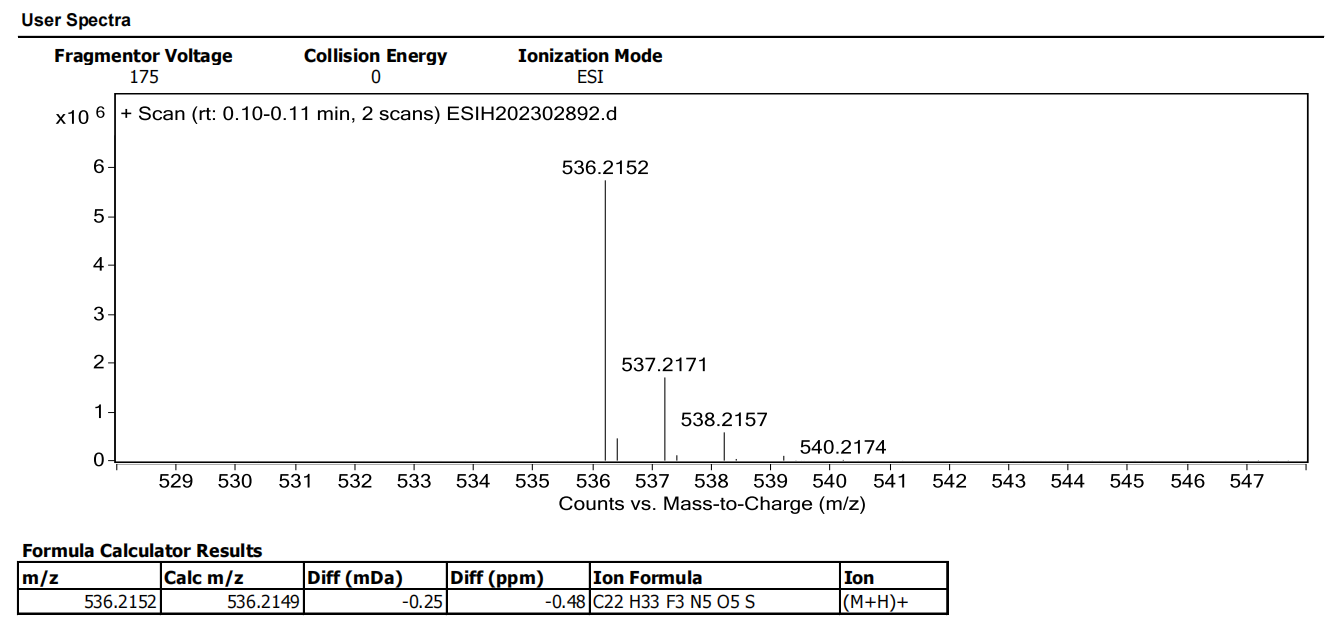
**

HPLC Trace for **2**

**
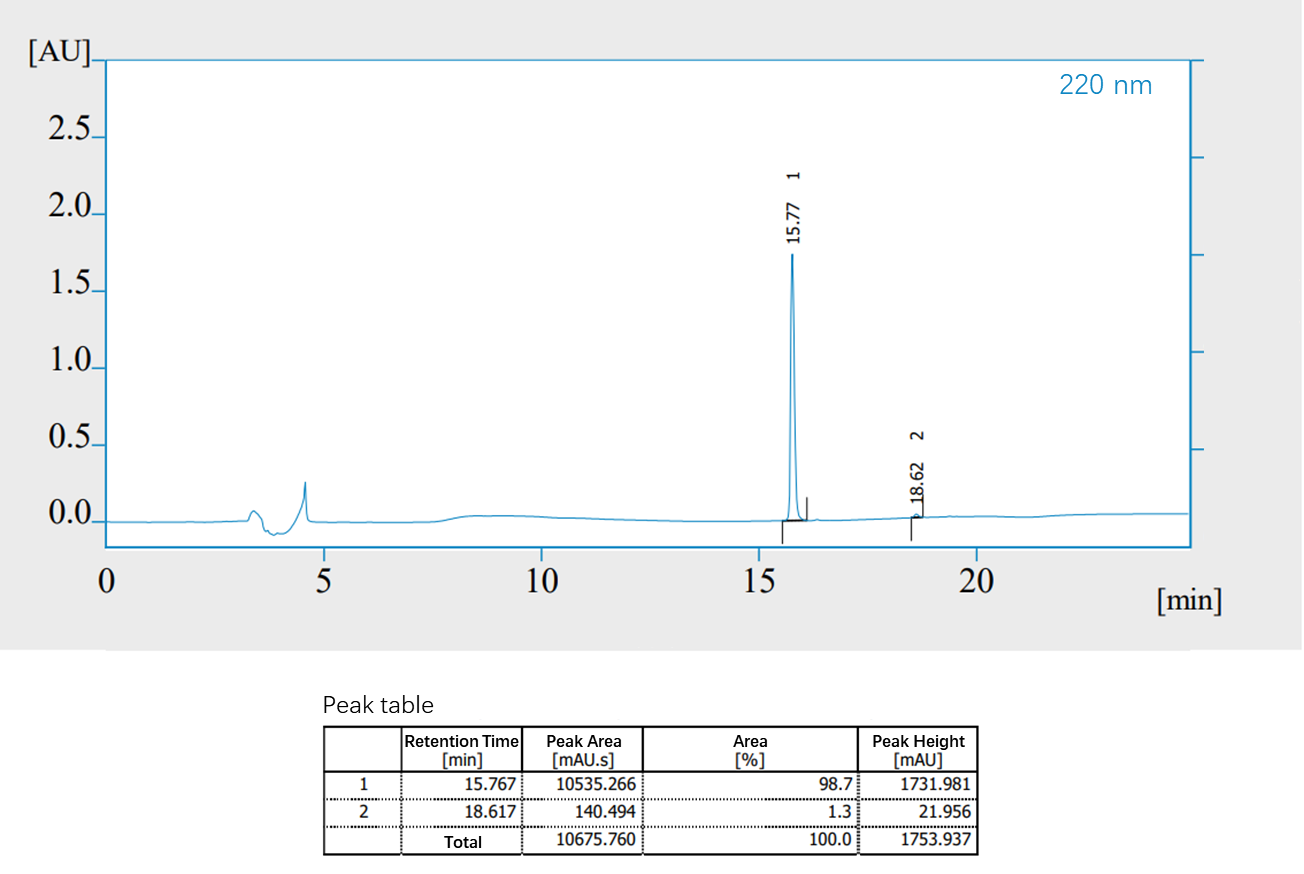
**

^1^H NMR Spectra for **3**

**
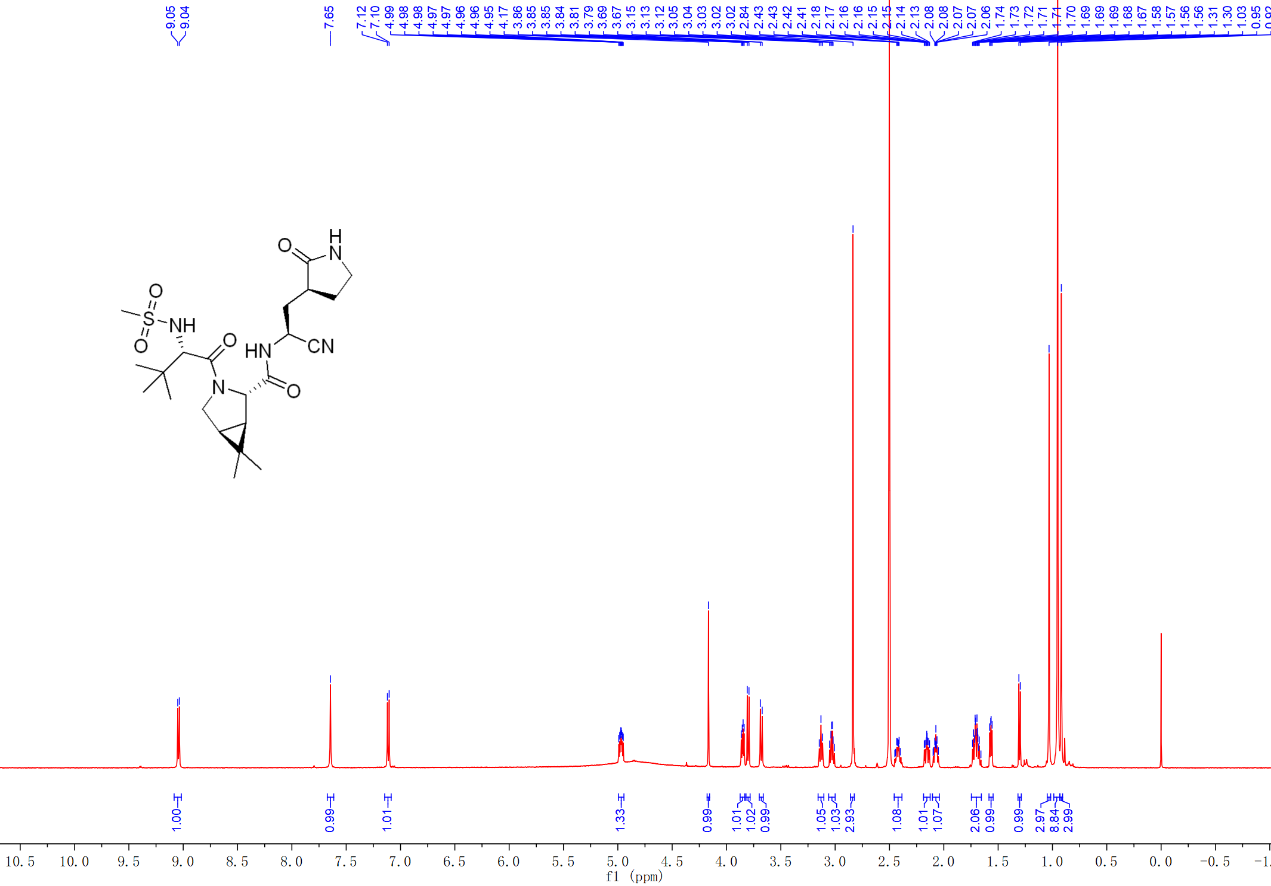
**

^13^C NMR Spectra for **3**


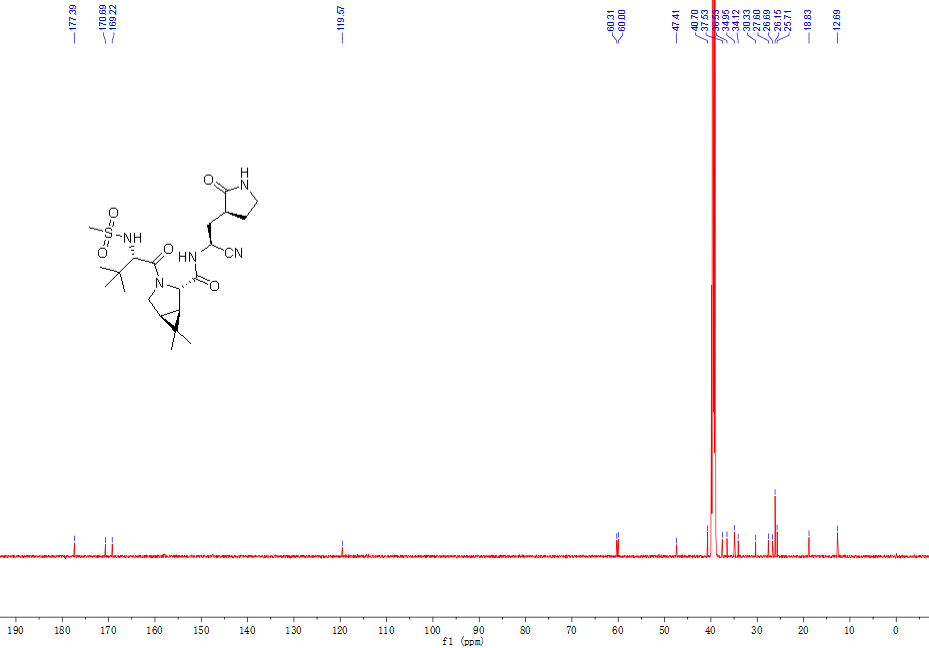


HRMS Spectra for **3**

**
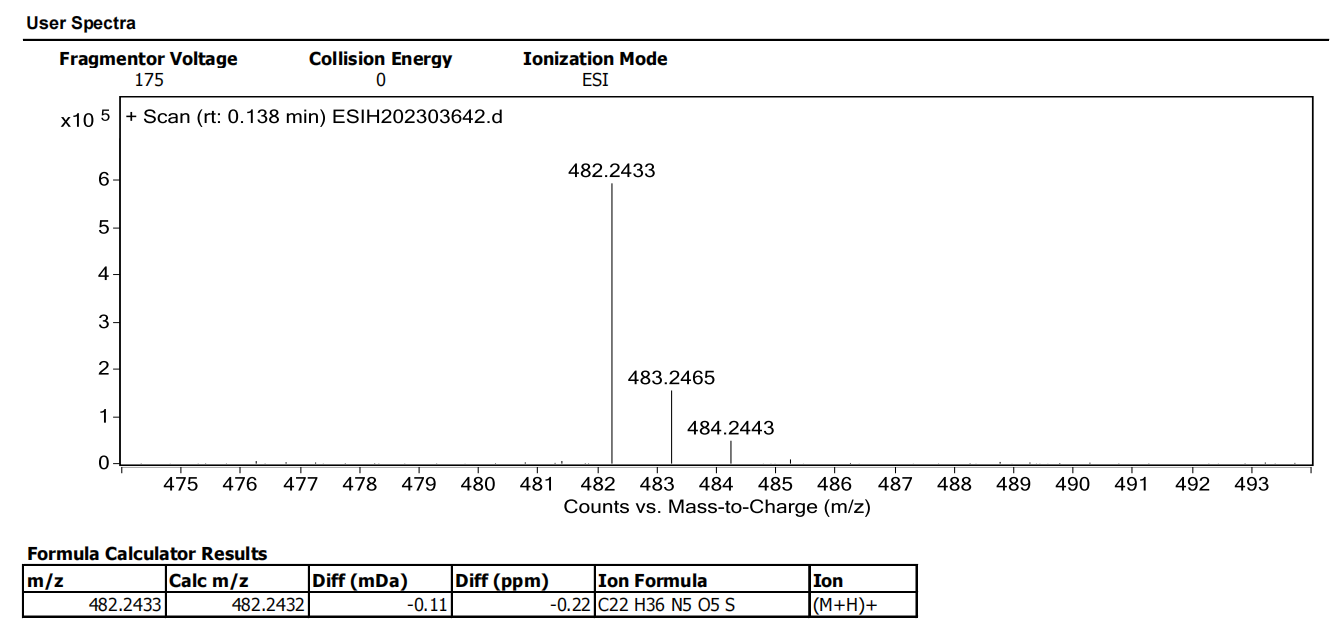
**

HPLC Trace for **3**

**
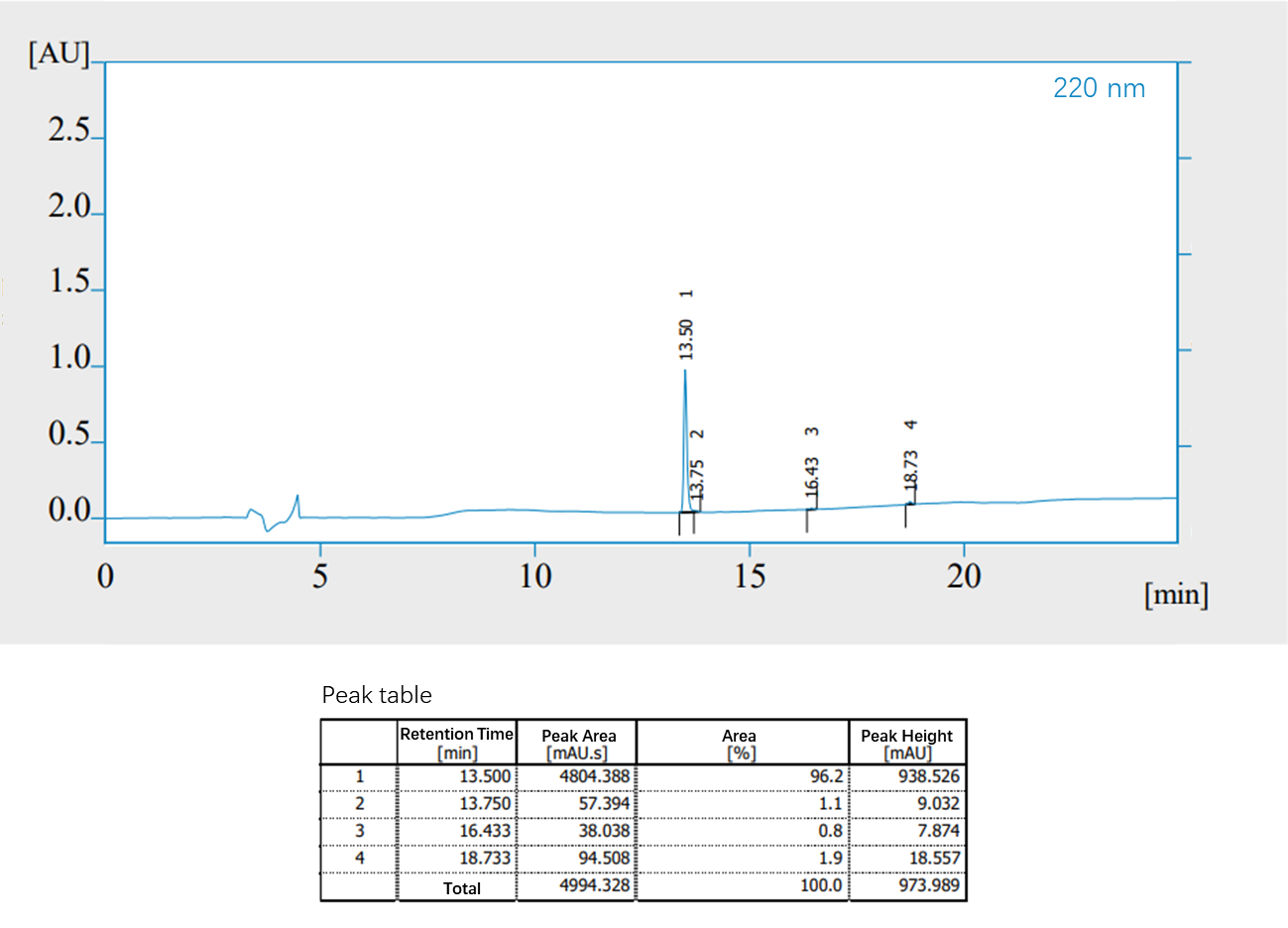
**

^1^H NMR Spectra for **4**

**
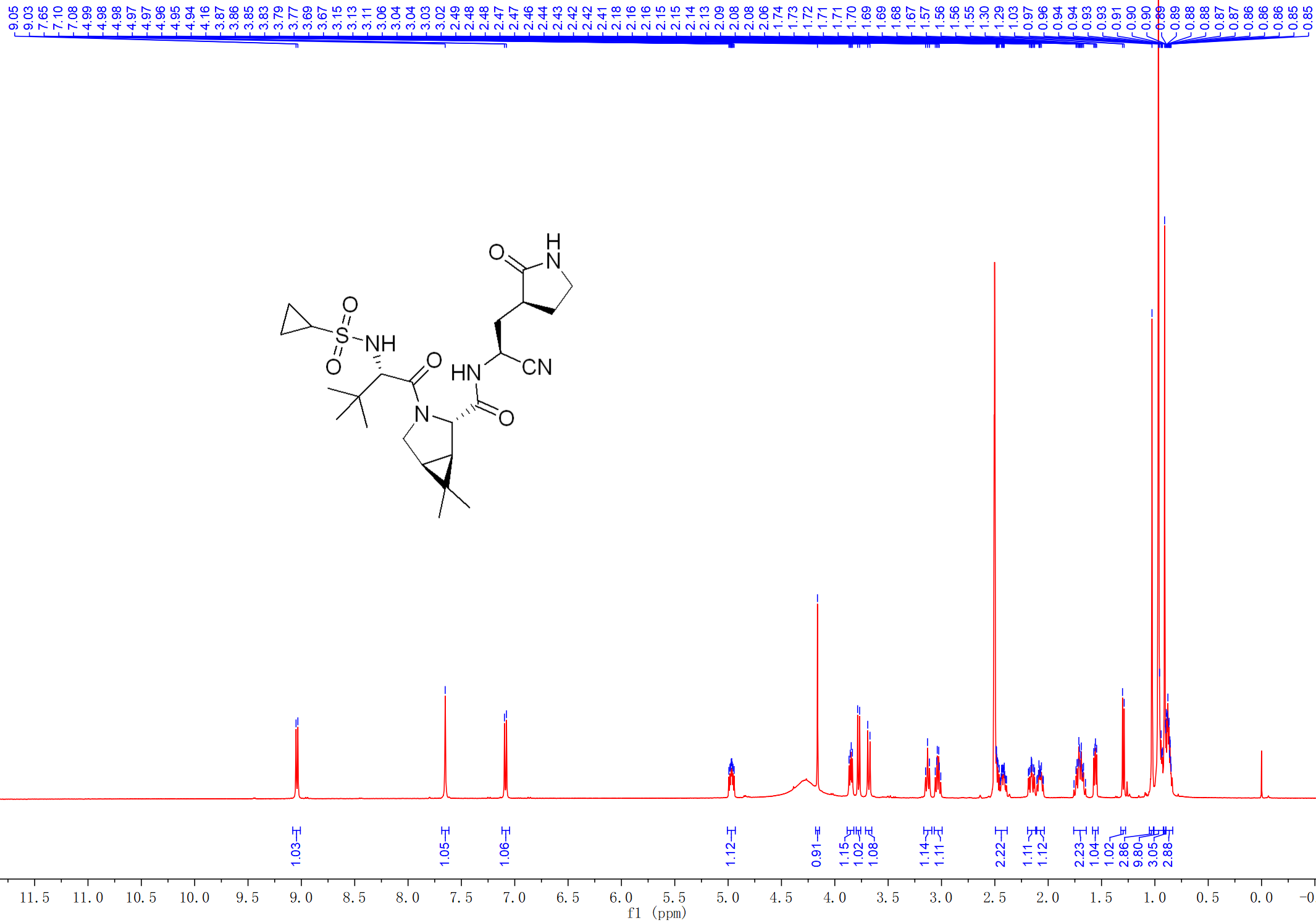
**

^13^C NMR Spectra for **4**

**
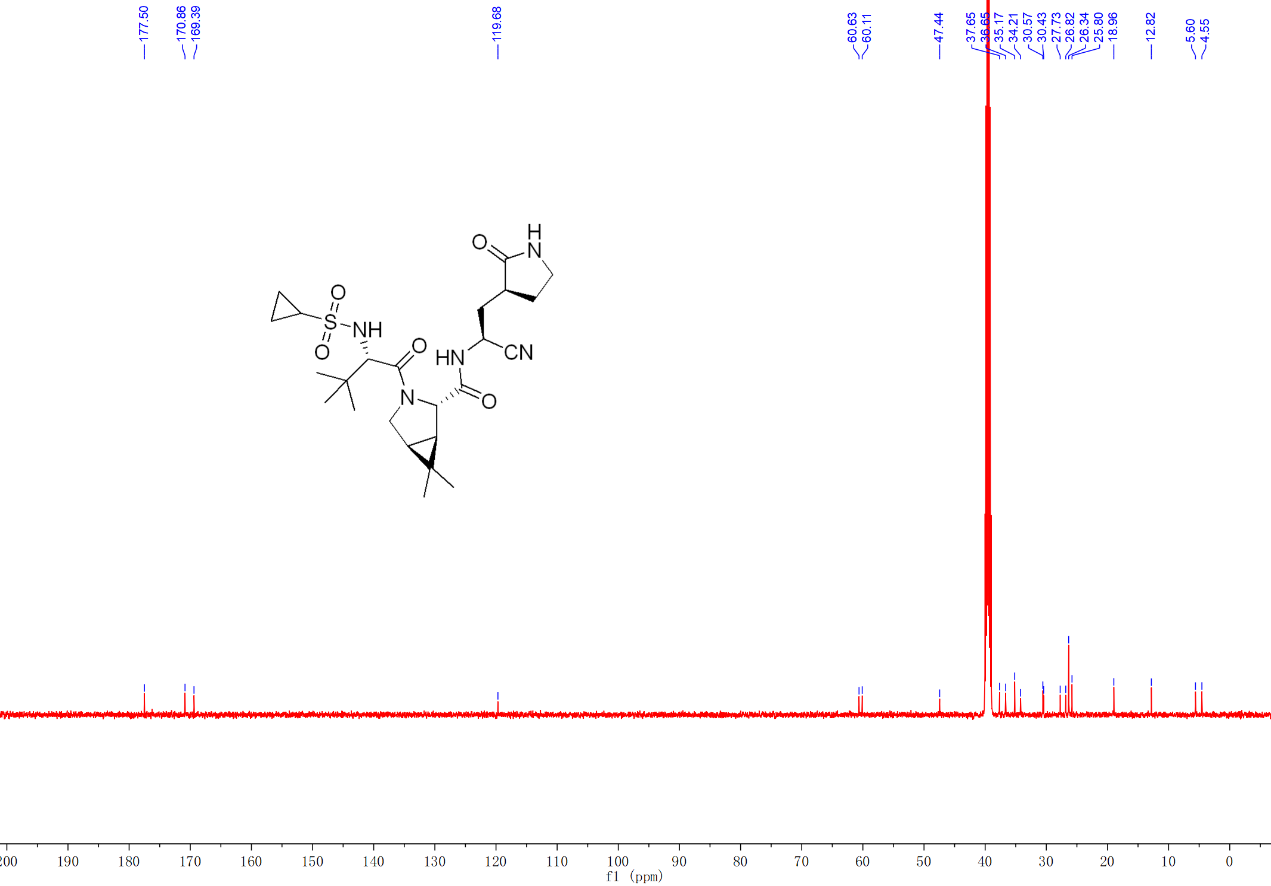
**

HRMS Spectra for **4**

**
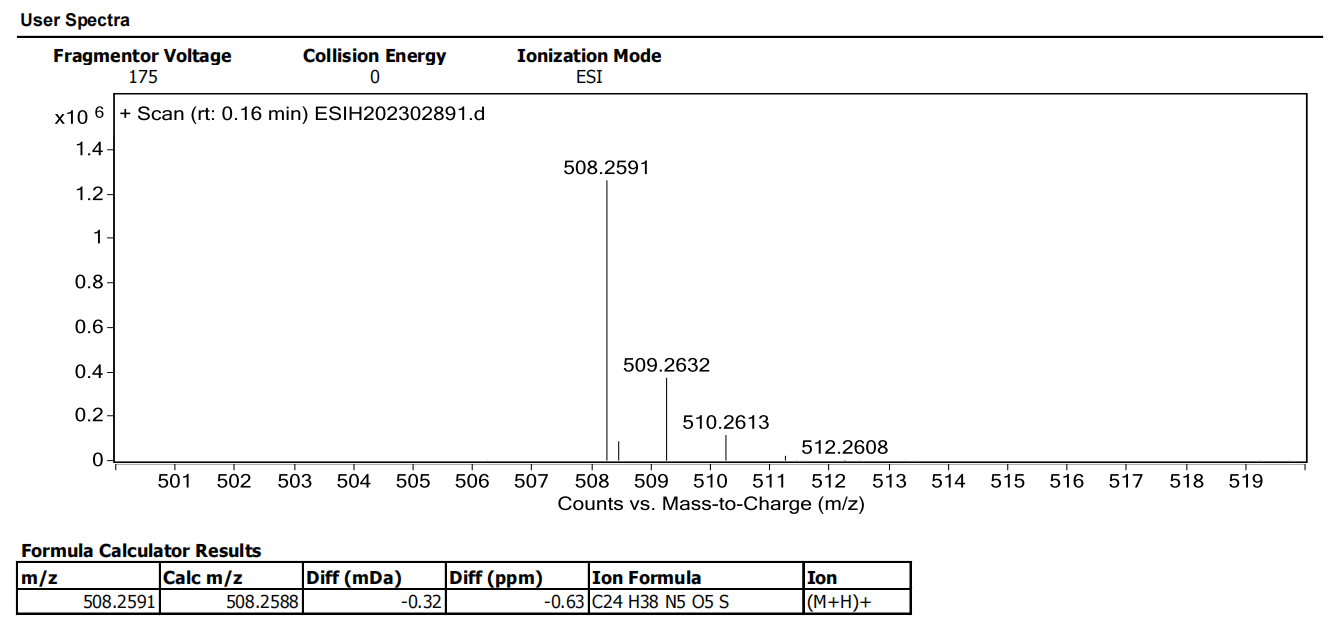
**

HPLC Trace for **4**

**
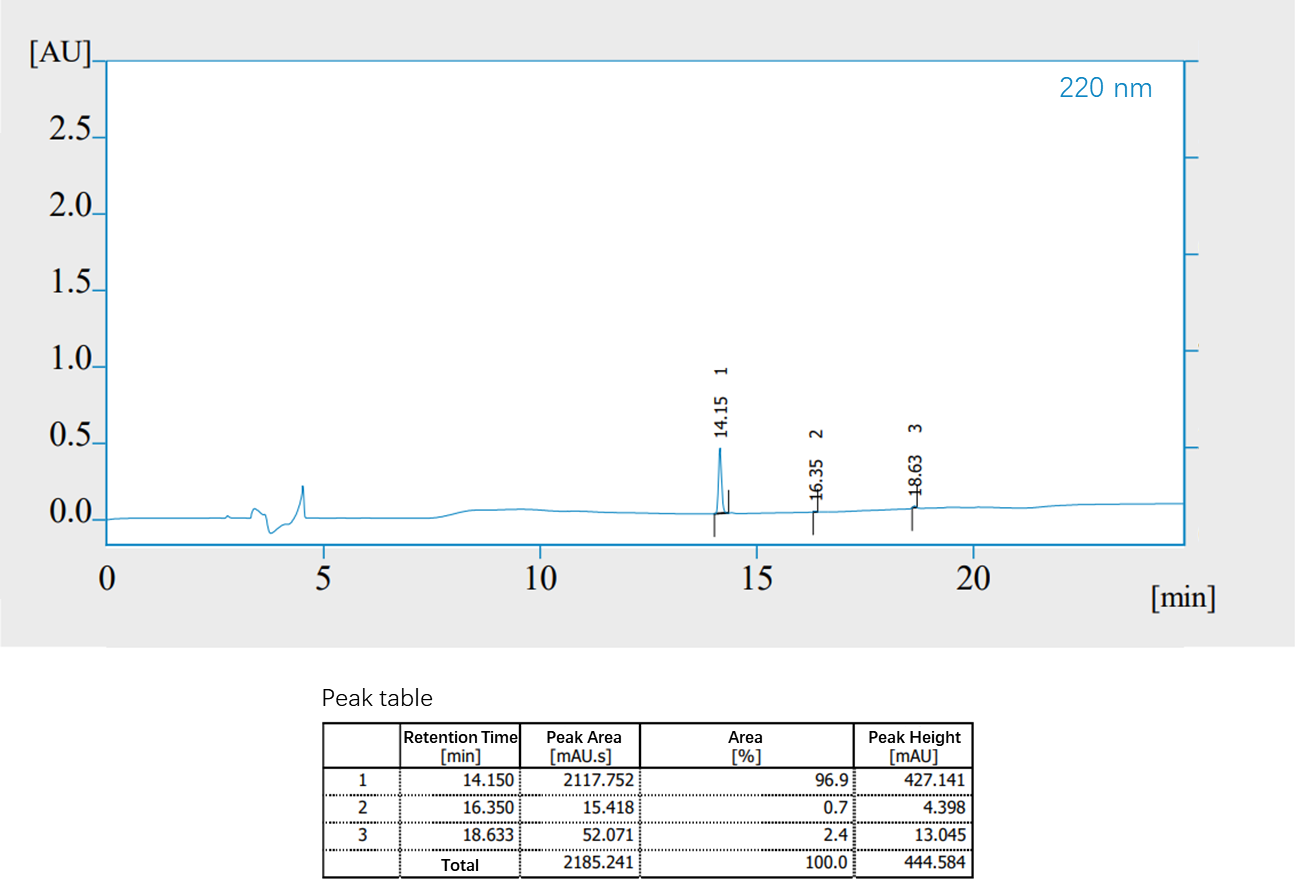
**

^1^H NMR Spectra for **5**

**
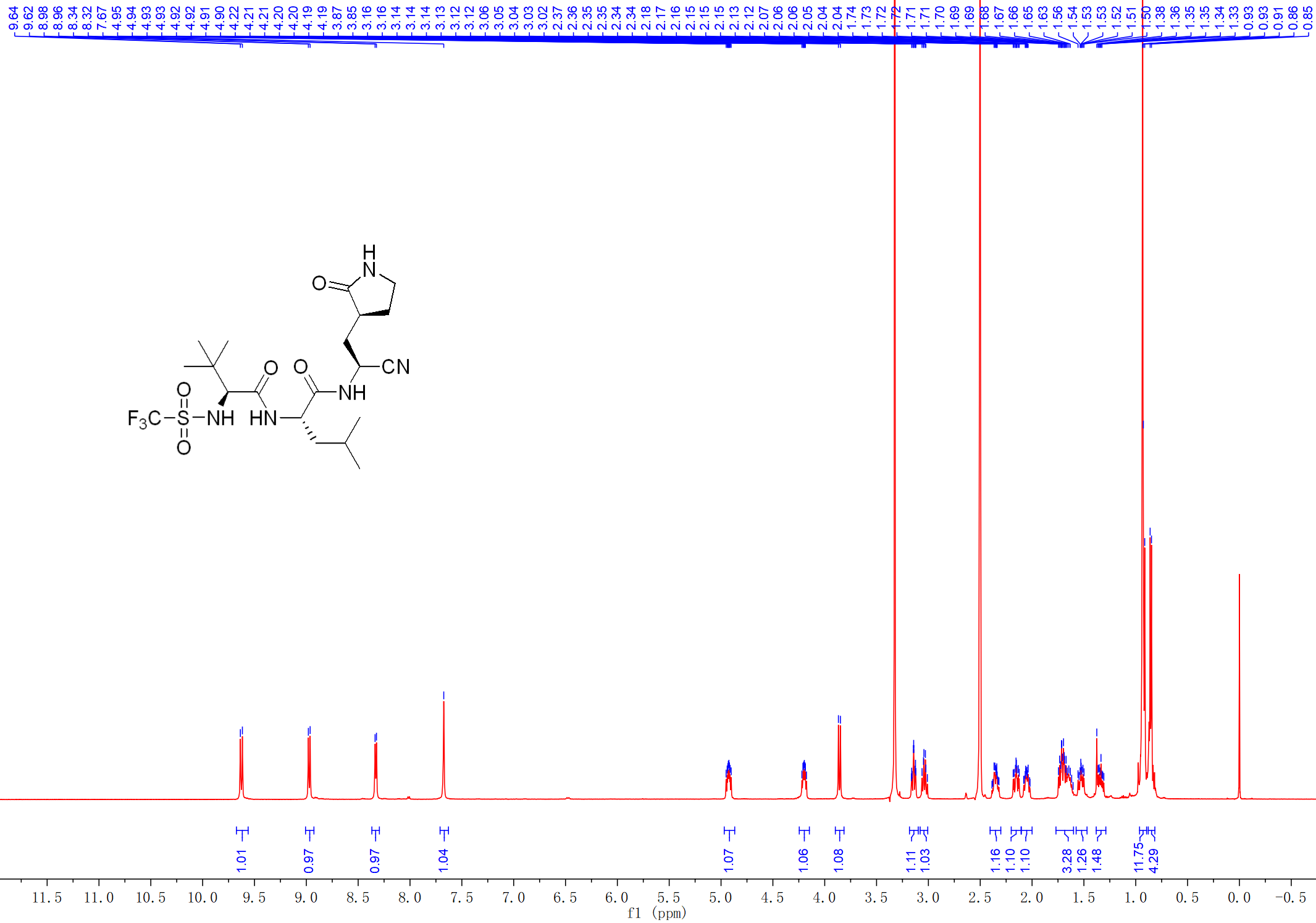
**

^13^C NMR Spectra for **5**

~~
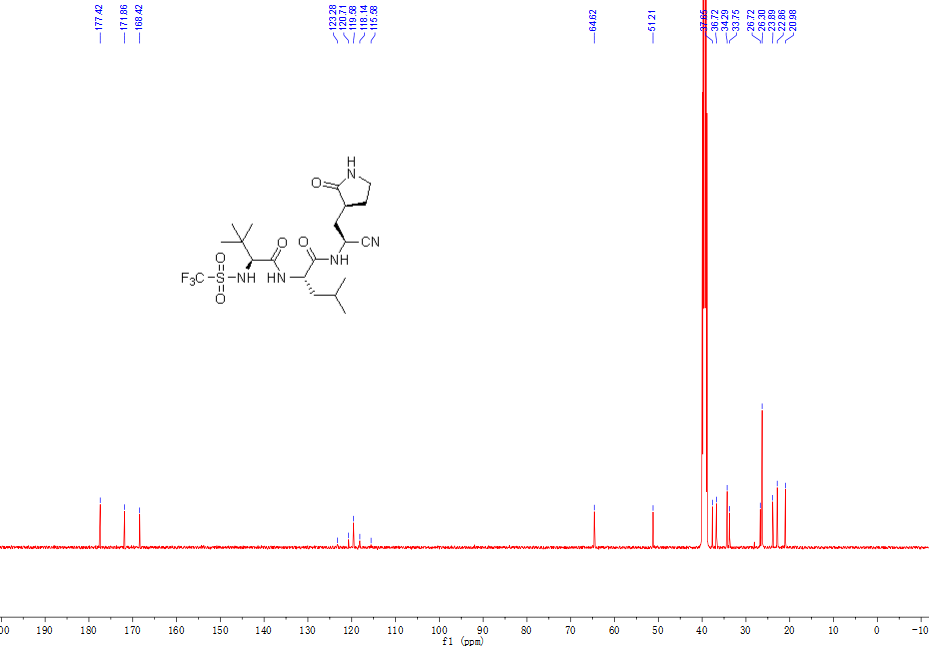
~~

HRMS Spectra for **5**

**
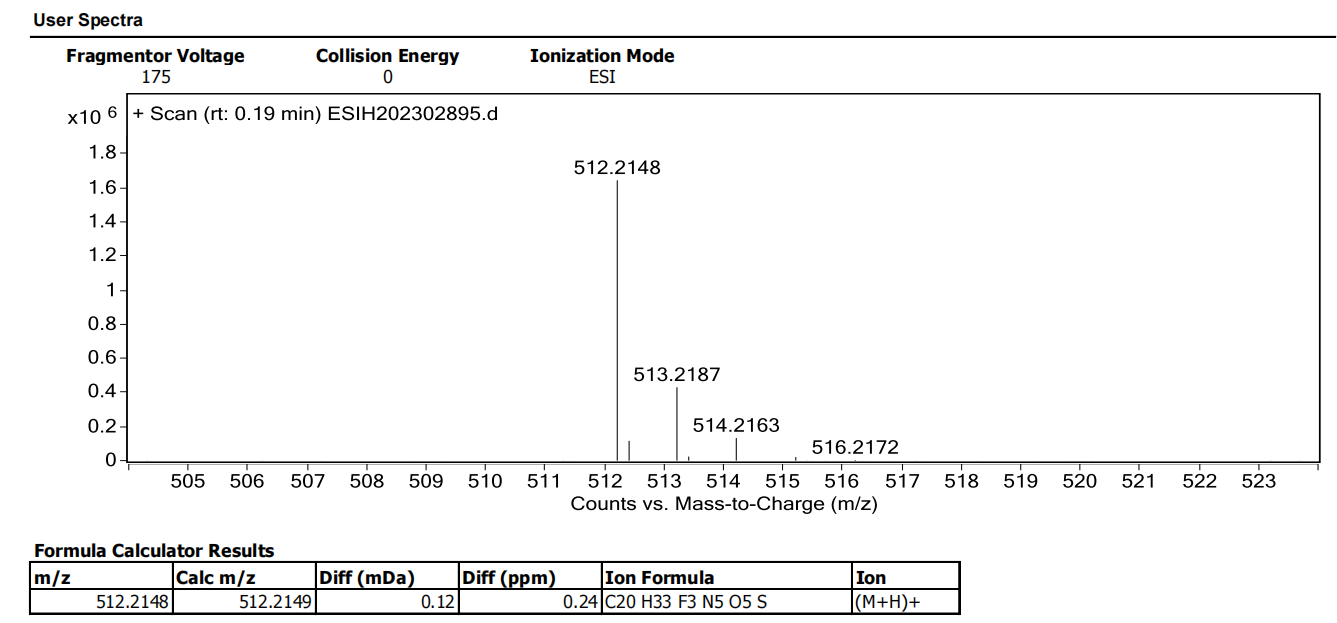
**

HPLC Trace for **5**

**
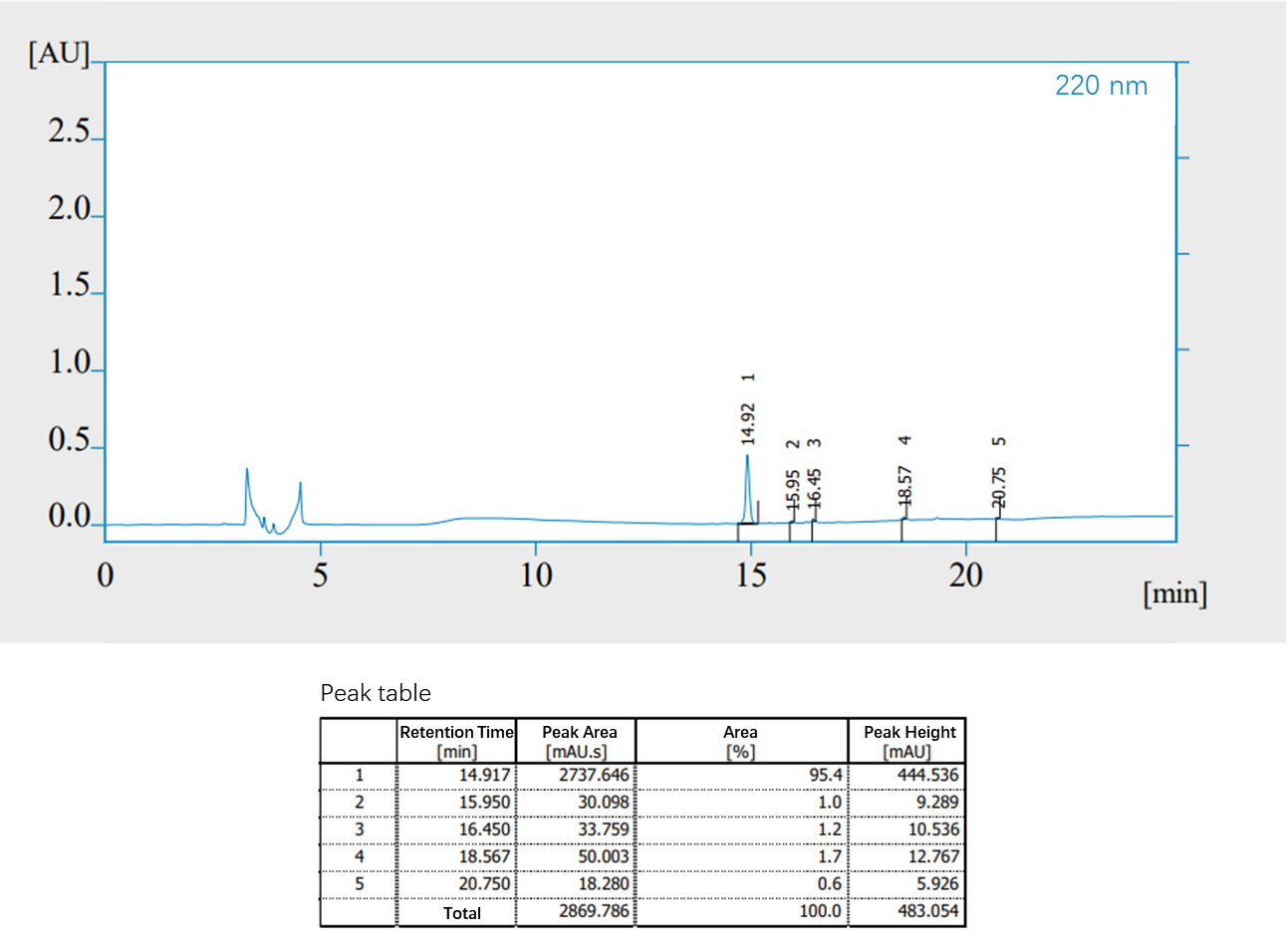
**

^1^H NMR Spectra for **6**

**
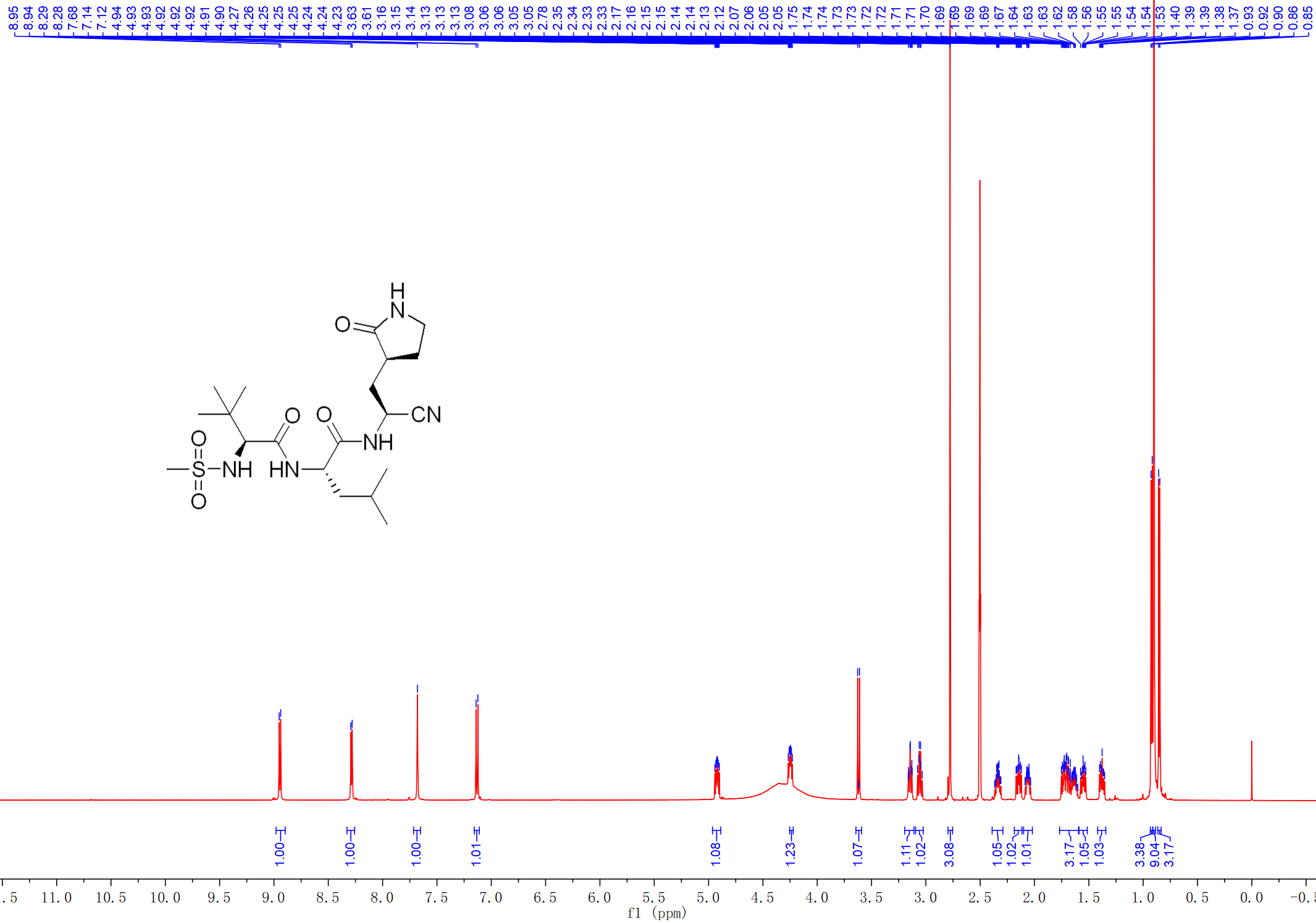
**

^13^C NMR Spectra for **6**

**
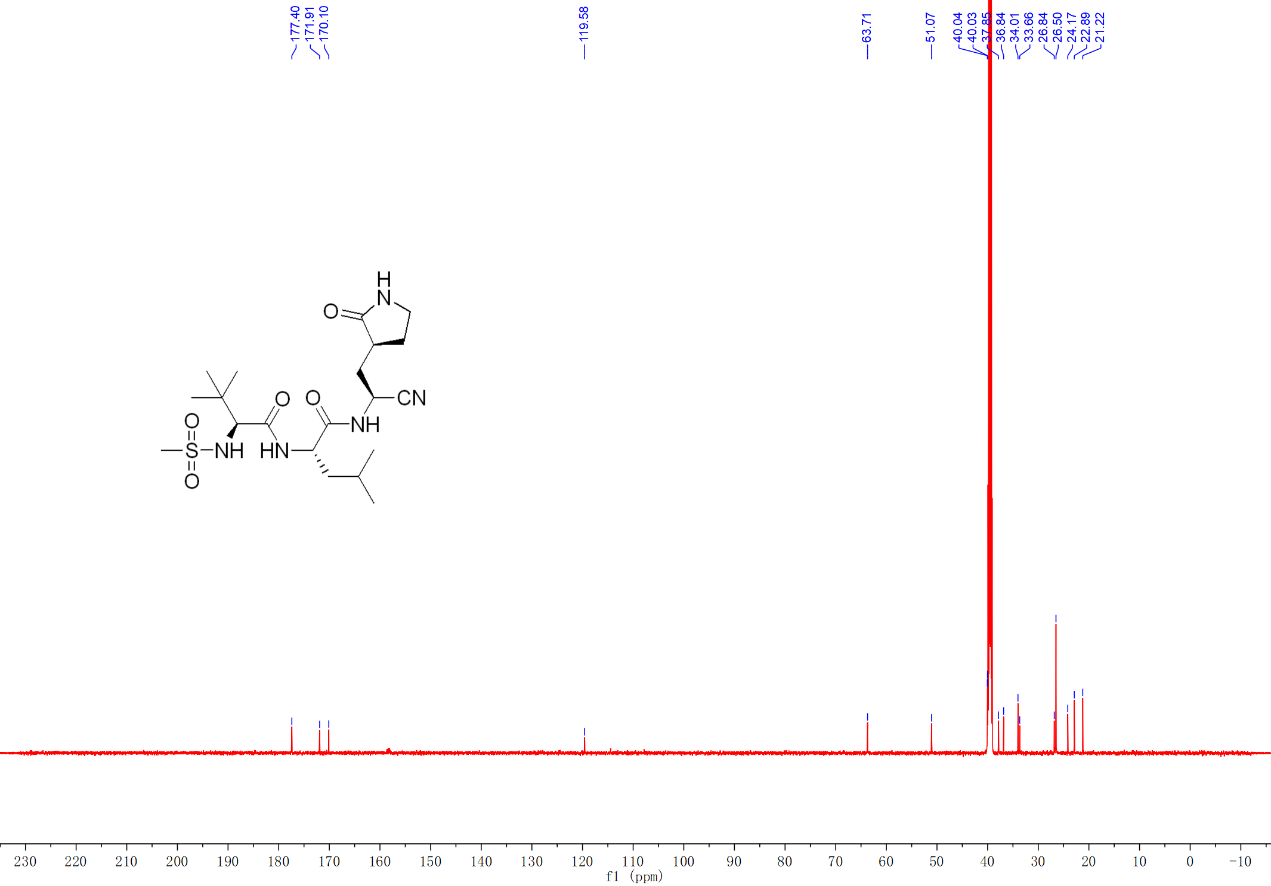
**

HRMS Spectra for **6**

**
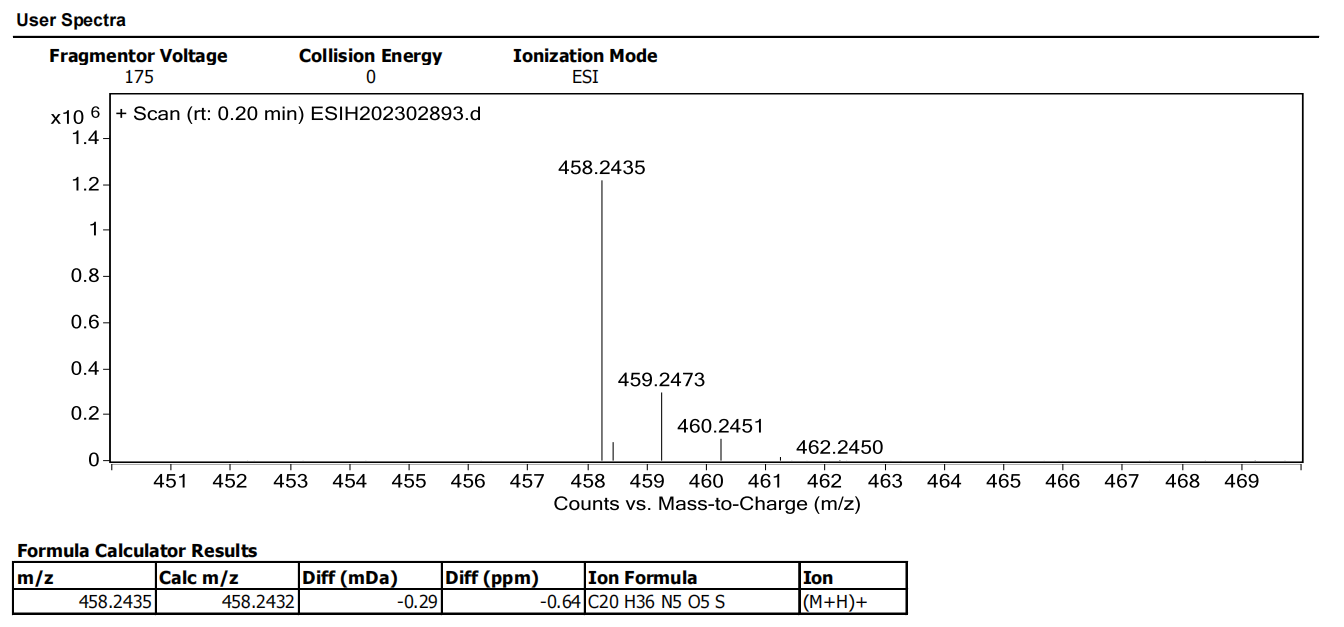
**

HPLC Trace for **6**

**
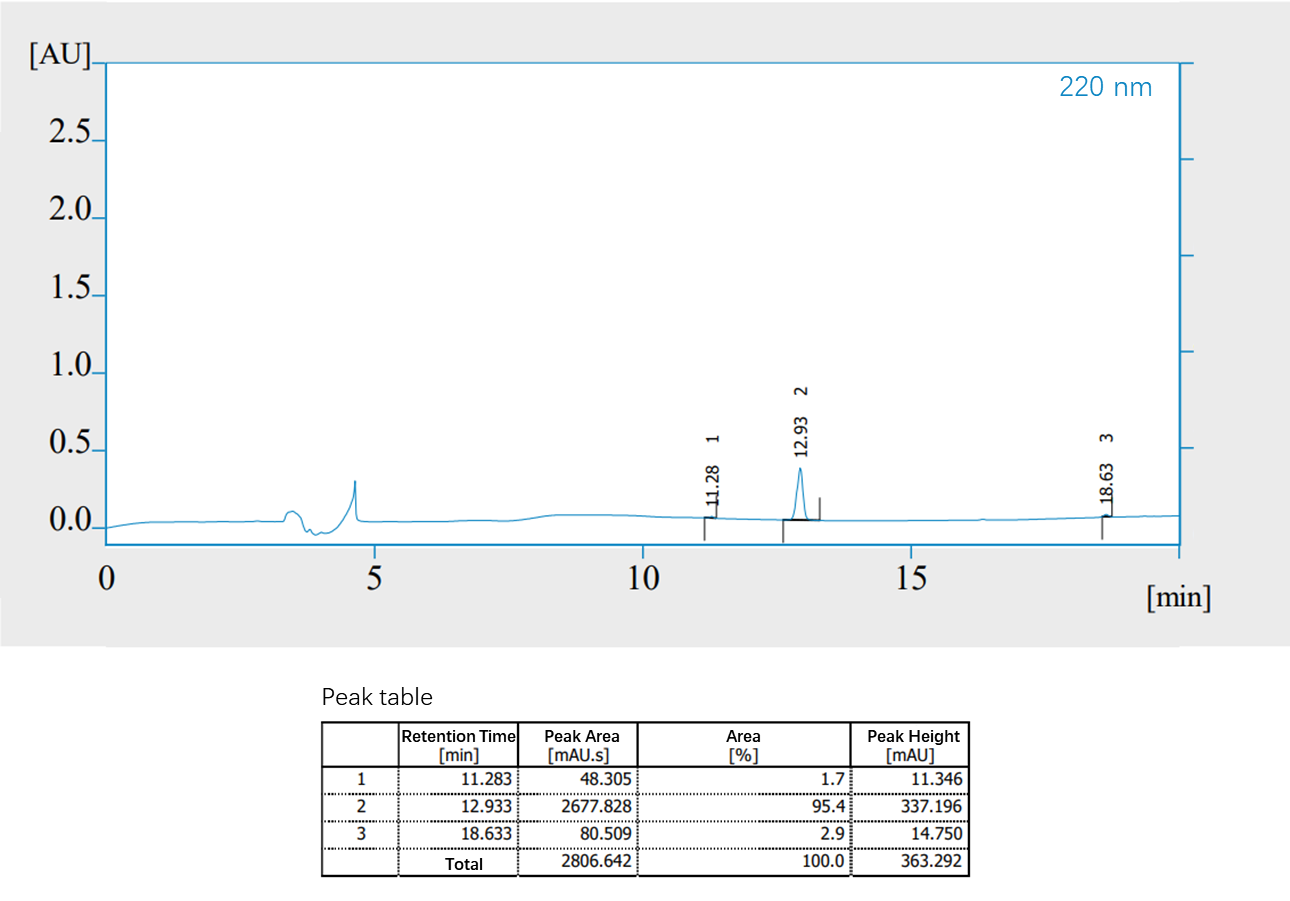
**

^1^H NMR Spectra for **7**

**
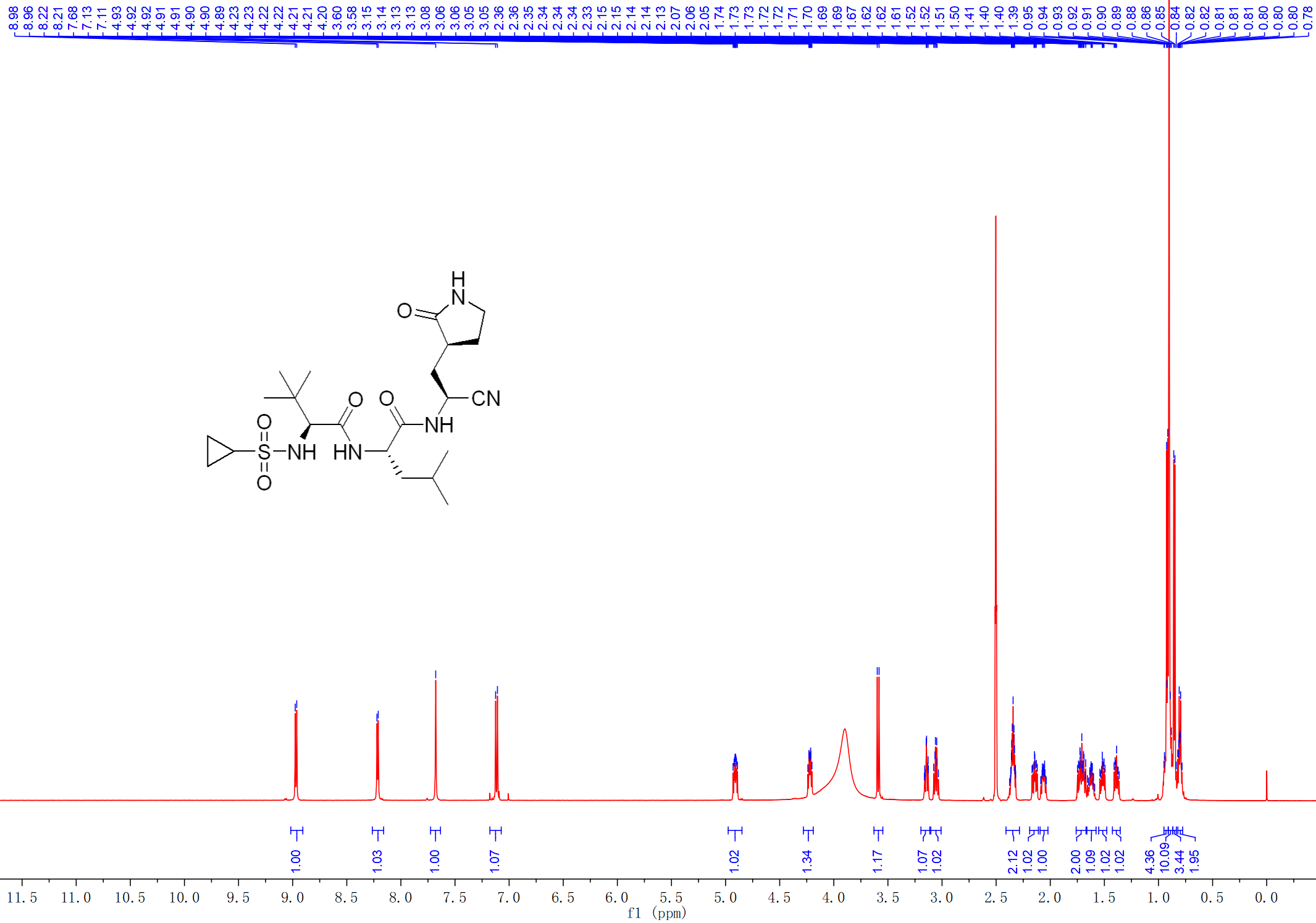
**

^13^C NMR Spectra for **7**

**
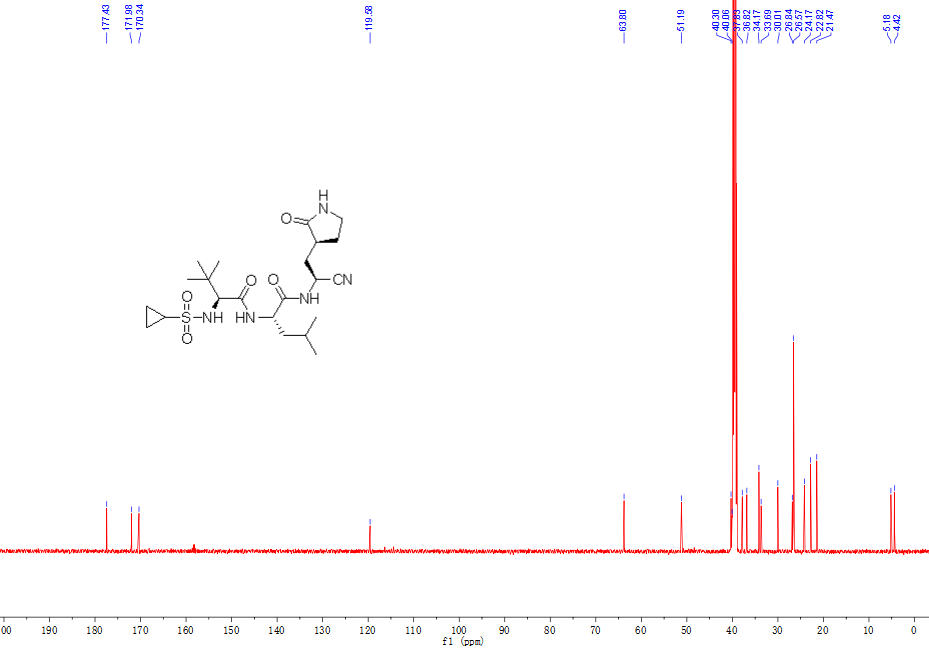
**

HRMS Spectra for **7**

**
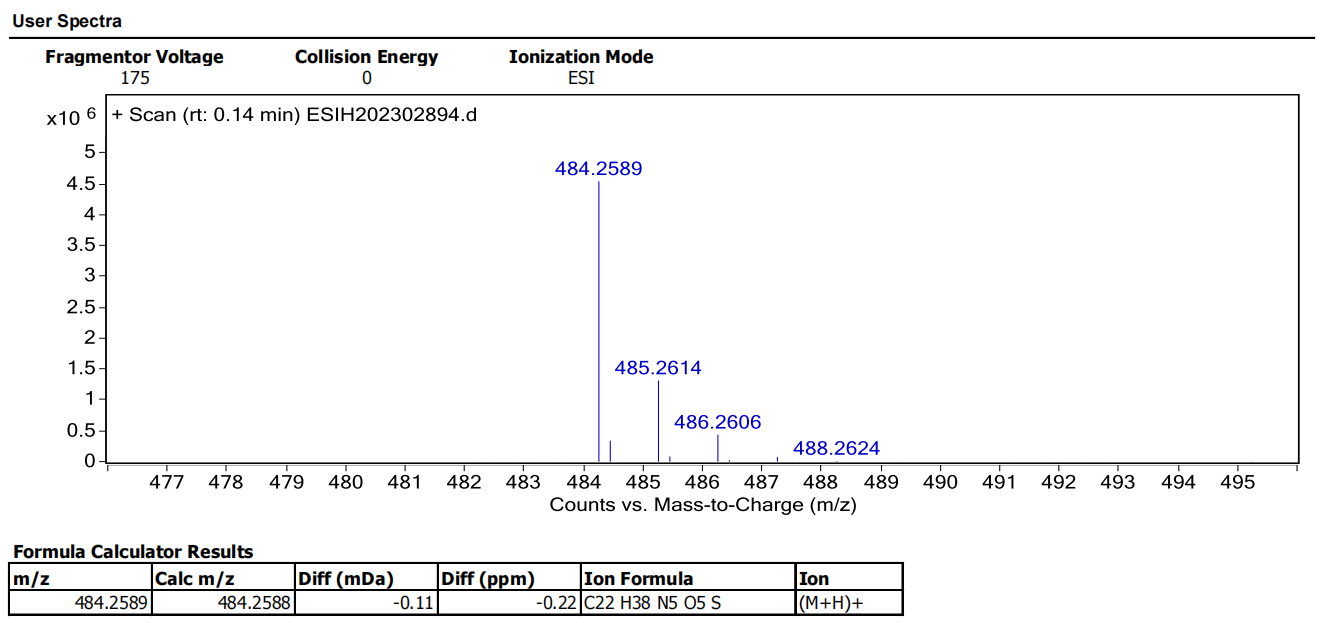
**

HPLC Trace for **7**

**
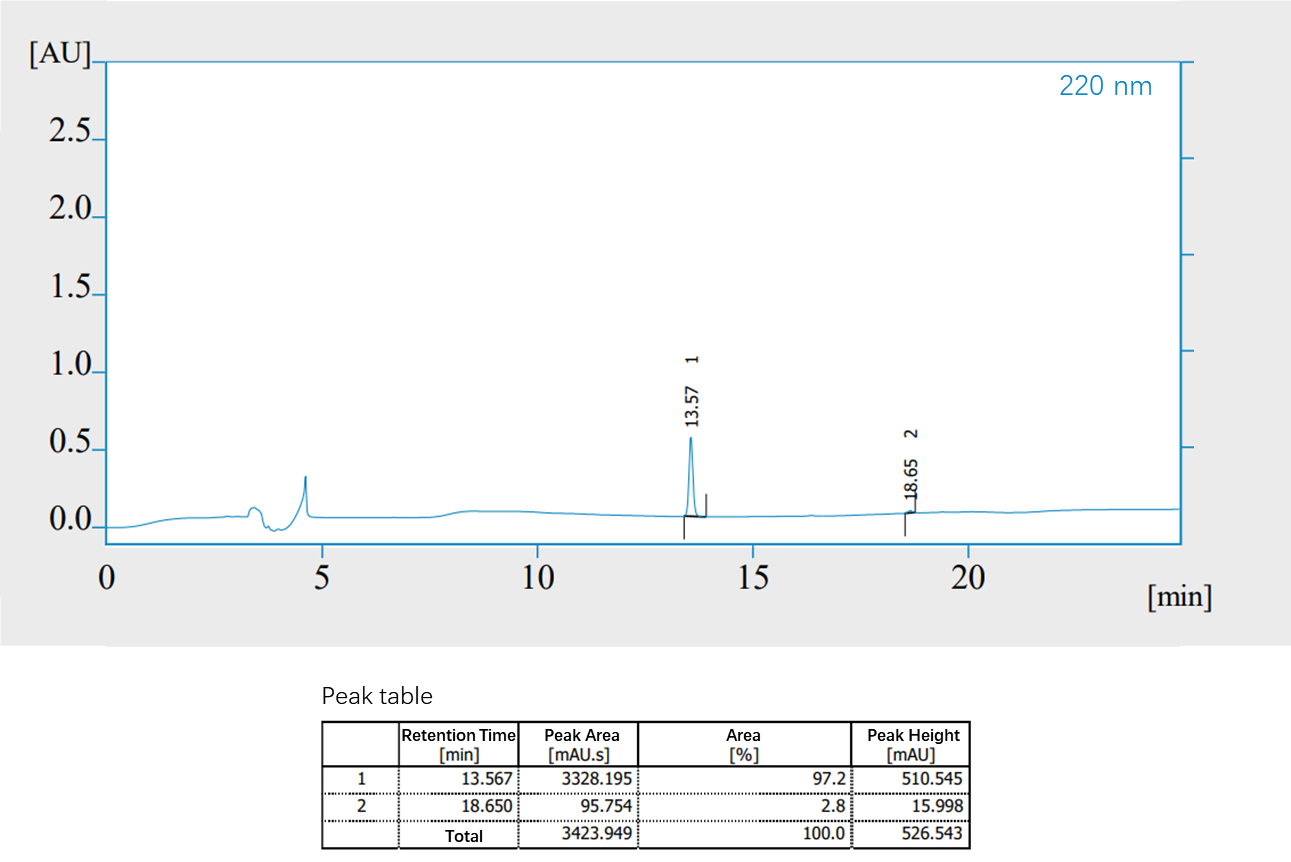
**

^1^H NMR Spectra for **8**


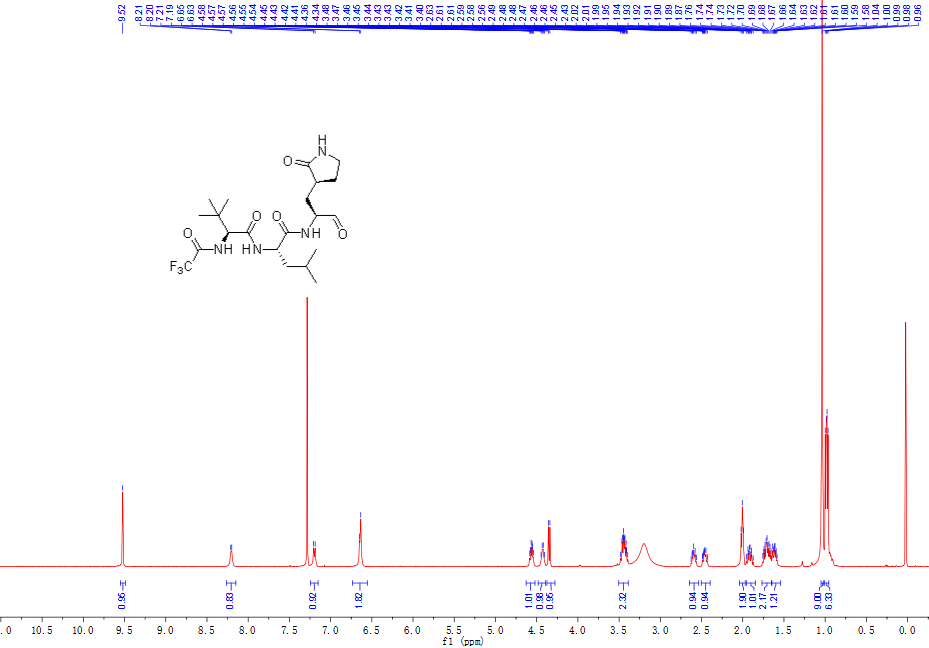


^13^C NMR Spectra for **8**


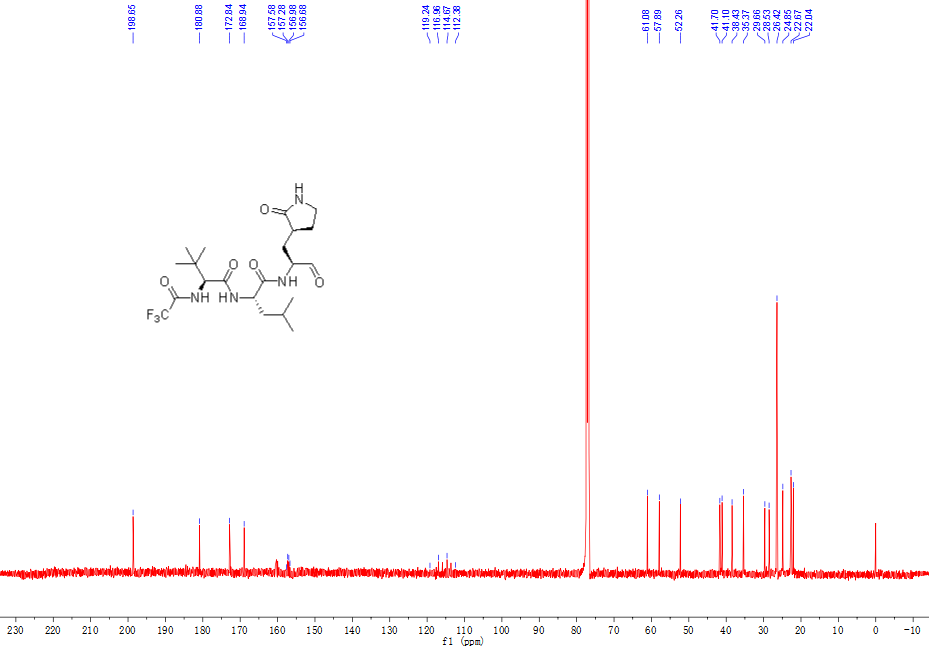


HRMS Spectra for **8**


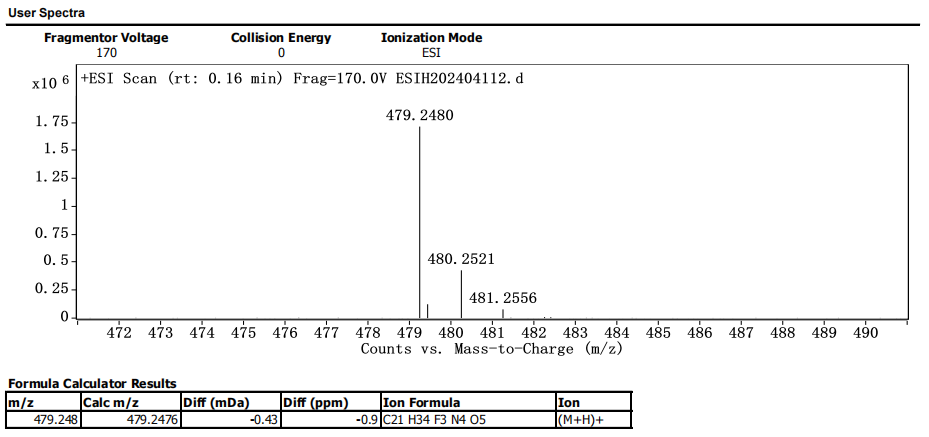


HPLC Trace for **8**


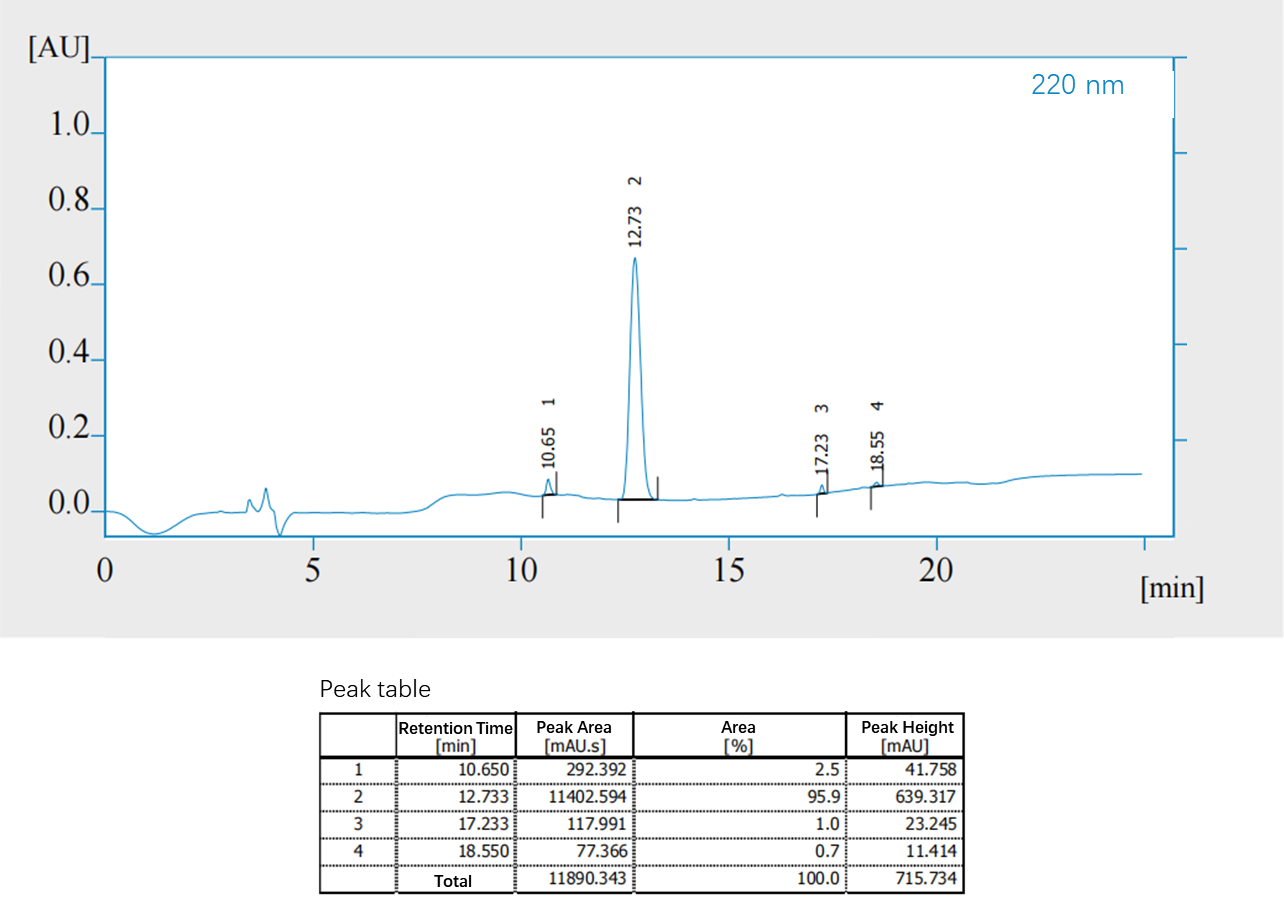


^1^H NMR Spectra for **9**


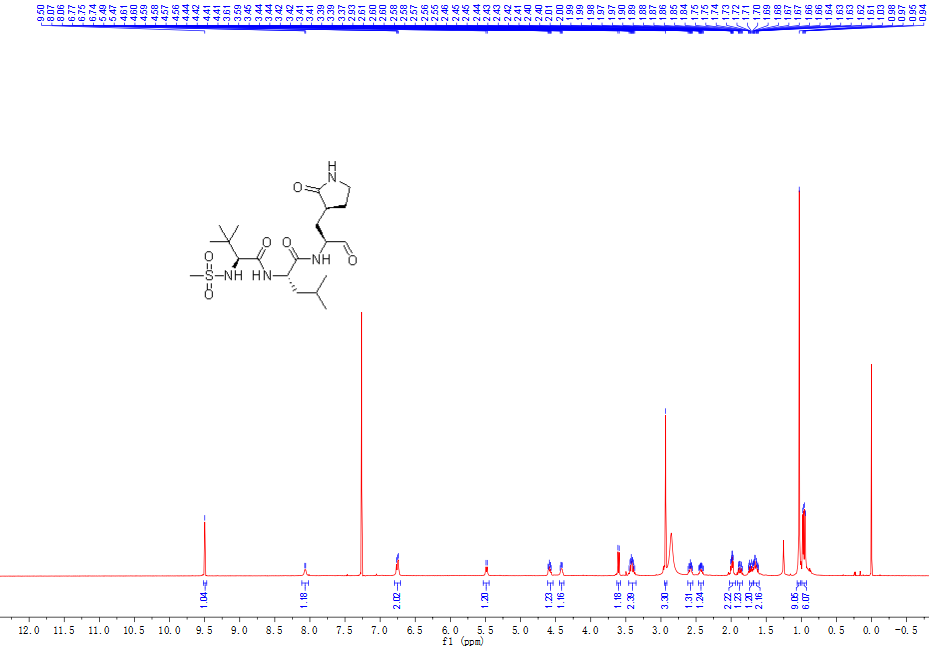


^13^C NMR Spectra for **9**


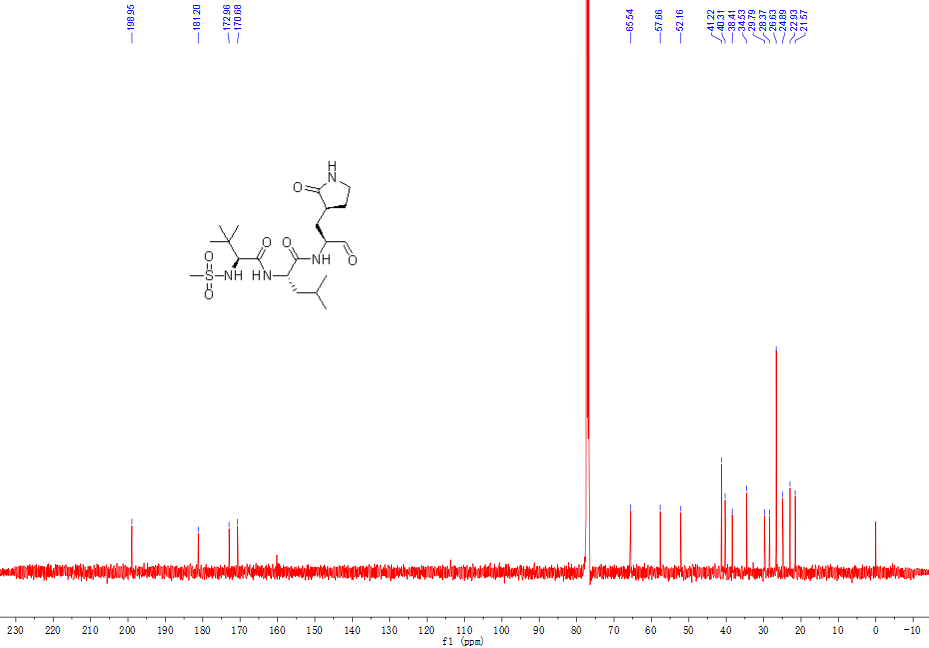


HRMS Spectra for **9**


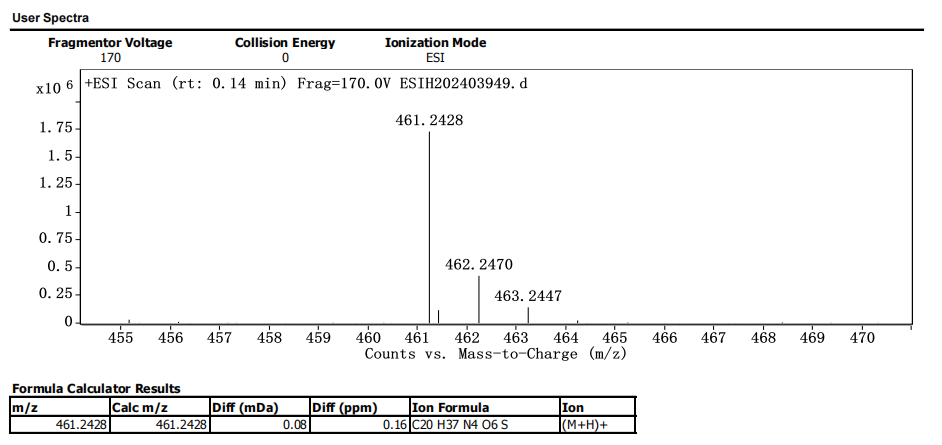


HPLC Trace for **9**


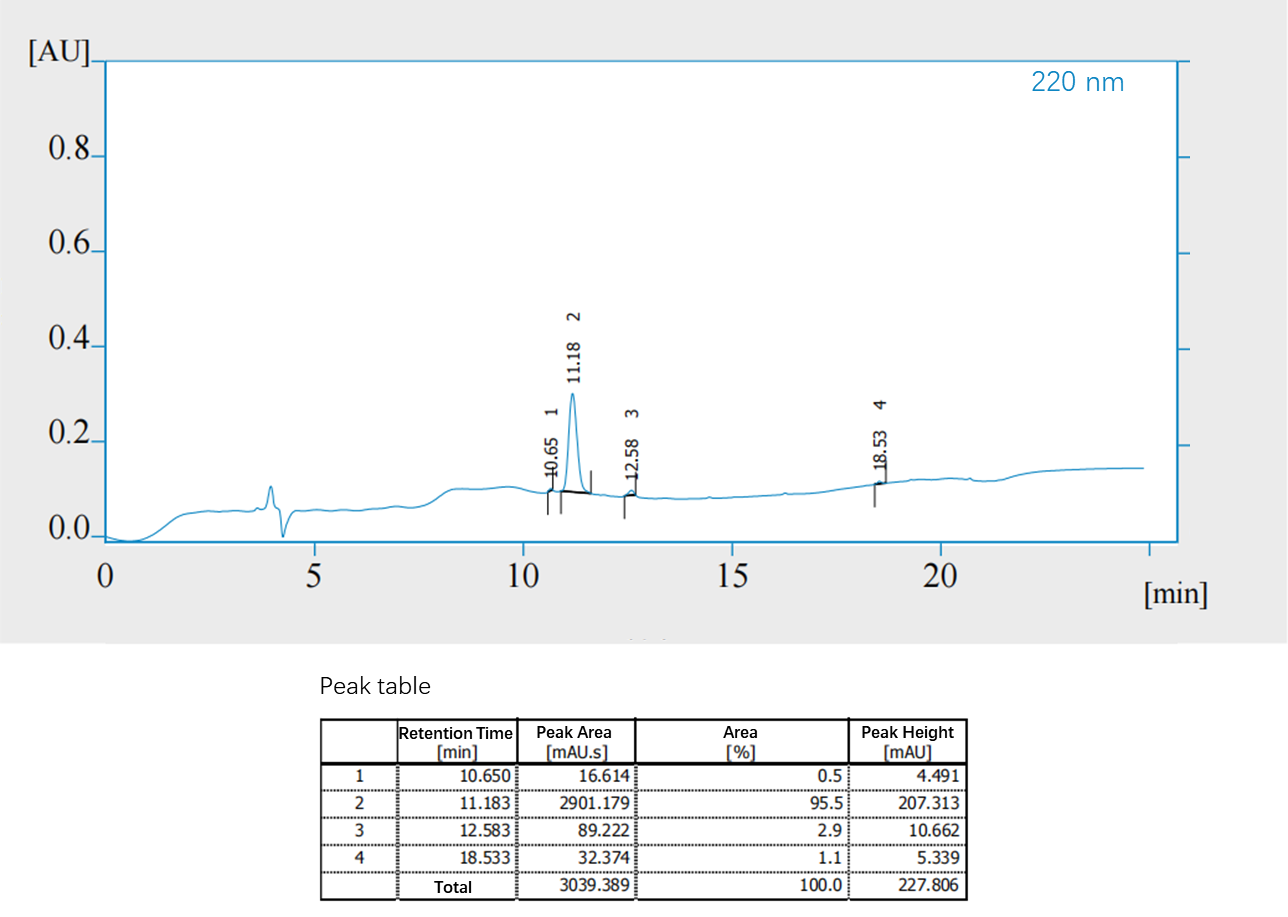


^1^H NMR Spectra for **10**

^
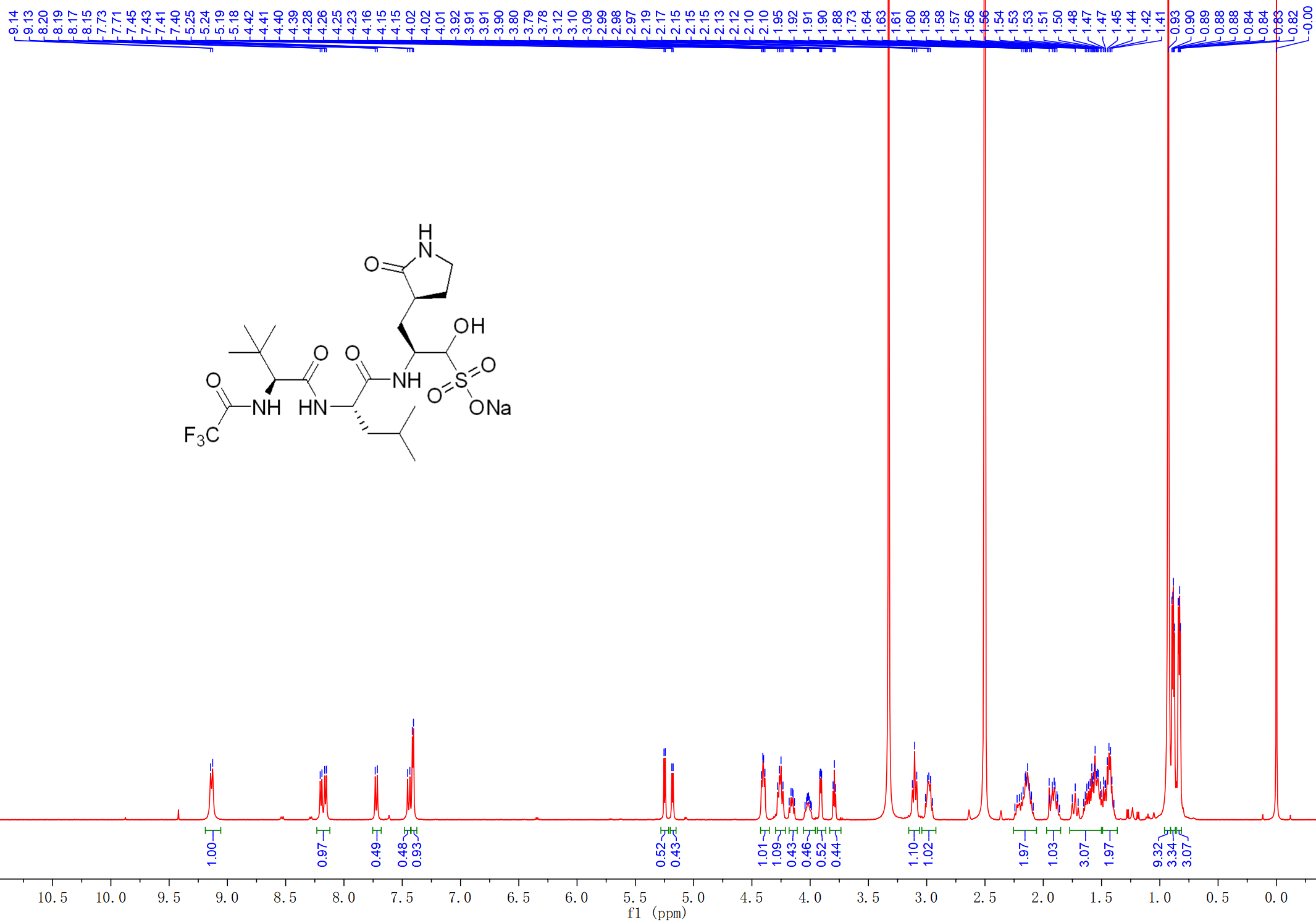
^

^13^C NMR Spectra for **10**


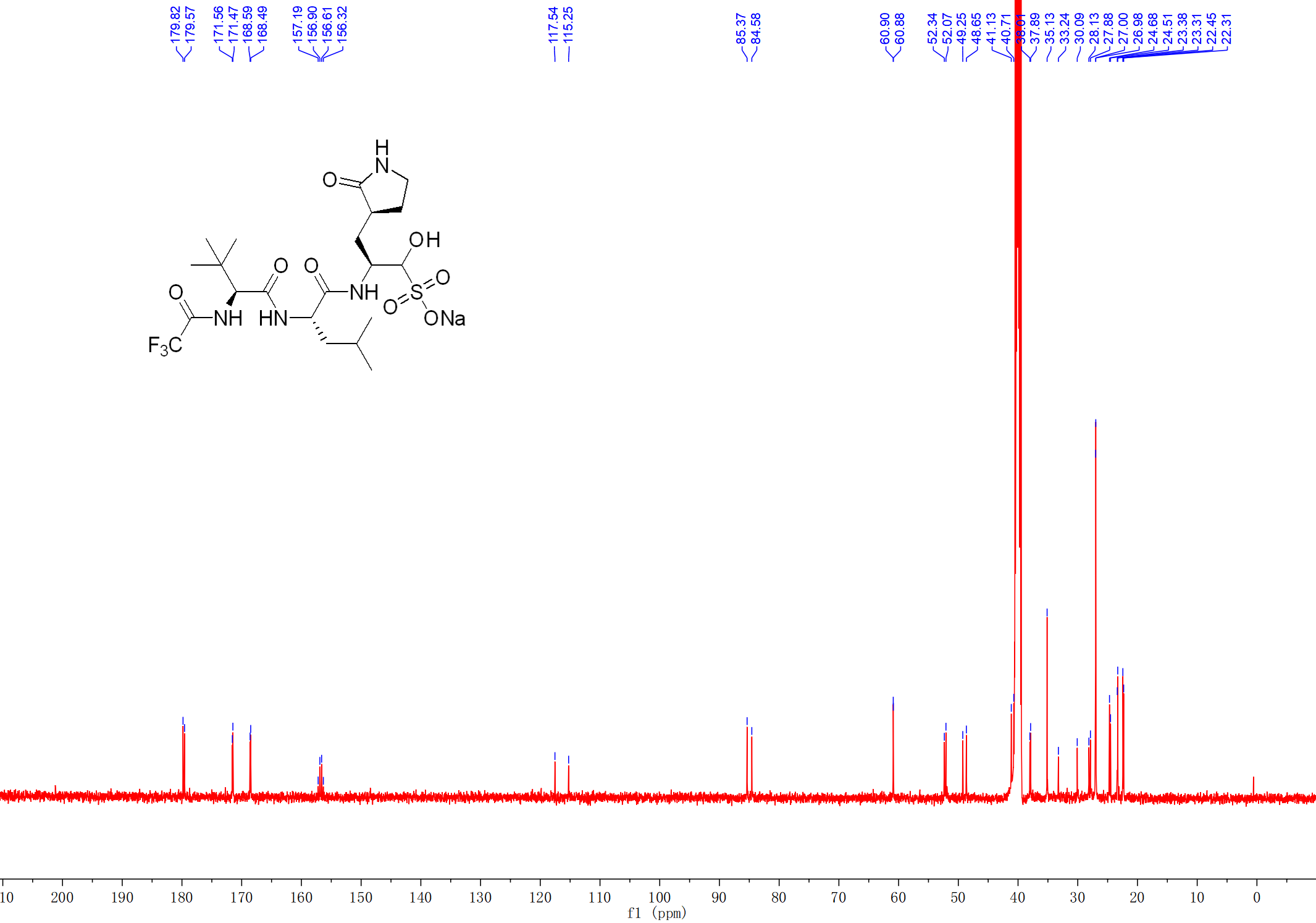


HRMS Spectra for **10**


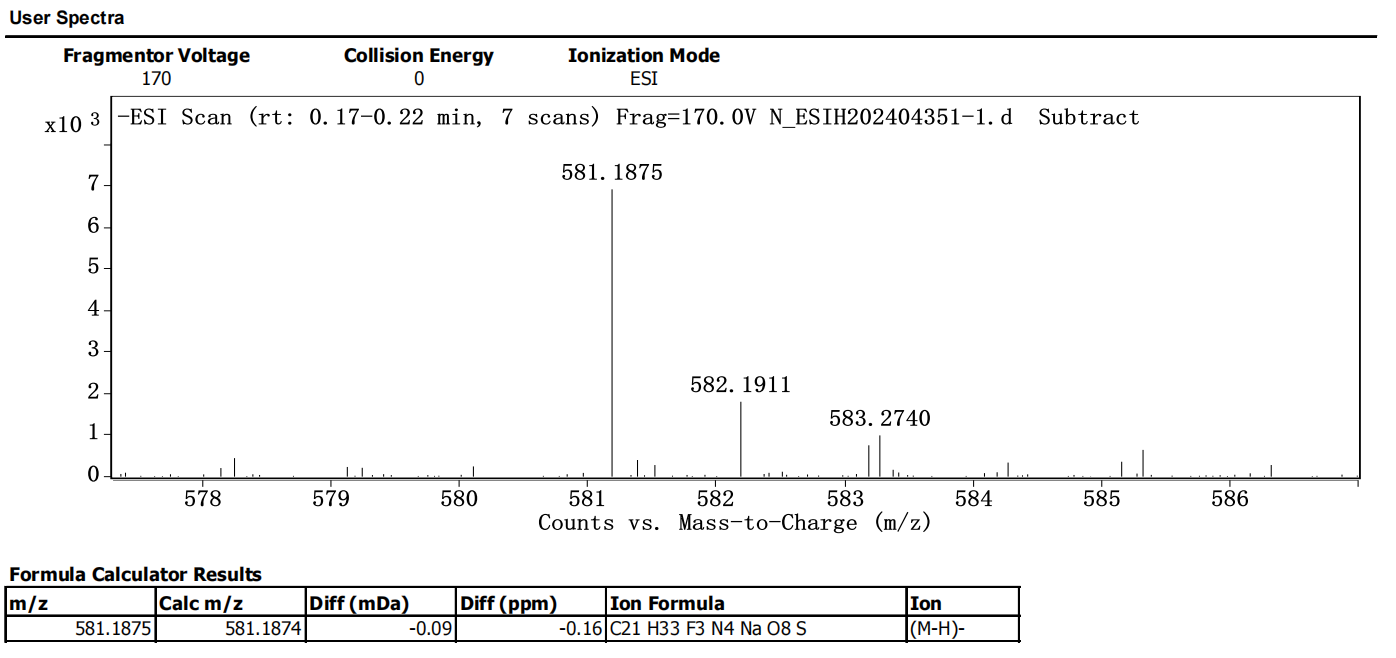

Supplement: Supplementary file 1 — Supporting Information [file ADVS-13-e12342-s001.docx]
